# Tranexamic Acid in Chronic Subdural Hematomas (TRACS): study protocol for a randomized controlled trial

**DOI:** 10.1186/s13063-016-1358-5

**Published:** 2016-05-05

**Authors:** Christian Iorio-Morin, Jocelyn Blanchard, Maxime Richer, David Mathieu

**Affiliations:** Division of Neurosurgery, Department of Surgery, Centre Hospitalier Universitaire de Sherbrooke, 3001, 12e avenue Nord, Sherbrooke, QC J1H 5N4 Canada; Department of Pathology, Centre Hospitalier Universitaire de Sherbrooke, 3001, 12e avenue Nord, Sherbrooke, QC J1H 5N4 Canada; Centre de recherche du CHUS, Sherbrooke, QC Canada

**Keywords:** Tranexamic acid, Cyklokapron, Conservative management, Medical management, Traumatic, Non-traumatic, Chronic subdural hematoma

## Abstract

**Background:**

Chronic subdural hematoma (CSDH) is one of the most frequent reason for cranial neurosurgical consultation. There is no widely accepted medical treatment for this condition. Herein, we present the protocol for the Tranexamic Acid (TXA) in Chronic Subdural Hematomas (TRACS) trial aiming at determining whether TXA can increase the rate of CSDH resolution following conservative management, lower the number of required surgical procedures and decrease the rate of CSDH recurrence following surgical evacuation.

**Methods:**

TRACS is a multicenter, double-blind, randomized, parallel-design, placebo-controlled, phase IIB study designed to provide preliminary efficacy data as well as feasibility, safety and incidence data required to plan a larger definitive phase III trial.

Consecutive patients presenting with a diagnosis of chronic subdural hematoma will be screened for eligibility. Exclusion criteria include: specific risk factors for thromboembolic disease, anticoagulant use or contraindication to TXA. A total of 130 patients will be randomized to receive either 750 mg of TXA daily or placebo until complete radiological resolution of the CSDH or for a maximum of 20 weeks. CSDH volume will be measured on serial computed tomography (CT) scanning. Cognitive function tests, quality of life questionnaires as well as functional autonomy assessments will be performed at enrollment, at 10 weeks following randomization and at 3 months following treatment cessation. During the treatment period, patients will undergo standard CSDH management with surgery being performed at the discretion of the treating physician. If surgery is performed, the CSDH and its outer membrane will be sampled for in vitro analysis.

The primary outcome is the rate of CSDH resolution by 20 weeks without intervening unplanned surgical procedure. Secondary outcomes include: CSDH volume, incidence of surgical evacuation procedures, CSDH recurrence, cognitive functions, functional autonomy, quality of life, incidence of complications and length of hospital stay. Planned subgroup analyses will be performed for conservatively versus surgically managed subjects and highly versus poorly vascularized CSDH.

**Discussion:**

CSDH is a frequent morbidity for which an effective medical treatment has yet to be discovered. The TRACS trial will be the first prospective study of TXA for CSDH.

**Trial registration:**

NCT ID: NCT02568124.

**Electronic supplementary material:**

The online version of this article (doi:10.1186/s13063-016-1358-5) contains supplementary material, which is available to authorized users.

## Background

Chronic subdural hematoma (CSDH) is one of the most frequent reasons for cranial neurosurgical consultation and a significant public health problem. The incidence in modern series ranges from 13.5 to 58.1 per 100,000 persons, depending on the studied population [1], and it peaks in the eighth decade.

As the name implies, CSDH results from venous bleeding in the space between the brain and the dura mater. While a major trauma is usually required to produce a subdural bleeding in young adults, a simple fall can be sufficient in the elderly, because brain atrophy results in stretching of bridging veins, rendering them susceptible to bleeding from minor trauma. In the acute stage, hemostasis is achieved through activation of coagulation cascades and formation of a blood clot. It is subsequently reorganized and reabsorbed following activation of the fibrinolytic cascade. For unknown reasons, however, this process often fails in the elderly, where an inflammatory reaction triggers the formation of a neovascularized membrane surrounding the clot [2]. It is postulated that activation of fibrinolysis within the hematoma might sustain a local coagulopathy which would promote low-volume bleeding from the outer membrane of the clot [3, 4]. This prevents resolution of the subdural collection, which then becomes “chronic”. Depending on its size, the hematoma can produce a variety of symptoms ranging from headaches and cognitive dysfunction to seizures, hemiplegia and coma. In most cases, motor symptoms and gait disruption will result in subsequent falls, which might lead to rebleeding [5]. Only 2.4 to 18 % of CSDH will resolve spontaneously [6] and most patients will require surgical drainage. In frail, elderly patients, this can be non-trivial.

There is no widely accepted medical treatment for CSDH. Evidence is currently available for four classes of drugs: corticosteroids, angiotensin-converting enzyme (ACE) inhibitors, platelet-activating factor (PAF) receptor antagonists and antifibrinolytics.

### Corticosteroids

Corticosteroids have been proposed as a way to reduce inflammation and angiogenesis in mild CSDH cases [2]. A recent meta-analysis of CSDHs found 5 studies investigating corticosteroids as the primary management strategy and 17 studies assessing its impact as an adjuvant to surgery [7]. Pooled analysis found no benefit and an increased morbidity when corticosteroids are used. Multiple prospective randomized controlled trials are currently ongoing to clarify the issue (NCT02111785, NCT02192320, NCT01380028).

### ACE inhibitors

In 2007, a retrospective analysis of 438 patients who underwent surgery for CSDH suggested that concomitant use of an ACE inhibitor for the management of hypertension lowered the risk of CSDH recurrence following surgery [8]. Indeed, ACE inhibitors have known antiangiogenic properties and might inhibit neovascularization in the CSDH outer membrane. A prospective randomized controlled trial comparing the effect of perindopril on postoperative recurrence has just been completed and is pending publication (NCT00915928).

### Platelet-activating factor receptor antagonist

Platelet-activating factor has been shown to be a potent lipid mediator of the inflammation involved in the formation and growth of the CSDH membrane [9]. Two small prospective studies from a single group demonstrated that treatment with the PAF receptor antagonist etizolam decreases the need for surgery [10] and decreases CSDH recurrence after surgery [11]. No further study is currently announced despite these interesting results.

### Antifibrinolytics

Tranexamic acid (TXA) is an antifibrinolytic agent used to minimize bleeding in trauma patients [12], high-risk surgical procedures [13] and refractory menorrhagia [14]. Its use in the treatment of CSDH was reported in two papers. The first is a case report of a hemodialyzed patient with CSDH refractory to three surgical evacuations [15]. The patient was given TXA (20 mg/kg/48 h intravenously (IV) for 4 weeks, followed by 10 mg/kg/48 h per os (PO) for 4 weeks) which led to complete radiological resolution of the CSDH. The patient remained recurrence-free at the 1-year follow-up. The second paper is a Japanese case series of 21 consecutive patients treated with TXA (750 mg PO daily) until radiological CSDH resolution on follow-up computed tomography (CT) scans performed every 3 weeks [16]. The authors report a 100 % success rate with a median treatment duration of 58 days (range 28–137 days).

While these anecdotal reports suggest a role for TXA in the medical management of CSDH, no interventional trial on the matter has been published. As of 14 November 2014, a search of the International Committee of Medical Journal Editors (ICMJE)-approved trial databases (including, “ClinicalTrials.gov,” “ISRCTN.org,” “anzctr.org.au,” “umin.ac.jp/ctr/index/htm,” “trialregister.nl” and “eudract.ema.europa.eu”), the World Health Organization (WHO) International Clinical Trials Registry Platform and PubMed yielded no planned or ongoing trial of TXA in CSDH. Because of the strong pathophysiological rationale for the use of TXA in CSDH and the encouraging reports previously published, we believe that further investigation of the matter is indicated.

### Objective

We will be conducting a phase IIB trial, called “Tranexamic Acid in Chronic Subdural Hematomas” (TRACS), to investigate whether TXA can increase the rate of CSDH resolution following conservative management, lower the number of required surgical procedures and decrease the rate of CSDH recurrence following surgical evacuation. The TRACS trial will provide preliminary efficacy data as well as the safety and incidence data required to plan a larger definitive phase III trial. The trial registration ID is NCT02568124. This paper is a summary of the study protocol adhering to the Standard Protocol Items: Recommendations for Interventional Trials (SPIRIT) Statement [17] (Additional file 1). The full, original protocol is available as Additional file 2 (French version only).

## Methods

### Overview

TRACS is a double-blinded, randomized, parallel-design, placebo-controlled trial investigating the effect of long-term (up to 20 weeks) TXA administration on CSDH resolution when used as an adjunct to standard management. A total of 130 adult CSDH patients will be randomized to receive either TXA or placebo. Hematoma resolution will be assessed by serial CT scanning.

### Inclusion criteria

TRACS will be open to adult patients (18 years of age or older) for whom a recent (up to 14 days) CT scan demonstrates the existence of a subdural hematoma containing a chronic component.

- A subdural hematoma is defined as a measurable liquid and hemorrhagic collection within the subdural space as assessed by CT scanning
- A chronic component is defined as a hypodense (<25 Hounsfield units (HU)) or isodense (25–35 HU) region within the subdural collection in the absence of anemia or coagulopathy
- In the presence of anemia or coagulopathy, at least one additional sign suggestive of a chronic process must be seen. Acceptable signs include:
  - ○ Bilateral hematomas
  - ○ Loculation within the hematoma
  - ○ Visible membrane surrounding the hematoma

Subjects presenting with an acute rebleeding within a CSDH are eligible.

### Exclusion criteria

Patients will be excluded from the study in the following cases:

- Acute subdural hematoma with no chronic component
- Active thrombotic, thromboembolic or atheroembolic disease, including:
  - ○ Deep venous thrombosis within the last 6 months
  - ○ Cerebral thrombosis within the last 6 months
  - ○ Symptomatic carotid stenosis that did not undergo surgery
  - ○ Stroke (ischemic or hemorrhagic) within the last year
  - ○ Acute coronary syndrome within the last year;
- Past history of unprovoked deep venous thrombosis or idiopathic pulmonary embolism
- Known hereditary thrombophilia including:
  - ○ Factor V Leiden
  - ○ Antithrombin III mutation
  - ○ Protein C deficiency
  - ○ Protein S deficiency
- Atrial fibrillation (unless under successful rhythm control therapy)
- Metallic heart valve
- Vascular stenting procedure within the last year
- Cardiac or vascular surgical procedure within the last 6 months, including:
  - ○ Endarterectomy
  - ○ Bypass
  - ○ Angioplasty
- Ongoing investigation for suspected malignancy
- Confirmed active malignancy
- Concomitant hormone therapy for malignancy
- Concomitant use of a hormone contraceptive pill
- Macroscopic hematuria
- Known or suspected TXA allergy
- Pregnancy or breastfeeding
- Concomitant use of anticoagulant medication
- Any concern from the attending physician

These extensive exclusion criteria have been selected to provide maximum safety for the study subjects. It is likely that our patient population will be significantly older and at higher risk for thromboembolic complications than in most other studies of TXA, notably the trauma studies [12]. Until the effectiveness of TXA use in CSDH is demonstrated, we believe that any risk factor for thrombotic events should be carefully considered. Should any exclusion criteria be newly diagnosed during the course of the study, treatment will be discontinued, although follow-up will proceed as planned.

### Setting and screening

TRACS is a Canadian multicenter trial. The coordinating center is the Centre Hospitalier Universitaire de Sherbrooke (CHUS). Enrollment will take place at the CHUS and at the Centre Hospitalier Affilié Universitaire de Québec. In both centers, all consecutive patients presenting to the neurosurgery department with a diagnosis of a subdural hematoma with a chronic component will be screened for eligibility by the treating physicians. It is likely that some eligible patients will be missed because of physician oversight. However, this screening method was deemed more sensitive than a systematic review of the participating centers’ daily head CT reports, because a significant proportion of patients are referred for consultation based on head CT scans performed in outside community hospitals. A screening log will be kept. Following screening, interested and potentially eligible patients will meet a trained research nurse who will consent and enroll the subjects.

### Power calculation

The primary goal of TRACS is to generate the feasibility, safety and incidence data required to inform the design of a larger definitive phase III trial. Powering for efficacy is, therefore, not required. However, given the impressive effect size reported in the case series, demonstration of efficacy might be achievable even within this smaller phase II study.

At our institution (CHUS) between 2009 and 2012, administrative data suggests that about 30 % of CSDH were symptomatic and underwent surgical treatment. This amounted to about 28 surgical procedures performed annually when including reoperations (unpublished data). In the literature, rates of complete radiological resolution following surgery have been reported to be between 70 and 95 % [18] with only 3–18 % of cases resolving spontaneously if conservative management is undertaken [6]. Using these data, if we postulate that 30 % of patients enrolled in the study will undergo surgical evacuation, the primary outcome in the placebo group should be met in 23–41 %. We believe that the effect of TXA would be clinically significant if it can double the rate of CSDH resolution. Given those parameters, obtaining an 80 % power with a statistical significance threshold set at *p* = 0.05 would require each treatment arm to recruit between 21 (if the primary outcome in the placebo group is met in 41 % of subjects) and 65 subjects (if the primary outcome in the placebo group is met in 23 % of subjects). The initial recruitment goal will, therefore, be of 65 subjects per group, for a total of 130 analyzed patients. With two recruiting centers, this goal could be achieved within 2.5 years if more than 40 % of screened patients are eligible and consent to participate.

This power analysis is based on a number of assumptions, including the incidence of the primary outcome, the size of the treatment effect, the proportion of patients undergoing planned surgical evacuation and the rate of change from planned conservative to surgical management (which leads to a failure of meeting the primary outcome). This can significantly impact the power of the study. Since 100 % of the 22 cases published achieved complete CSDH resolution [15, 16], powering for an incidence of primary outcome of 46–82 % in the treatment arm (odds ratio of 2) seems reasonable. A lower odds ratio could still be clinically significant given the reported safety of TXA, although detecting it would require a substantially larger cohort (225 subjects per group for a 1.5 odds ratio with a 23 % rate of primary outcome in the placebo group). To verify these assumptions, an interim power analysis will be performed after follow-up data is available for the 65th subject and using the study’s real primary outcome incidence. Target sample size will be adjusted if this analysis suggests that the power required to assess effectiveness could be reached with available or minimal extension of funding. However, while TRACS is designed to provide preliminary efficacy data, the main objective is to provide the feasibility, safety and incidence data required to plan a larger definitive phase III trial. Therefore, powering for effectiveness is not strictly required.

### Ethics, consent and permissions

Study explanation to potential subjects will be performed by research personnel independent from the treating physicians. Consent will be obtained from the patient directly whenever possible. However, it is expected that a significant proportion of patients in the target population will be unable to give informed consent because of confusion or speech impairment caused by their condition. In such cases, consent will be obtained from a legal representative or the patient’s most significant relative. Consent to remain in the study will be obtained from the subject as soon as competency to consent is recovered. This process is in accordance with the World Medical Association’s Declaration of Helsinki [19]. The TRACS trial has received local Institutional Review Board (IRB) (Comité d’éthique de la recherche du CR-CHUS) approval as well as approval from Health Canada. The French and English consent forms are available as Additional files 3 and 4.

### Stopping guidelines

The safety profile of TXA is well-established and the probability of harm resulting from study participation is low. Because TRACS is a small study, no predefined stopping guidelines have been included in the protocol. All complications will be reviewed by the principal investigator as they occur and by the safety monitoring committee at a planned meeting scheduled after follow-up data is available for the 65th subject. Should any concern arise, the data will be submitted for evaluation by the IRB and further enrollment stopped until a decision is made.

### Randomization, blinding and unblinding

Upon giving informed consent, subjects will be randomized by the recruiting center’s central pharmacy to one of two groups: a group treated with TXA and a control group treated with a placebo. Stratified block randomization will be used with a block size of 4 and the use of antiplatelet drugs within the last month and recruiting center as covariates. The allocation sequence, produced by an independent statistician, will be determined by assigning a random number to each subject within a block (two taking placebo and two taking TXA) and then sorting the subjects by number. Four tables of 128 allocations will be generated with Table A used for patients not previously taking antiplatelet therapy and Table B used for patients previously taking antiplatelet therapy. Tables C and D will be similarly generated and reserved for the second recruiting center. Subjects and care providers will be blinded by the use of a placebo tablet. In order to minimize cost, the placebo tablet will not be perfectly identical to the TXA tablet and the drugs will, therefore, be served in an opaque container to further protect the allocation from the physician. Blinding will be maintained throughout the trial period, including during the interim analysis where patient’s group assignation will be unmasked, but not the group’s assignation to a specific treatment. After database lock following the last follow-up of the last patient, planned statistical analyses will be performed and conclusions drawn and presented to the trial steering committee prior to unblinding of the groups’ allocation.

There should be no need for unblinding during the treatment phase. If, for any patient, an adverse event occurs such as a thrombosis, pseudo-nephrolithiasis or suspected allergic reaction, the trial intervention should be stopped for the affected patient and standard supportive care given. Should the attending physician require unblinding, the coordination center’s pharmacy can be contacted at any time.

### Intervention

**Fig. 1:**
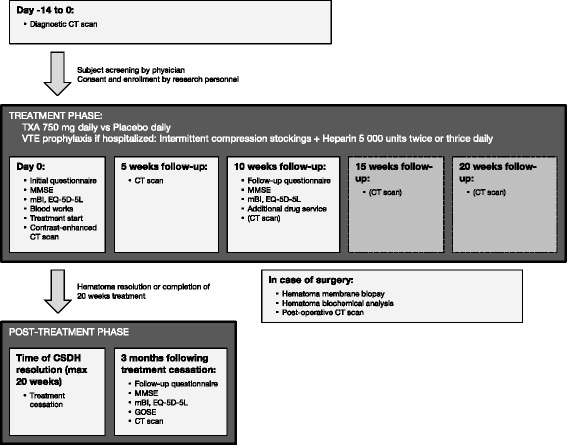
Intervention schedule. The TRACS trial is divided into three phases. The treatment phase is entered upon enrollment. The post-treatment phase is entered upon complete radiological resolution of the chronic subdural hematoma (CSDH) on follow-up computed tomography (CT) scans or after 20 weeks of treatment. The optional surgical phase is entered if the subject undergoes surgical CSDH evacuation

TRACS is divided into three phases: (1) the treatment phase, (2) the post-treatment phase, and (3) the optional surgical phase (see Fig. 1).

The treatment phase begins immediately after randomization and involves the administration of daily oral TXA or placebo. Planned procedures include blood tests at 0 weeks, a set of questionnaires and cognitive function tests at 0 and 10 weeks and serial CT scans every 5 weeks. The first CT scan will be contrast-enhanced to assess the degree of membrane vascularization. Throughout the treatment phase and regardless of treatment arm assignation, CSDH management proceeds at the discretion of the attending physician. This can include a conservative observation period, a surgical evacuation or any procedure deemed appropriate by the medical team. Resumption of prior antiplatelet medications is not standardized but is documented. The treatment phase continues until complete radiological resolution of the CSDH or for a maximum of 20 weeks.

The post-treatment phase begins at the first CT scan demonstrating complete radiological resolution of the CSDH or after 20 weeks of treatment. It involves the cessation of trial drug administration and a set of questionnaires, cognitive function tests and a CT scan to be performed after 3 months following treatment cessation.

If a surgical evacuation is performed, the optional surgical phase is entered and mandates sampling and analysis of the hematoma and its outer membrane, as detailed in the Outcomes section. Surgical technique is left to the discretion of the treating team. Timing of surgery, use of drains and use of burr holes versus craniotomy will be recorded, but are not pre-specified.

Treatment adherence will be monitored by asking patients to return any remaining drug tablets at each clinical visit and at the end of the study. The schedule of enrollment, interventions, and assessments is summarized in Fig. 1.

#### Dose selection

TXA has been used for many applications and the doses studied vary widely. Loading doses of 2.5–100 mg/kg and maintenance doses of 0.25–4 mg/kg/h have been reported as safe and effective in surgical patients, although total treatment duration in these studies was less than 12 h [20]. Approved dosing for menorrhagia is 1300 mg three times daily for up to 5 days. Off-label, long-term use of 500 mg/dose PO once or twice daily has been reported for hereditary angioedema prophylaxis with some patients treated safely for years [21].

CSDH is believed to result from a slow, occult bleeding from the subdural hematoma membrane. The goal of an antifibrinolytic therapy in this context is to correct the coagulopathy within the hematoma so that thrombosis of the surrounding membrane can occur and be followed by CSDH reabsorption. Because this process is unlikely to take place within 12 h, the use of a long-term, low-dose regimen might be favored. In the first case report of TXA use for CSDH, a dose of 20 mg/kg/48 h IV for 4 weeks, followed by 10 mg/kg/48 h PO for 4 weeks was administered [15]. For a 70-kg subject, this would yield 700 mg IV daily for 4 weeks, followed by 350 mg PO daily for 4 weeks. For all the other reported patients, a dose of 750 mg PO daily was used [16]. Because of the high effectiveness and absence of adverse event in this series, the latter dose was chosen for the TRACS trial.

TXA and placebo will be provided as tablets to be taken orally. The drug can be administered through a nasogastric tube if the patient is dysphagic.

#### Drug manufacture and supply of trial treatment

TXA and placebo drugs will be purchased on the open market by the trial coordinating center pharmacy (Centre de recherche du CHUS). The pharmacy will also manage the blinding process and drug packaging. As long as subjects are hospitalized, the drugs will be administered by the ward nurses along with the patient’s other medication. Upon discharge, remaining doses will be packaged and given to the patient with detailed written administration instructions. The initial trial drug service will cover the first 10 weeks of treatment. If, at the 10-week follow-up, the control CT scan demonstrates persistence of the CSDH, a second service of trial drugs will be performed and the treatment will be continued for a total of 20 weeks. Complete radiological resolution of the CSDH on any CT scan, at any time during the treatment phase, will end the trial intervention and trigger the post-treatment phase. At treatment cessation, remaining drug tablets will be collected be the trial pharmacy and counted to monitor compliance.

#### Venous thrombosis prophylaxis

All hospitalized patients will undergo mandatory standard neurosurgical venous thrombosis prophylaxis consisting of intermittent compression stockings plus 5,000 units of unfractionated heparin given subcutaneously twice daily (or thrice if the patient weighs over 100 kg), to be discontinued 12 h preoperatively and resumed 12 h after removal of all postoperative subdural drains. Prophylaxis will be discontinued at hospital discharge.

#### Other treatments

TXA or placebo administered as part of the TRACS trial is provided as an adjunct to the usual management of CSDH. There is no need to modify or withhold any clinically indicated treatment, including surgery, antiplatelet therapy or prophylactic heparin administration.

### Adverse events

The use of the trial’s regimen was not associated with any complication in the only available study [16]. Other, higher-dose TXA regimens have been proven safe and effective for other indications [22, 23]. However, our study population will likely be older than these earlier studies and TRACS will specifically assess TXA safety in the CSDH population. As such, adverse events will be systematically monitored during hospital stay and at each follow-up. Any event thought by the treating physician to be potentially related to TXA will be reported within 48 h of detection using a standardized Adverse Event Report Form. Adverse events to be specifically assessed at each follow-up include the incidence of deep venous thrombosis, pulmonary embolism, transient ischemic attacks, stroke, pseudo-nephrolithiasis and subjective changes in vision. Any other side effects reported by patients will also be documented. Adverse Event Reports will be screened by each site’s principal investigator as soon as they are produced. An independent safety monitoring committee will review all Adverse Event Reports, and analyze the rates of TXA adverse events after follow-up data is available for the 65th subject. As part of the interim analysis, the safety monitoring committee will produce a report to be reviewed by the trial steering committee and might request a trial review by the IRB should any concern arise. Group allocation unblinding will be performed if a highly statistically significant (*p* <0.001) difference in the rate of adverse events is identified.

Adverse events will be reported using the classification of the *Medical Dictionary for Regulatory Activities*, version 19.0.

### Measures of outcome

The primary outcome is the rate of complete CSDH radiological resolution (see definition in CSDH radiological assessment section below) by 20 weeks without intervening unplanned surgical procedure. The management strategy chosen is documented at the time of randomization as a categorical variable (no surgery planned versus elective evacuation planned versus emergent (cannot wait 24 h) evacuation planned). An unplanned surgical procedure is any surgery performed in addition to the management strategy documented at randomization. Specifically:

- If the management strategy chosen by the attending physician within the first 24 hours of presentation included a surgical evacuation of the CSDH, the primary outcome is met if a control CT scan by 20 weeks demonstrates complete radiological resolution of the CSDH.
  - ○ If a second surgical procedure is required, the subject is considered to have failed CSDH treatment and the primary outcome is not met, even if the CSDH has completely resolved by 20 weeks
  - ○ If a surgical procedure is never performed, the primary outcome is met if a control CT scan by 20 weeks demonstrates complete radiological resolution of the CSDH
- If the management strategy chosen by the attending physician within the first 24 hours of presentation consisted of expectant management, the primary outcome is met if a control CT scan by 20 weeks demonstrates complete radiological resolution of the CSDH.
  - ○ If a surgical procedure is performed, the subject is considered to have failed CSDH treatment and the primary outcome is not met, even if the CSDH has completely resolved by 20 weeks

For patients presenting with bilateral hematomas, only the largest hematoma at presentation will be considered for the primary outcome analysis.

The secondary outcomes are:

- CSDH volume at 20 weeks
- Incidence of surgical evacuation procedures
- Incidence of symptomatic and asymptomatic CSDH recurrence at 3 months following complete radiological resolution
- Cognitive functions at 10 weeks following randomization and at 3 months following the end of treatment as assessed by the Mini-Mental State Examination (MMSE)
- Functional autonomy at 10 weeks following randomization and at 3 months following the end of treatment as assessed by the modified Barthel Index (mBI) and Glasgow Outcome Scale Extended (GOSE)
- Quality of life at 10 weeks following randomization and at 3 months following the end of treatment as assessed by the EuroQol five dimensions, five levels health survey (EQ-5D-5L) questionnaire
- Length of initial hospital stay
- Number of rehospitalization
- Incidence of complications

Additional variables will be collected to allow planned subgroup analyses of the primary outcome, secondary outcomes and response to TXA when stratifying patients by:

- Conservatively versus surgically managed patients
- Level of CSDH membrane contrast-enhancement on the initial CT scan
- Level of CSDH membrane vascularization on histopathological analysis
- Evidence of coagulopathy in the CSDH

The primary analysis will be performed and reported on an intention-to-treat basis. As-treated analyses and hematoma-level analyses (for patients with bilateral CSDH) will also be performed and compared to the intention-to-treat results.

#### CSDH radiological assessment

CSDH will be assessed by serial CT scanning. Volume will be measured by segmentation of the CSDH using Osirix 6.5 (Pixmeo, Geneva, Switzerland). Contrast enhancement of the membrane will be assessed on injected sequences by volume measurement and reported as the volume/volume percentage of contrast enhancement relative to the total CSDH volume. Radiological resolution of the CSDH is defined as the absence of a measurable subdural collection discernable from cerebrospinal fluid (CSF) on the 3-mm sliced axial reconstructions of a non-enhanced isotropic CT scan. Recurrence is defined as the apparition of a new CSDH after radiological resolution of a prior known CSDH in the same location. Progression is defined as a more than 10 % increase in CSDH volume.

Radiological resolution during the treatment phase will be assessed by local investigators who will review the CT scans as they are performed and trigger the post-treatment phase upon resolution or at 20 weeks. All local investigators will undergo a standardized training in CSDH radiological resolution assessment by the study radiologist prior to subject enrollment at their center. After study completion, all CT scans will be centrally reviewed by an independent, blinded radiologist who will perform the volumetric analyses and determine the official CSDH resolution and recurrence status for outcome analyses.

#### Cognitive functions assessment

Cognitive functions will be assessed using the Mini-Mental State Examination (MMSE), a validated and widely used test [24].

#### Functional autonomy assessment

Functional autonomy will be assessed using a 10-item questionnaire: the Modified Barthel Index of Activities of Daily Living (mBI) [25]. This tool has been used in more than 2500 studies and is the most commonly used functional autonomy scale in the elderly [26]. The Glasgow Outcome Scale Extended (GOSE) will also be used at the last follow-up to measure gross functional autonomy. It is the most frequent outcome measure in neurosurgical trials, although less sensitive than the mBI for highly functional individuals.

#### Quality of life assessment

Quality of life (QOL) will be assessed using the EQ-5D-5L questionnaire, a validated QOL tool and standard test for cost-effectiveness and health utility analyses [27].

#### Hematoma analysis

Patients enrolled in the trial and undergoing surgical evacuation of their CSDH provide an opportunity for further analysis of CSDH pathophysiology. Immediately after trepanation, the CSDH and its outer membrane will be sampled. Levels of glucose, proteins, osmolarity, prothrombin time (PT), activated partial thromboplastin time (aPTT), fibrinogen, D-dimers and the platelet count will be measured in the hematoma liquid. A measure of membrane vascularization will be reported as assessed by standard hematoxylin and eosin staining and immunohistochemical labeling. Membrane analysis will be performed by a single, blinded pathologist at the coordinating center. For better reproducibility, all membranes will be analyzed simultaneously once patient recruitment has stopped and the last follow-up is completed. Sampling of the CSDH will not affect the surgical procedure itself and no tissue will be banked for future use once the trial analyses have been performed.

### Data collection and management

All data will be collected by the recruiting center’s research personnel. Paper data collection forms will be used (see Additional file 5) when questioning patients and then transcribed in a secure online database with range checks for data values. At the time of analysis, all outliers in the electronic data will be double-checked on the paper questionnaires to rule out errors in data entry. All data will be conserved for 25 years after the end of the study. Data will be collected for all enrolled patients even if the intervention is discontinued. Investigators, the trial steering committee and the safety monitoring committee will have access to the final dataset. Participant-level data and statistical code will be made available upon request for external review, substudies or meta-analysis purposes.

## Analysis

The statistical analysis plan and interim analysis plan are provided as Additional file 2 (as part of the detailed protocol). After database lock, inclusion and exclusion criteria (including imaging and clinical data) for all enrolled patients will be validated by an independent reviewer. Patients randomized despite not being eligible will be excluded from the primary analysis. Missing data will be handled by means of multiple imputation analyses. Baseline characteristics of each treatment arm will be tested for similarity using Student’s *t* test or the Mann-Whitney *U* test depending on distribution normality. Tested characteristics will include age, sex, alcohol consumption, smoking status, previous history of CSDH, use of antiplatelet medication, ACE inhibitors or glucocorticoids, previous lumbar puncture or neurosurgical procedure, recent head trauma, baseline questionnaire scores, coagulation parameters and radiological parameters. Primary and secondary outcomes analyses will be performed and reported on an intention-to-treat basis. Complementary as-treated and hematoma-level analyses will also be provided. Treatment effect on primary and secondary outcomes will be tested using the appropriate statistical tests with reporting of the effect size and a measure of precision. Briefly, the primary outcome will be assessed using Fisher’s exact test. Linear mixed models will be used to test the impact of treatment arm on CSDH volume and the various questionnaire scores. GOSE score, hospitalization length and number of readmissions will be tested using Student’s *t* test or the Mann-Whitney *U* test. Other secondary outcomes will be tested using Fisher’s exact test. Statistical significance will be defined as *p* <0.05. Risk factors for primary outcome failure will be explored using logistic regression analyses. Potential risk factors to be tested include age, alcohol consumption, smoking, previous history of CSDH, use of antiplatelet medication, ACE inhibitors or glucocorticoids, previous neurosurgical procedure, chronic renal failure, brain atrophy, convexity CSDH, loculated CSDH, enhancing CSDH membranes, high membrane vascularization and low fibrinogen level in the CSDH fluid. Subgroup analyses of the primary outcome will also be performed for conservatively managed patients and surgically managed patients.

Results will be published in peer-reviewed journals and reported in the ClinicalTrial.gov result database.

## Administrative data

### Protocol development

The protocol was developed by and following the input of:

- Dr David Mathieu MD, Department of Surgery (Neurosurgery), principal investigator
- Dr Christian Iorio-Morin MD PhD, Department of Surgery (Neurosurgery)
- Dr Jocelyn Blanchard MD, Department of Surgery (Neurosurgery)
- Dr Jean-François Castilloux MD, Department of Medicine (Hematology)
- Dr Jean Chénard MD, Department of Diagnostic Radiology
- Dr Ana-Maria Crous Tsanaclis MD, Department of Pathology
- Dr Maxime Richer MD PhD, Department of Pathology
- Dr Marie-Pierre Garant PhD, statistician

No patient was involved in the development of the study protocol.

### Trial steering committee

The trial steering committee will oversee the progress of the trial, review the safety data, review the Adverse Events Reports as they become available and provide advice regarding trial extension and funding after the power calculations are performed using the interim analysis data. The trial steering committee will consist of:

- Dr David Mathieu MD, Department of Surgery (Neurosurgery), principal investigator
- Dr Christian Iorio-Morin MD PhD, Department of Surgery (Neurosurgery)
- Dr Jocelyn Blanchard MD, Department of Surgery (Neurosurgery)

### Safety monitoring committee

The safety monitoring committee will analyze the safety data and all Adverse Events Reports available at the interim analysis. They will provide advice to the trial steering committee for protocol improvement and might request IRB reevaluation should significant safety concerns arise from the interim analysis report. The safety monitoring committee will consist of:

- Dr François Lamontagne MD MSc, Department of Medicine (Critical Care)
- Dr Jean-François Castilloux MD, Department of Medicine (Hematology)
- Dr Nicolas Dea MD MSc, Department of Surgery (Neurosurgery)
- Dr David Fortin MD MSc, Department of Surgery (Neurosurgery)

### Financial support

Funding for the TRACS trial has been provided by:

- Fond de recherche du Québec – Santé
- Centre de recherche du CHUS
- Department of Surgery, University of Sherbrooke
- Fondation Neuro-Trauma Marie-Robert

Additional funding sources are being solicited. There is neither funding nor support provided by the pharmaceutical industry.

This trial will not generate any intellectual property for the sponsor or any participating party. The funders did not participate at any point in the design of the study and will have no role in data collection, management, analysis, interpretation and reporting.

## Discussion

CSDH is a frequent and morbid condition. A better understanding of the underlying pathophysiological processes will support direct targeting of dysregulated elements of coagulation cascades that are thought to be responsible for CSDH growth. TXA is a safe and widely used inhibitor of fibrinolysis. Its effective use as a medical treatment for CSDH has been reported for 22 patients. While these results are promising, their retrospective and uncontrolled nature does not allow any conclusion regarding TXA effectiveness in CSDH to be drawn. TRACS will be the first prospective trial of TXA in CSDH.

### Safety

TRACS’ protocol requires discontinuation of TXA administration at the first CT scan demonstrating complete CSDH radiological resolution rather than at a fixed 20-week time point. This design decision will result in variable treatment duration and time of primary outcome assessment within the cohort. We anticipate that our patient population will be significantly older and at higher risk for complications than most other studies of TXA. While the low TXA dose used in TRACS will hopefully increase the safety of the intervention, the risk of thromboembolic events will likely be proportional to treatment duration. Therefore, because the goal of TXA treatment is resolution of the CSDH, we believe this variable treatment duration paradigm will maximize the safety of the intervention by allowing treatment to be discontinued in patients who have responded and for whom no additional benefit is expected, while allowing a longer course of treatment to non-responders.

Consequently, total follow-up will also be variable since the last follow-up, scheduled 3 months following treatment cessation, will depend on the time at which CSDH resolution occurred. Because the goal of this post-treatment assessment is to investigate recurrence relative to the demonstration of complete resolution and treatment cessation (as opposed to initial CSDH diagnosis and treatment start), we do not believe this design will introduce any significant bias.

### Expected impact

The TRACS trial will provide safety and incidence data, as well as effect size estimation to inform the design of a larger, definitive trial. Depending on the resulting power calculations, TRACS itself could be sufficiently powered to provide reliable evidence as to whether TXA can increase the rate of CSDH resolution following conservative management, lower the number of required surgical procedures and decrease the rate of CSDH recurrence following surgical evacuation. Exploratory analyses will investigate the impact of CSDH presence and resolution on QOL and cognitive function. While patients with no pain, motor deficit or marked cognitive impairment are often considered “asymptomatic,” these analyses might identify subtle yet clinically meaningful deficits that might benefit from a minimally-invasive treatment. Planned subgroup analyses will try to identify risk factors for CSDH recurrence and TXA failure with particular focus on prior use of antiplatelet medication, poorly-vascularized CSDH membranes and low fibrinogen levels within the CSDH fluid. CSDH sampling in the surgical patients and subsequent comparison of biochemical and immunohistological data to clinical outcome will further enhance our understanding of the pathophysiology of CSDH with the goal of predicting TXA response in individual patients. If the trial hypothesis is confirmed, it could help position TXA as a new non-invasive treatment modality for CSDH.

## Trial status

Patient enrollment began at the coordinating center in late October 2015. At the time of submission (March 2016), nine patients had been enrolled. Enrollment at the second site has not yet begun. Any change to the trial protocol will be communicated to relevant parties by the principal investigator and mentioned in the results manuscript.

## Consent to publish

Consent to publish anonymized, individual patient data will be obtained as part of the general consent for study participation.

## Additional files

Additional file 1: SPIRIT checklist. (DOC 122 kb) Additional file 2: Protocol (original French version). (PDF 3571 kb) Additional file 3: Consent form (original French version). (PDF 253 kb) Additional file 4: Consent form (English translation). (PDF 324 kb) Additional file 5: Data collection forms (original French version). (PDF 334 kb)

## Abbreviations

ACE: angiotensin-converting enzyme
CSDH: chronic subdural hematoma
CSF: cerebrospinal fluid
GOSE: Glasgow Outcome Scale Extended
HU: Hounsfield unit
ICMJE: International Committee of Medical Journal Editors
IRB: Institutional Review Board
IV: intravenous
mBI: modified Barthel Index of activities of daily living
MMSE: Mini-Mental State Examination
PAF: platelet-activating factor
PO: per os
QOL: quality of life
TRACS: Tranexamic Acid in Chronic Subdural Hematomas
TXA: tranexamic acid
