# Supplementary material for: Tranexamic Acid in Chronic Subdural Hematomas (TRACS): study protocol for a randomized controlled trial
Source: Trials. 2016 May 5;17:235. doi: 10.1186/s13063-016-1358-5 (PMC4857422; doi:10.1186/s13063-016-1358-5)
Supplement: Additional file 2: — Protocol (original French version). (PDF 3571 kb) [file 13063_2016_1358_MOESM2_ESM.pdf]

Version 7
27 avril 2016

# Protocole de recherche

## L'acide tranexamique comme traitement médical des hématomes sous-duraux chroniques

Christian Iorio-Morin, M.D., Ph.D
Résident 3, Service de neurochirurgie, Département de chirurgie
Centre hospitalier universitaire de Sherbrooke

Jocelyn Blanchard, M.D.
Service de neurochirurgie, Département de chirurgie
Centre hospitalier universitaire de Sherbrooke

David Mathieu, M.D.
Service de neurochirurgie, Département de chirurgie
Centre hospitalier universitaire de Sherbrooke
Centre de recherche du CHUS

Déposé au Comité d'éthique de la recherche en santé chez l'humain du CHUS

Centre de recherche du Centre hospitalier universitaire de Sherbrooke
3001 12e avenue Nord
Sherbrooke, Québec
J1H 5N4

# L'acide tranexamique comme traitement médical des hématomes sous-duraux chroniques

## Équipe de recherche

**Dr Christian Iorio-Morin**, M.D., Ph.D., R3, Service de neurochirurgie, CHUS
Auteur principal de l'étude.
Responsable de la planification, de la coordination, de l'exécution, de l'analyse et de la publication des résultats de l'étude. Remplacera l'infirmière de recherche en cas d'absence. Participe au repérage des patients et au prélèvement per-opératoire de spécimens.

**Dr Jocelyn Blanchard**, M.D., Service de neurochirurgie, CHUS
Co-chercheur principal.
Supervision globale du projet. Participe au repérage des patients et au prélèvement per-opératoire de spécimens.

**Dr David Mathieu**, M.D., Membre du CRCHUS, Directeur du service de neurochirurgie, CHUS
Co-chercheur principal.
Responsable de la gestion des fonds de recherche et de la supervision globale du projet. Participe au repérage des patients et au prélèvement per-opératoire de spécimens.

## Collaborateurs au repérage et au prélèvement per-opératoire de spécimens

**Dr Charles Touchette**, M.D., R1, Service de neurochirurgie, CHUS

**Dr Jonathan Picard**, M.D., R1, Service de neurochirurgie, CHUS

**Dr France Héroux**, M.D., R4, Service de neurochirurgie, CHUS

**Dr Cyril Barthélemy-Ducharme**, M.D., R5, Service de neurochirurgie, CHUS

**Dr Annie Drapeau**, M.D., R7, Service de neurochirurgie, CHUS

**Dr Khaled Effendi**, M.D., Service de neurochirurgie, CHUS

**Dr Mario Séguin**, M.D., Service de neurochirurgie, CHUS

**Dr David Fortin**, M.D., M.Sc., Service de neurochirurgie, CHUS

**Dr Nicolas Dea**, M.D., Service de neurochirurgie, CHUS

## Problématique

L'hématome sous-dural chronique est une raison de consultation fréquente en neurochirurgie et une cause importante de morbidité. Au CHUS, entre 2009 et 2012, 529 patients furent hospitalisés avec un diagnostic d'hématome sous-dural (traumatique ou non) et 154 procédures de draînage au bloc opératoire furent réalisées (1,2).

L'hématome sous-dural résulte d'un saignement intracrânien dans l'espace sous-dural localisé entre le cerveau et la dure-mère, une enveloppe fibreuse l'entourant (c.f. Figure 1). Cet espace est traversé par des veines pont qui, lors de traumatismes crâniens ou de procédures neurochirurgicales, peuvent rompre et saigner de façon plus ou moins abondante. Dans les premières heures suivant l'initiation du saignement, la cascade de coagulation s'active et entraîne la formation d'un caillot solide et adhérant aux surfaces adjacentes. Cette réaction permet à l'organisme de contrôler le saignement, colmater la brèche et réparer la veine. Dans la majorité des contextes traumatiques, ce caillot est rapidement résorbé en totalité.

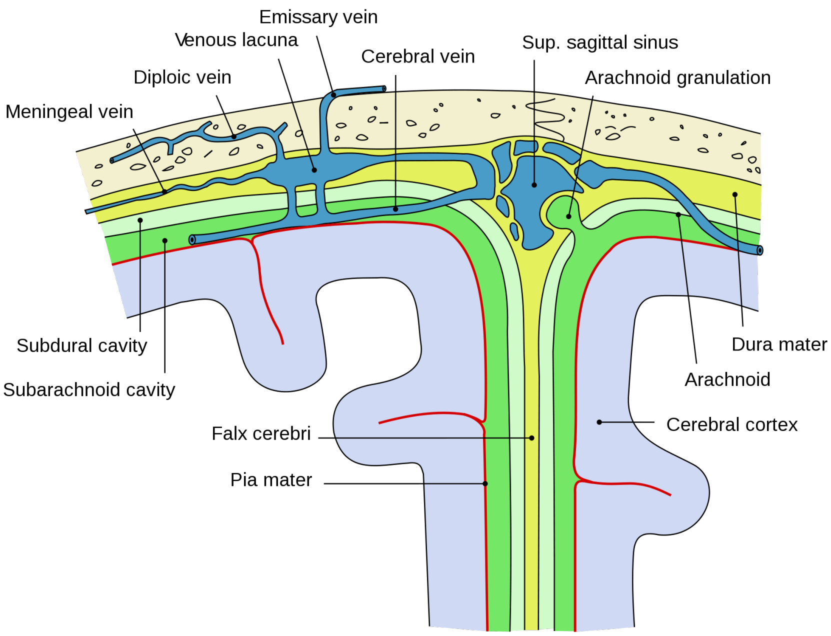


**Figure 1 : Méninges et espaces intra-crâniens (*Source : Wikipédia*)**

Pour des raisons encore incomprises, chez certains patients, notamment les patients âgés, la résorption du caillot échoue et une chronicisation de l'hématome s'installe. Chez ces patients, une membrane très vascularisée se développe lentement aux pourtours du caillot et l'isole des tissus avoisinants. Une cascade de fibrinolyse s'installe au sein du caillot et mène à sa liquéfaction. Il est postulé que ce processus, ayant lieu dans un environnement local maintenant déplété en facteurs de coagulation fonctionnels, pourrait entraîner un saignement à bas bruit de la membrane de l'hématome qui entretiendrait la collection sous-durale, maintenant dite « chronique » (3).

Si un traumatisme important est requis pour générer un hématome sous-dural chez un patient jeune sans co-morbidités, un impact mineur peut être suffisant pour déclencher un saignement

significatif chez la personne âgée et plusieurs patients âgés se présentent avec des hématomes sous-duraux chronique sans histoire de traumatisme identifiable. Une fois installé, seulement de 2,4 à 18% des hématomes sous-duraux chroniques se résolvent spontanément (4). Au contraire, la plupart des hématomes augmentent en volume et engendrent des symptômes progressifs résultant de la compression du cerveau par la collection. Les symptômes les plus fréquemment identifiés incluent les céphalées réfractaires, une diminution des fonctions cognitives incluant la mémoire et la concentration, de même que des symptômes sensitivo-moteurs, tels une faiblesse d'un membre, une difficulté à la marche ou des chutes à répétition (5). Chez certains patients, notamment ceux chez qui un saignement aigu important survient à l'intérieur de l'hématome chronique, une augmentation de la pression intracrânienne peut même mener au coma et menacer la vie.

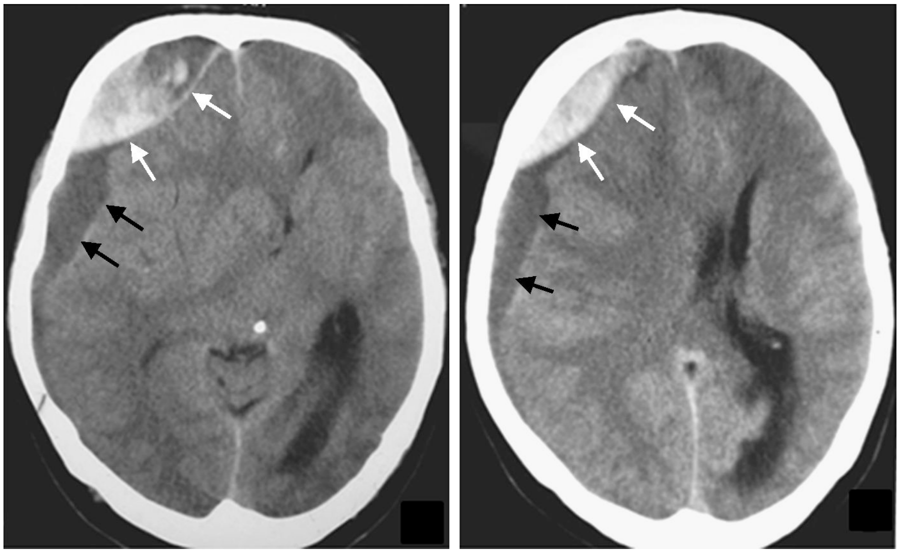

**Figure 2 : Hématome sous-dural chronique**

Le traitement usuel de l'hématome sous-dural chronique consiste en une évacuation chirurgicale de l'hématome liquide par trépanation, mais peut occasionnellement nécessiter une craniotomie. Au CHUS, cette procédure s'effectue au bloc opératoire sous anesthésie générale et nécessite une hospitalisation d'environ 6 jours en moyenne (1). En post-opératoire immédiat, le patient est généralement admis à l'unité des soins intensifs chirurgicaux ou des soins intermédiaires neurologiques où débute sa convalescence. À leur sortie de l'hôpital, les patients peuvent parfois retourner à domicile directement, mais plusieurs nécessitent un passage par une ressource intermédiaire (centre de réadaptation, maison de convalescence, etc.) avant le retour à domicile afin de contrer le déconditionnement inhérent à la période péri-opératoire. Une fois l'épisode résolu, le taux de récidive des hématomes sous-duraux après une chirurgie de draînage est de 5 à 30% (6) : il n'est donc pas rare de devoir opérer des patients à plusieurs reprises. La gravité d'un diagnostic d'hématome sous-dural ne doit pas être sous-estimée : au CHUS, entre 2009 et 2012, la mortalité associée aux hématomes sous-duraux non-traumatique et leurs complications fut de 19% (1).

La décision de procéder à une intervention chirurgicale est individualisée à chaque patient et dépend de plusieurs facteurs, dont la sévérité des symptômes, la présence de conséquences fonctionnelles et l'existence de comorbidités (7). La majorité des patients sont suivis pendant une certaine période avant que la décision d'opérer ne soit prise. Cette période d'observation

offre donc une opportunité d'intervention où une prise en charge médicale ciblant la pathophysiologie de l'hématome sous-dural chronique pourrait potentiellement améliorer la symptomatologie et repousser, voir éliminer la nécessité d'une intervention chirurgicale. Cette opportunité demeure inexploitée à l'heure actuelle.

## Recension des écrits

Aucun traitement médical n'est actuellement reconnu efficace pour résoudre les hématomes sous-duraux, diminuer les symptômes associés ou diminuer la nécessité de chirurgies de drainage. Des évidences sont toutefois disponibles concernant l'utilité de trois classes de médicaments chez les patients atteints d'hématomes sous-duraux chroniques : les corticostéroïdes, les inhibiteurs de l'enzyme de conversion de l'angiotensine et l'acide tranexamique (7).

L'administration de corticostéroïde constitue le seul mode d'intervention étudié directement. Un groupe de l'université McMaster a effectué en 2013 une méta-analyse de 250 études recensant 34 829 patients atteints d'hématomes sous-duraux chroniques (8). Cinq études s'intéressaient à l'usage de corticostéroïdes comme plan thérapeutique principal, alors que 17 incluaient les stéroïdes comme adjuvant à un traitement principal chirurgical. Ensemble, ces études démontrent une absence de bénéfice et une morbidité accrue lorsque des corticostéroïdes sont administrés (8).

En 2007, une analyse rétrospective de 438 patients opérés pour des hématomes sous-duraux chroniques a démontré que la prise concomitante d'inhibiteurs de l'enzyme de conversion de l'angiotensine comme traitement de l'hypertension artérielle semblait diminuer le risque de récidive, probablement via un mécanisme anti-angiogénique (9). Une étude prospective est actuellement en cours à cet effet (NCT00915928).

L'acide tranexamique (Cyklokapron®) est un médicament anti-fibrinolytique utilisé afin de diminuer les risques de saignement dans un contexte de polytraumatisme (10) ou de chirurgies à haut risque de saignement (cardiaque, orthopédique, urologique, gynécologique, etc.) (11), de même que pour le traitement des ménorragies réfractaires (12). Puisqu'une activation de la cascade de fibrinolyse semble être un élément pathophysiologique central menant à la chronicisation des hématomes sous-duraux, son utilisation comme traitement médical apparait logique. Deux articles font état de son utilisation dans les hématomes sous-duraux. Le premier rapport fut publié en 2000 et présente le cas d'un patient hémodialysé ayant développé des hématomes sous-duraux multi-récidivants et réfractaires à trois drainages chirurgicaux (13). Un traitement à l'acide tranexamique (20 mg/kg/48h IV pendant 4 semaines, puis 10 mg/kg/48h PO pendant 4 semaines) fut instauré et mena à une résolution complète des hématomes sans récidive à 12 mois post-évènement. Le deuxième rapport fut publié en 2013 par un groupe japonais et présente une série de 21 patients traités de façon consécutive avec 750 mg PO die d'acide tranexamique et suivis aux 21 jours avec une tomodensitométrie axiale (14). Les auteurs font état d'une résolution complète des hématomes dans 100% des cas.

Concernant l'acide tranexamique, ces deux rapports anecdotiques suggèrent un rôle potentiel dans le traitement médical de l'hématome sous-dural, mais ne permettent pas d'évaluer l'efficacité de cette intervention. Des études supplémentaires sont donc nécessaires avant d'introduire cette option thérapeutique à la prise en charge régulière des hématomes sous-duraux. En date du 14 novembre 2014, aucune étude prospective sur ce sujet n'était enregistrée dans les banques approuvées par l'ICMJE (« clinicaltrials.gov », « ISRCTN.org », « anzctr.org.au », « umin.ac.jp/ctr/index/htm », « trialregister.nl », « eudract.ema.europa.eu/ »), dans le « WHO International Clinical Trials Registry Platform », ou identifiable sur PubMed.

## Objectif

L'objectif de cette étude est de déterminer, chez les patients diagnostiqués avec un hématome sous-dural ayant une composante chronique, si l'administration d'acide tranexamique permet d'améliorer le taux de succès des traitements conventionnels (absence de récidive chez les patients opérés et résolution spontanée chez les patients non-opérés). Plus généralement, l'acide tranexamique permet-il d'augmenter le taux de résolution complète, de réduire la nécessité d'interventions chirurgicales ou de diminuer la symptomatologie?

## Devis de recherche et issues

Nous proposons de réaliser une étude clinique multicentrique, randomisée, contrôlée, à double insu, comparant un groupe traité à l'acide tranexamique à un groupe traité avec un placebo.

L'issue primaire de l'étude sera le taux de succès à 20 semaines du traitement initialement choisi au moment de la consultation :

- Si le plan de traitement était de procéder à un drainage chirurgical, l'issue primaire est atteinte si le suivi radiologique démontre une résolution complète de l'hématome dans les 20 semaines suivant le recrutement. Une deuxième chirurgie (pour récidive ou échec à la chirurgie initiale) constitue un échec au plan de traitement et ne permet pas d'atteindre l'issue primaire, même si une résolution radiologique complète est obtenue.
- Si le plan de traitement était conservateur, l'issue primaire est atteinte si le suivi radiologique démontre une résolution complète de l'hématome dans les 20 semaines suivant le recrutement. La nécessité d'un drainage chirurgical est considérée comme un échec au traitement conservateur et ne permet pas d'atteindre l'issue primaire.

Les issues secondaires seront :

- Volume de l'hématome à 20 semaines;
- Incidence de drainage chirurgical;
- Incidence de récidive pendant la durée du suivi (jusqu'à 3 mois suivant l'arrêt de l'intervention);
- Fonctions cognitives à 10 semaines et à 3 mois suivant l'arrêt de l'intervention;
- Autonomie fonctionnelle à 10 semaines et à 3 mois suivant l'arrêt de l'intervention;
- Qualité de vie à 10 semaines et à 3 mois suivant l'arrêt de l'intervention;
- Durée de l'hospitalisation pendant laquelle le diagnostic initial fut établi;
- Nombre de réhospitalisation pendant la durée du suivi;
- Survenue d'effets indésirables (thrombophlébite profonde, embolie pulmonaire, ICT, AVC, pseudonéphrolithiases, changement de la vision ou tout autre événement indésirable potentiellement causé par la prise d'acide tranexamique).

## Population à l'étude et échantillon

La population étudiée est constituée de femmes et d'hommes majeurs souffrant d'un hématome sous-dural confirmé par tomodensitométrie axiale et ayant une composante chronique. L'échantillon sera composé de patients vus consécutivement en consultation au service de neurochirurgie du Centre hospitalier universitaire de Sherbrooke ou du Centre hospitalier affilié universitaire de Québec avec un diagnostic d'hématome sous-dural ayant une composante chronique. De tous les patients référés, nous estimons qu'environ 50% rencontreront les critères d'inclusion et d'exclusion.

## Échantillonnage

L'étude utilisera un échantillonnage consécutif des patients vus en consultation au service de neurochirurgie des centres participants avec un diagnostic d'hématome sous-dural ayant une composante chronique.

## Taille de l'échantillon

Au CHUS, entre 2009 et 2012, environ 30% des hématomes sous-duraux chroniques furent drainés chirurgicalement. L'incidence rapportée de résolution des hématomes post-drainage est de 70 à 95%(6). Par ailleurs, l'incidence rapportée de résolution spontanée d'un hématome sous-dural chronique traité de façon conservatrice (sans chirurgie) varie entre 3 et 18%(4). En supposant que le taux de drainage chirurgical pour les sujets de l'étude se maintient à 30%, l'incidence estimée de l'issue primaire (résolution complète de l'hématome) se situe entre 23% et 41%. En supposant que l'administration d'acide tranexamique double le taux de résolution complète, l'échantillon nécessaire pour obtenir une puissance de 80% avec un p de 0,05 variera entre 21 (si l'incidence est de 41%) et 65 (si l'incidence est de 23%) sujets par groupe. L'objectif de recrutement sera donc de 65 sujets par groupe.

Ces calculs demeurent toutefois hautement hypothétiques. D'une part, l'incidence réelle de l'issue primaire est inconnue dans notre population et est estimée à partir d'études dont les populations sont hautement variables. Cette incidence dépendra fortement de la proportion des patients subissant un draînage chirurgical par rapport à ceux traités de façon conservatrice – un ratio qui pourrait différer des données administratives et varier en fonction des habitudes locales du centre recruteur. Par ailleurs, les deux rapports publiés jusqu'à présent suggèrent que l'acide tranexamique mène à une résolution complète des hématomes sous-duraux chroniques dans chez 100% des patients. Si tel est le cas, la taille de l'échantillon requis pour démontrer un effet du traitement sera nettement moindre. Une réponse si spectaculaire demeure toutefois peu probable à notre avis et nous avons conséquemment choisi de calculer la taille de l'échantillon avec un rapport de cote de 2. Toutefois, un effet générant un rapport de cote de 1,25 à 1,5 serait probablement cliniquement significatif vu l'innocuité relative de l'intervention, mais demanderait une taille d'échantillon substantielle (225 sujets par groupe pour un rapport de cote de 1,5 si l'incidence de l'issue primaire est de 23% dans le groupe placebo). Pour ces raisons, une analyse intérimaire des résultats sera effectuée au 65$^{e}$ sujet afin d'établir une incidence précise pour notre issue primaire et estimer l'effet du traitement. Si ce dernier s'avère significatif, l'étude sera poursuivie jusqu'au recrutement du 130$^{e}$ sujet afin d'augmenter la puissance des analyses de sous-groupe. Si le traitement n'a pas d'effet significatif au 65$^{e}$ sujet, les calculs de puissance seront révisés avec l'incidence réelle de résolution de l'issue primaire dans l'échantillon disponible afin d'ajuster la puissance de l'étude et planifier le budget en conséquence.

## Critères d'inclusion et d'exclusion

Critère d'inclusion :

- Tout sujet majeur chez qui une tomodensitométrie axiale datant de moins de 14 jours démontre l'existence d'un hématome sous-dural ayant une composante chronique.
    - L'hématome sous-dural est défini radiologiquement comme une collection liquidienne hémorragique dans l'espace sous-dural;

  - La composante chronique est définie comme une région hypodense (< 25 HU) ou isodense (25-35 HU) au sein de la collection sous-durale en l'absence d'anémie ou de coagulopathie;
  - En présence d'anémie ou de coagulopathie, des signes supplémentaires doivent suggérer l'existence d'un processus chronique sous-jacent. Par exemple :
    - Hématomes sous-duraux bilatéraux
    - Loculation de l'hématome
    - Présence d'une membrane aux pourtours de l'hématome

Critères d'exclusion de l'étude ou de cessation de la médication :

- Hématome sous-dural exclusivement aigu (car la pathophysiologie est différente de l'hématome sous-dural chronique)
- Maladie thrombotique, thrombo-embolique ou athéro-embolique **active** (car l'effet anti-fibrinolytique du cyklokapron pourrait potentiellement décompenser la pathologie) incluant, par exemple :
  - Thrombophlébite profonde dans les 6 derniers mois
  - Thrombose cérébrale dans les 6 derniers mois
  - Sténose carotidienne symptomatique non-opérée
  - Accident vasculaire cérébral nouveau dans la dernière année
  - Syndrome coronarien aiguë dans la dernière année
- Sujet avec antécédent de thrombophlébite idiopathique (car l'effet anti-fibrinolytique du cyklokapron pourrait potentiellement décompenser la pathologie)
- Sujet avec antécédent d'embolie pulmonaire idiopathique (car l'effet anti-fibrinolytique du cyklokapron pourrait potentiellement décompenser la pathologie)
- Sujet connu pour une thrombophilie héréditaire (car l'effet anti-fibrinolytique du cyklokapron pourrait potentiellement décompenser la pathologie) incluant, par exemple :
  - Facteur V de Leiden
  - Mutation du gène de l'antithrombine III
  - Déficience en protéine C
  - Déficience en protéine S
- Sujet atteint de fibrillation auriculaire (car l'effet anti-fibrinolytique du cyklokapron pourrait potentiellement aggraver l'effet d'une embole)
- Sujet porteur d'une valve cardiaque métallique (car l'effet anti-fibrinolytique du cyklokapron pourrait potentiellement aggraver l'effet d'une embole)
- Sujet porteur de *stent* vasculaire installé dans la dernière année (car l'effet anti-fibrinolytique du cyklokapron pourrait potentiellement favoriser une sténose si le *stent* n'est pas encore ré-épithélialisé)
- Sujet ayant subi une chirurgie vasculaire ou cardiaque dans les derniers six mois (car l'effet anti-fibrinolytique du cyklokapron pourrait potentiellement favoriser une thrombose au site des sutures endovasculaires) incluant, par exemple :
  - Endartérectomie
  - Pontage
  - Angioplastie
- Sujet en investigation pour une néoplasie ou souffrant d'une néoplasie active (car l'effet anti-fibrinolytique du cyklokapron pourrait potentiellement favoriser une thrombose)
- Sujet sous hormonothérapie dans le contexte d'un cancer
- Hématurie macroscopique (car l'effet anti-fibrinolytique du cyklokapron pourrait entretenir des caillots intra-rénaux et générer des pseudolithiases)
- Allergie documentée ou rapportée à l'acide tranexamique
- Femme enceinte ou en allaitement (car le cyklokapron n'est pas démontré comme sécuritaire en grossesse ou en allaitement)
- Prise concomitante d'anticoagulant

- Prise concomitante de contraceptifs oraux combinés (car l'effet anti-fibrinolytique du cyklokapron pourrait potentiellement avoir un effet pro-thrombogène)
- Sujet de moins de 18 ans
- Refus du médecin traitant

Ne sont **pas** considérés comme des critères d'exclusion :

- Hématome sous-dural asymptomatique ou découvert fortuitement
- Hématome sous-dural traumatique
- Antécédent de thrombophlébite profonde, superficielle ou d'embolie pulmonaire maintenant résolu
- Prise concomitante d'hormonothérapie de remplacement

## Recrutement des sujets

Les patients admissibles seront repérés par les membres de l'équipe de neurochirurgie au moment du diagnostic d'hématome sous-dural. Il s'agira le plus souvent du médecin résident ou du patron de garde en neurochirurgie à l'urgence, sur les étages ou en clinique externe. Le médecin informera le patient de l'existence du projet de recherche et lui offrira d'être rencontré par l'infirmière de recherche s'il est intéressé à participer. Si le patient accepte, l'infirmière de recherche rencontrera le patient dans les deux heures au lieu de la consultation (étage, urgence, etc.). L'infirmière présentera le projet de recherche, vérifiera l'intérêt du patient pour le projet, confirmera son éligibilité et obtiendra le consentement écrit des patients intéressés. Une copie papier du formulaire de consentement sera remise au sujet et la copie signée sera déposée au dossier de projet du sujet. Le sujet est libre de se retirer du projet à tout moment.

Advenant le cas où la consultation aurait lieu à plus de 24h d'un jour ouvrable (i.e. vendredi soir, samedi ou lors de congés fériés), le consentement écrit sera obtenu directement par le résident de garde, qui avisera l'infirmière de recherche sur sa boîte vocale (c.f. Annexe 3). Cette dernière rencontrera le patient et confirmera le consentement lors du prochain jour ouvrable. Cette démarche vise à permettre un recrutement optimal sans délais dans l'initiation du traitement, tout en maintenant la qualité et la standardisation des consentements. Le patient est libre de se retirer du projet à tout moment.

Advenant le cas où le patient serait inapte à consentir à ses soins, le consentement au projet sera obtenu auprès du proche le plus significatif (p. ex. conjoint, enfant, ami). Ce dernier peut prendre le temps de consulter d'autres membres de la famille au besoin, sachant que le protocole ne sera pas initié avant l'obtention formelle du consentement. Dès le retour de l'aptitude à consentir du sujet, ce dernier sera rencontré par l'infirmière de recherche, le projet lui sera présenté et un nouveau formulaire de consentement devra être signé sans quoi le sujet sera retiré du projet. Si le proche le plus significatif se voit dans l'impossibilité de se déplacer pour signer le consentement, un consentement téléphonique sera jugé acceptable.

## Variables et collecte des données

Les variables mesurées sont présentées dans le tableau suivant. À noter qu'afin de permettre une certaine latitude en terme d'analyses *post-hoc*, certaines variables qui seront analysées de façon binaire ou catégorique (voir section Analyse) sont collectées de façon catégorique ou continue.

| Variable | Type | Instrument de mesure | Notes |
|---|---|---|---|
| Nature du médicament administré | Binaire :<br>• Placébo<br>• Acide tranexamique | Variable indépendante assignée par randomisation | Étude à double insu : cette variable est donc inconnue du personnel soignant et des chercheurs tant pendant l'étude que pendant l'analyse. |
| **Données démographique et caractéristiques de base** | | | |
| Âge au recrutement | Continue (années) | Révision du dossier clinique | |
| Sexe | Binaire :<br>• Femme<br>• Homme | Révision du dossier clinique | |
| Dominance du patient | Catégorique :<br>• Droitier<br>• Gaucher<br>• Ambidextre | Questionnaire initial | |
| Consommation d'alcool | Continue (consommation par semaine) | Questionnaire initial | |
| Tabagisme actif | Binaire :<br>• Non<br>• Oui | Questionnaire initial | Pour être considéré comme non-actif, le sujet doit avoir cessé de fumer depuis plus de 4 semaines. |
| Tabagisme cumulatif | Continue (paquets-années) | Questionnaire initial | |
| Antécédent d'hématome sous-dural | Catégorique :<br>• Non<br>• Oui, non drainé<br>• Oui, drainé | Questionnaire initial | |
| Prise récente (moins de 1 mois) d'un médicament antiplaquettaire | Catégorique :<br>• Non<br>• Oui, unique<br>• Oui, plusieurs | Questionnaire initial | Exemples de médicaments antiplaquettaires : Acide acétylsalicylique, Clopidogrel. |
| Prise récente (moins de 1 mois) d'un médicament anticoagulant | Binaire :<br>• Non<br>• Oui | Questionnaire initial | Exemples de médicaments anticoagulants : Héparine à dose thérapeutique, Warfarin, Dabigatran, Rivaroxaban, Apixaban. |
| Prise récente (moins de 1 mois) d'IECA ou d'ARA | Catégorique :<br>• Non<br>• Oui, IECA<br>• Oui, ARA<br>• Oui, les deux | Questionnaire initial | |
| Prise récente (moins de 1 mois) de corticostéroïde | Binaire :<br>• Non<br>• Oui | Questionnaire initial | |
| Convulsion (dans la | Continue (nombre) | Questionnaire initial | |

| | | | |
|---|---|---|---|
| dernière année) | | | |
| Dérivation de LCR (dans la dernière année) | Catégorique :<br>• Non<br>• Oui, ponction lombaire<br>• Oui, dérivation ventriculo-péritonéale<br>• Oui, autre | Questionnaire initial | |
| Antécédent de procédure neurochirurgicale récente (< 1 mois avant le début des symptômes) | Catégorique :<br>• Non<br>• Oui, procédure extra-crânienne<br>• Oui, procédure intra-crânienne | Questionnaire initial | Exemples de procédures extra-crâniennes : révision de shunt, drain lombaire, ponction lombaire, chirurgie spinale intra- ou extra-durale.<br><br>Exemples de procédures intra-crâniennes : craniotomie, trépanation, dérivation ventriculaire externe. |
| Histoire de traumatisme récent (< 1 mois avant le début des symptômes) | Catégorique :<br>• Non<br>• Oui, mineur<br>• Oui, majeur | Questionnaire initial | Un traumatisme majeur implique au minimum un diagnostic de TCC léger (Glasgow < 15, perte de conscience, amnésie, etc.) |
| Tout facteur jugé pertinent | Texte libre | Questionnaire initial | |
| **Présentation clinique** | | | |
| Contexte de la consultation | Catégorique :<br>• Consultation élective en clinique externe<br>• Consultation non-urgente à l'urgence ou sur un étage<br>• Consultation urgente | Questionnaire initial | Une consultation urgente est une consultation nécessitant un avis neurochirurgical immédiat, par opposition à une consultation non-urgente à l'urgence ou sur un étage où l'avis neurochirurgical est donné le jour même par soucis d'efficacité, mais où un avis en externe aurait été acceptable. |
| Symptôme principal à la présentation | Catégorique :<br>• Asymptomatique<br>• Céphalées<br>• Confusion<br>• Somnolence<br>• Chute ou trouble d'équilibre<br>• Faiblesse d'un membre<br>• Trouble du langage<br>• Trouble de mémoire<br>• Vomissements<br>• Trouble visuel<br>• Convulsion<br>• Autre | Questionnaire initial | |
| Date du début approximatif des symptômes | Date | Questionnaire initial | Date des premiers symptômes en lien avec le diagnostic. Il ne s'agit pas nécessairement du symptôme principal ayant mené à la consultation. |

| | | | |
|---|---|---|---|
| Fonctions cognitives au recrutement | Continue | Questionnaire MMSE | |
| Qualité de vie au recrutement | Catégorique | Questionnaire EQ-5D-5L | |
| Qualité de vie subjective au recrutement | Continue | Questionnaire EQ-5D-5L VAS | |
| Autonomie fonctionnelle au recrutement | Continue | Questionnaire mBI | |
| Plan initial de traitement | Catégorique :<br>• Conservateur<br>• Drainage électif<br>• Drainage urgent | Questionnaire initial | Plan initial de traitement sur lequel le patient et l'équipe médicale s'entendent au moment de la consultation. Un drainage urgent est un drainage devant être réalisé < 24 h. Un drainage électif est un drainage pour lequel le délai n'a pas d'importance. |
| **Bilan biochimique initial** | | | |
| Hémoglobine au diagnostic | Continue | Révision du dossier clinique | |
| Décompte plaquettaire au diagnostic | Continue | Révision du dossier clinique | |
| PT au diagnostic | Continue | Révision du dossier clinique | Prendre la valeur avant le renversement de l'anticoagulothérapie si le patient fut renversé d'urgence |
| PTT au diagnostic | Continue | Révision du dossier clinique | Prendre la valeur avant le renversement de l'anticoagulothérapie si le patient fut renversé d'urgence |
| Clairance de créatinine au diagnostic | Continue | Révision du dossier clinique | |
| **Tomodensitométrie initiale** | | | |
| Date de la tomodensitométrie diagnostique | Date | Révision du dossier clinique | |
| Atrophie cérébrale | Continue | Tomodensitométrie axiale du diagnostic | L'atrophie cérébrale sera estimée par le ratio bicaudé (distance axiale entre la tête des deux noyaux caudés divisé par le diamètre interne du crâne au même endroit) sur la tomodensitométrie |
| Localisation de l'hématome sous-dural initial au diagnostic | Catégorique :<br>• Convexité frontale<br>• Convexité fronto-pariétale<br>• Base frontale<br>• Base fronto-temporale<br>• Interhémisphérique | Tomodensitométrie axiale du diagnostic | La localisation est définie selon la classification de Nakaguchi *et al.* (15) |

| | | | |
|---|---|---|---|
| Côté de l'hématome sous-dural initial au diagnostic | Catégorique :<br>• Droit<br>• Gauche<br>• Bilatéral | Tomodensitométrie axiale du diagnostic | |
| Volume de l'hématome sous-dural initial au diagnostic | Continue (mL) | Tomodensitométrie axiale du diagnostic | Le volume sera mesuré par segmentation de l'hématome sur la tomodensitométrie non-injectée |
| Composition de l'hématome sous-dural initial | Catégorique :<br>• Homogène<br>• Laminaire<br>• Séparé<br>• Trabeculaire | Tomodensitométrie axiale du diagnostic | La composition est définie selon la classification de Nakaguchi *et al.* (15) |
| Volume rehaussant dans l'hématome initial | Continue (mL) | Tomodensitométrie axiale injectée | |
| **Suivi radiologique** | | | |
| Volume de l'hématome sous-dural initial à 5, 10, 15 et 20 semaines du recrutement, puis à 3 mois de l'arrêt du médicament | Continue (mL) | Tomodensitométrie axiale | Une résolution démontrée à 10 semaines implique automatiquement une résolution de l'hématome **initial** à 15 et 20 semaines : des tomodensitométries subséquentes ne sont donc pas nécessaires et le volume sera obligatoirement de 0 mL. |
| Composition de l'hématome sous-dural à 5, 10, 15 et 20 semaines du recrutement, puis à 3 mois de l'arrêt du médicament | Catégorique :<br>• Aigu sur chronique<br>• Purement chronique | Tomodensitométrie axiale | |
| Apparition d'un nouvel hématome sous-dural pendant le suivi | Catégorique :<br>• Non<br>• Oui, récidive au site initial<br>• Oui, nouvel hématome à distance | Tomodensitométrie axiale | |
| **Suivi clinique** | | | |
| Résolution du symptôme initial | Catégorique :<br>• Non<br>• Partiellement<br>• Complètement | Questionnaire de suivi | |
| Fonctions cognitives à 10 semaines et à 3 mois de l'arrêt du médicament | Continue | Questionnaire MMSE | |
| Qualité de vie à 10 semaines et à 3 mois de l'arrêt du médicament | Catégorique et Continue | Questionnaire EQ-5D-5L et EQ-5D-5L VAS | |

| | | | |
|---|---|---|---|
| Autonomie fonctionnelle à 10 semaines et à 3 mois de l'arrêt du médicament | Continue | Questionnaire mBI | |
| Durée de l'hospitalisation pendant laquelle le diagnostic initial fut établis | Continue (jours) | Révision du dossier clinique | |
| Nombre de ré-hospitalisation pour hématome sous-dural pendant la durée du suivi | Continue | Révision du dossier clinique | |
| Nombre de ré-hospitalisation pour toute cause pendant la durée du suivi | Continue | Révision du dossier clinique | |
| **Bilan per-opératoire (si un drainage chirurgical est effectué)** | | | |
| Drainage chirurgical de l'hématome initial | Binaire :<br>• Non<br>• Oui | Révision du dossier clinique | |
| Contexte de la chirurgie | Catégorique :<br>• Chirurgie élective<br>• Chirurgie d'urgence | Formulaire de protocole opératoire | Une chirurgie d'urgence est une chirurgie qui n'aurait pas pu attendre au lendemain |
| Date de la chirurgie | Date | Formulaire de protocole opératoire | |
| Type de chirurgie | Catégorique :<br>• Trépanation simple<br>• Trépanation double<br>• Craniotomie<br>• Autre | Formulaire de protocole opératoire | |
| Lieu de la chirurgie | Catégorique :<br>• Bloc opératoire<br>• Draînage au chevet | Formulaire de protocole opératoire | |
| Installation de drain | Catégorique :<br>• Non<br>• Oui, sous-dural<br>• Oui, sous-galéal<br>• Oui, autre | Formulaire de protocole opératoire | |
| Orientation du drain | Catégorique :<br>• Aucun drain<br>• Extrémité en antérieur<br>• Extrémité en postérieur | Formulaire de protocole opératoire | |
| Irrigation intra-opératoire | Catégorique :<br>• Non | Formulaire de protocole opératoire | |

| | | | |
|---|---|---|---|
| | • Oui | | |
| Mobilisation post-opératoire | Catégorique :<br>• Repos au lit<br>• Mobilisation selon tolérance | Formulaire de protocole opératoire | |
| Nombre total de chirurgies de drainage pendant le suivi | Continue | Révision du dossier clinique | |
| Vascularisation du spécimen de membrane | Continue (vaisseaux/mm$^3$) | Révision du dossier clinique | Analyse immunohistochimique (avec le facteur VIII) du nombre moyen de vaisseaux/mm$^3$. Effectué par le laboratoire de pathologie sur le spécimen chirurgical final (aucune analyse sur les congélations). |
| Formule sanguine complète | Continue | Révision du dossier clinique | |
| Décompte cellulaire sur l'hématome | Continue | Révision du dossier clinique | |
| Glycémie | Continue | Révision du dossier clinique | |
| Glucose sur l'hématome | Continue | Révision du dossier clinique | |
| Protéines totales sériques | Continue | Révision du dossier clinique | |
| Protéines sur l'hématome | Continue | Révision du dossier clinique | |
| Osmolarité sérique | Continue | Révision du dossier clinique | |
| Osmolarité de l'hématome | Continue | Révision du dossier clinique | |
| PT sérique | Continue | Révision du dossier clinique | |
| PT de l'hématome | Continue | Révision du dossier clinique | |
| PTT sérique | Continue | Révision du dossier clinique | |
| PTT de l'hématome | Continue | Révision du dossier clinique | |
| Fibrinogène sérique | Continue | Révision du dossier clinique | |
| Fibrinogène de l'hématome | Continue | Révision du dossier clinique | |
| D-dimères sériques | Continue | Révision du dossier clinique | |
| D-dimères de l''hématome | Continue | Révision du dossier clinique | |
| **Sécurité du médicament** | | | |

| | | | |
|---|---|---|---|
| Diagnostic de thrombophlébite profonde pendant la durée du suivi | Binaire :<br>• Non<br>• Oui | Révision du dossier clinique et questionnaire de fin d'étude | |
| Diagnostic d'embolie pulmonaire pendant la durée du suivi | Binaire :<br>• Non<br>• Oui | Révision du dossier clinique et questionnaire de fin d'étude | |
| Diagnostic d'ICT pendant la durée du suivi | Binaire :<br>• Non<br>• Oui | Révision du dossier clinique et questionnaire de fin d'étude | |
| Diagnostic d'AVC pendant la durée du suivi | Binaire :<br>• Non<br>• Oui | Révision du dossier clinique et questionnaire de fin d'étude | |
| Diagnostic de pseudo-néphrolithiases pendant la durée du suivi | Binaire :<br>• Non<br>• Oui | Révision du dossier clinique et questionnaire de fin d'étude | |
| Toute complication attribuable à l'administration d'acide tranexamique | Texte libre | Révision du dossier clinique et questionnaire de fin d'étude | Cette variable sera analysée individuellement pour chaque sujet |

## Instruments de mesure

### Tomodensitométrie axiale

La tomodensitométrie axiale (CT Scan) représente l'examen de choix pour le diagnostic et le suivi des hématomes sous-duraux et est régulièrement effectuée au CHUS dans ce but. Il s'agit d'une procédure d'imagerie par rayon X permettant de générer des reconstructions tridimensionnelles du contenu de la boite crânienne, avec une excellente sensibilité pour détecter des hémorragies. L'examen nécessite que le patient se déplace au département de radiologie où est localisé l'appareil. L'examen comme tel dure environ 5 minutes pendant lesquelles le patient est allongé sur le dos. L'examen est indolore, mais les patients claustrophobes peuvent parfois ressentir de l'anxiété temporaire.

La première tomodensitométrie axiale du protocole nécessite une injection intraveineuse d'iode permettant l'identification de la membrane et la segmentation des hématomes isodenses, une technique utilisée dans plusieurs centres (16,17), mais qui n'est pas réalisée de façon systématique au CHUS. Cette étape requiert l'installation d'une voie intraveineuse pendant la durée de l'examen. L'injection ne sera pas effectuée en cas d'allergie à l'iode ou d'insuffisance rénale (DFGe < 60 mL/min).

Suite à l'examen, le sujet peut retourner immédiatement à domicile ou à l'étage où il est hospitalisé.

Le protocole de suivi radiologique a été conçu pour calquer le suivi radiologique usuel des hématomes sous-duraux au CHUS. Ainsi, bien que les tomodensitométries axiales de contrôle seront prescrites de façon standardisée par l'infirmière de recherche, elles serviront également de suivi radiologique au médecin traitant.

Le volume des hématomes sera mesuré par segmentation en utilisant le logiciel Osirix 6.5 (Pixmeo, Genève, Suisse). Le rehaussement de la membrane sera évalué sur les séquences injectées en calculant le pourcentage volume/volume de la zone rehaussante par rapport à l'hématome lui-même. La résolution radiologique sera définie comme l'absence de collection sous-durale discernable du LCR. Pendant l'étude, le médecin coordonnateur du centre sera responsable d'établir pour les patients de son centre s'il y a résolution ou non de l'hématome afin d'enclencher ou non l'arrêt de l'intervention. Une formation standardisée sera offerte aux médecins coordonnateurs afin d'uniformiser l'évaluation de la réponse radiologique. Une fois l'étude terminée, les mesures officielles servant à déterminer l'issue primaire seront effectuées par un radiologiste unique et aveugle face aux patients et leur traitement.

### Mini Mental State Examination

Le Mini-Mental State Examination (MMSE) est un test de dépistage de la démence. Il s'agit d'un questionnaire administrable en 10 minutes par un professionnel de la santé en présence du patient. Son utilisation est très répandue, ce qui en fait un étalon à partir duquel baser les autres résultats (19).

Le questionnaire complet est présenté en Annexe 4.

### EQ-5D-5L

Le questionnaire EQ-5D-5L est un questionnaire validé de qualité de vie. Son usage répandu facilitera la comparaison à d'autres études. Des valeurs standardisées sont disponibles pour comparer les résultats de notre population à une population saine de référence. Des pondérations permettent également de générer une valeur globale permettant des analyses coût-bénéfice.

Le questionnaire complet est présenté en Annexe 5

### Modified Barthel Index of Activities of Daily Living (mBI)

Le Modified Barthel Index of Activites of Daily Living (mBI) est un outil d'évaluation de l'autonomie fonctionnelle constitué de 10 questions simples portant sur les activités de la vie quotidienne (21). Utilisé dans plus de 2 500 études, il s'agit du questionnaire le plus fréquemment employé pour mesurer le niveau d'autonomie chez les sujets âgés (22). Il peut être administré en 2 à 10 minutes par une infirmière ou un membre de l'équipe de recherche.

Le questionnaire complet est présenté en Annexe 6.

### Glasgow Outcome Scale Extended (GOSE)

Le GOSE est un questionnaire standardisé en 8 points évaluant le niveau fonctionnel du sujet. Il s'agit de l'une des échelles pronostiques les plus utilisées en neurochirurgie.

Le questionnaire complet est présenté en Annexe 7.

### Analyse de vascularisation des membranes

Les spécimens de membrane des hématomes sous-duraux opérés seront collectés par les centres recruteurs et envoyés au CHUS à la fin de l'étude pour analyse. L'ensemble des spécimens sera évalué par le même pathologiste à l'aide d'une coloration à l'hématoxyline et l'éosine de même que par un marquage immunohistochimique au facteur VIII qui permettront de calculer le volume moyen de vaisseaux par $mm^3$.

## Déroulement de l'étude

### Préparatifs préalables à l'étude

- Les médicaments seront achetés sur le marché :
  - Des comprimés de placebo;
  - Des comprimés d'acide tranexamique de 750 mg.
- Les médicaments servis au patient par la pharmacie du centre recruteur.

### Identification et recrutement

- L'identification des patients potentiellement admissibles et intéressés est faite au moment de la consultation par le résident de neurochirurgie ou le neurochirurgien

traitant. Avec l'accord du patient, ce dernier avise l'infirmière de recherche responsable du projet.
- Le recrutement des sujets se fait par l'infirmière de recherche qui les rencontre dans les deux heures suivant la consultation initiale. L'infirmière leur présente le projet et obtient le consentement écrit des patients intéressés. Une copie papier du formulaire de consentement est remise au patient et la copie signée est déposée au dossier de projet du patient.

**Randomisation**

- La randomisation a lieu suite à l'obtention du consentement. L'infirmière contacte la pharmacie du centre recruteur qui assigne le sujet à un groupe (A ou B) à l'aide d'une table de randomisation par blocs de 4 sujets et stratifiée pour la prise d'antiplaquettaires et par centre recruteur.
- L'étude est réalisée à double insu. Ainsi, l'assignation du sujet au groupe A ou B sera faite à l'insu du personnel soignant et des auteurs de l'étude. La table de randomisation sera conservée à la pharmacie du centre coordonateur et ne sera dévoilée aux auteurs qu'au moment de l'analyse des résultats. Le traitement administré à chacun des groupes (acide tranexamique vs placébo) ne sera quant à lui révélé aux auteurs qu'une fois l'analyse statistique complétée et les tableaux de résultats produits.
- La pharmacie prépare par la suite 115,5 comprimés (1,5 co PO die pour 11 semaines) du médicament correspondant (acide tranexamique ou placebo)
    - Si la randomisation doit avoir lieu hors des heures ouvrables, le résident de garde utilise une trousse de réserve pré-randomisée qui sera conservée à l'étage de neurochirurgie. Cette trousse contiendra les 115,5 comprimés.
    - Si le patient est hospitalisé, les médicaments sont acheminés à l'infirmière du patient qui les administre comme « médicament du patient » selon la posologie prescrite (1,5 co PO die).
    - Si le patient obtient congé ou est traité d'emblée en externe, les comprimés sont remis directement au patient avec un feuillet expliquant la posologie (c.f. Annexe 10).
    - Le nombre de comprimés (115,5) est suffisant pour permettre au patient de recevoir le traitement expérimental jusqu'à l'obtention, par l'infirmière, du résultat de sa deuxième tomodensitométrie axiale à 10 semaines.
- L'administration du médicament débute dès la randomisation et la réception des comprimés.
    - Une fois le traitement débuté, le patient est pris en charge de façon usuelle par l'équipe traitante qui peut, par exemple, choisir d'intervenir chirurgicalement ou d'observer le sujet, indépendamment du projet de recherche. Le patient peut être hospitalisé ou congédié selon la situation.
    - La participation au projet n'entraine aucune autre modification au traitement usuel qu'un patient recevrait à l'extérieur du protocole.
    - La participation au projet ne nécessite aucun changement dans la médication usuelle du patient.
    - La participation au projet ne modifie aucunement les indications d'un drainage chirurgical ni la procédure chirurgicale elle-même.

**Collecte de données initiale et suivi per-traitement**

- Suite à l'obtention du consentement ou le premier jour ouvrable suivant l'obtention du consentement (si le patient est recruté un soir ou une fin de semaine), l'infirmière :
    - Complète la liste de validation du consentement (cf. Annexe 1)

    - Inscrit le numéro de dossier clinique, de même que les données démographiques du sujet dans la base de données en ligne sécurisée;
    - Complète le questionnaire initial (cf. Annexe 2);
    - Administre le test cognitif MMSE (cf. Annexe 4);
    - Administre le test de qualité de vie EQ-5D-5L (cf. Annexe 5);
    - Administre le test d'autonomie fonctionnelle mBI (cf. Annexe 6)
    - Confirme la présence au dossier d'un bilan biochimique de routine datant de moins de 14 jours et prescrit, au nom du médecin coordonnateur du centre recruteur, les prélèvements manquants s'il y a lieu :
        - Formule sanguine complète (pour le décompte plaquettaire);
        - Créatinine sérique (pour l'injection d'iode);
        - PT;
        - PTT;
    - Prescrit, au nom du médecin coordonnateur du centre recruteur, une tomodensitométrie axiale injectée à réaliser au plus tard 24 h suivant la première dose d'acide tranexamique, avant toute procédure chirurgicale.
    - Prescrit, au nom du médecin coordonnateur du centre recruteur, la prochaine tomodensitométrie axiale (à 5 semaines) en précisant la participation du sujet au projet de recherche;
    - Dépose au dossier clinique le formulaire de protocole opératoire à remplir par le chirurgien advenant une procédure chirurgicale.

- À 5 semaines :
    - Une tomodensitométrie axiale de contrôle est effectuée;
    - L'infirmière contacte le médecin coordonnateur du centre recruteur qui évaluera la résolution de l'hématome :
        - S'il y a résolution complète de l'hématome sous-dural, le protocole de suivi post-traitement est enclenché (voir plus bas);
        - S'il n'y a pas résolution complète de l'hématome sous-dural, l'infirmière prescrit, au nom du médecin coordonnateur du centre recruteur, une nouvelle tomodensitométrie axiale de contrôle à 10 semaines.

- À 10 semaines :
    - Le sujet est rencontré par l'infirmière qui :
        - Complète le questionnaire de suivi (cf. Annexe 8);
        - Administre le test cognitif MMSE (cf. Annexe 4);
        - Administre le test de qualité de vie EQ-5D-5L (cf. Annexe 5);
        - Administre le test d'autonomie fonctionnelle mBI (cf. Annexe 6);
    - Une tomodensitométrie axiale est effectuée (à moins d'une résolution complète à l'examen précédent);
    - L'infirmière contacte le médecin coordonnateur du centre recruteur qui évaluera la résolution de l'hématome :
        - S'il y a résolution complète de l'hématome sous-dural, le protocole de suivi post-traitement est enclenché (voir plus bas);
        - S'il n'y a pas résolution complète de l'hématome sous-dural :
            - L'infirmière prescrit, au nom du médecin coordonnateur du centre recruteur, une nouvelle tomodensitométrie axiale de contrôle à 15 semaines;
            - L'infirmière contacte la pharmacie afin d'obtenir 94,5 comprimés supplémentaires requis pour compléter le traitement à un total de 20 semaines. L'infirmière avise également le patient du lieu de collecte.

- À 15 semaines :
  - Une tomodensitométrie axiale de contrôle est effectuée (à moins d'une résolution complète à l'examen précédent);
  - L'infirmière contacte le médecin coordonnateur du centre recruteur qui évaluera la résolution de l'hématome :
    - S'il y a résolution complète de l'hématome sous-dural, le protocole de suivi post-traitement est enclenché (voir plus bas);
    - S'il n'y a pas résolution complète de l'hématome sous-dural, l'infirmière prescrit, au nom du médecin coordonnateur du centre recruteur, une nouvelle tomodensitométrie axiale de contrôle à 20 semaines.

- À 20 semaines :
  - Une tomodensitométrie axiale de contrôle est effectuée (à moins d'une résolution complète à l'examen précédent);
  - Le protocole de suivi post-traitement est enclenché (voir plus bas).

**Suivi post-traitement**

- Le suivi post-traitement est enclenché après 20 semaines de traitement ou dès qu'une tomodensitométrie axiale (prescrite par l'étude ou réalisée dans tout autre contexte) confirme une résolution complète de l'hématome sous-dural. À ce moment, l'infirmière :
  - Contacte le patient par téléphone et l'avise de cesser le traitement expérimental et de rapporter les comprimés restant au prochain suivi avec le personnel de recherche;
  - Prescrit, au nom du médecin coordonnateur du centre recruteur, la dernière tomographie axiale de contrôle 3 mois suivant l'arrêt du médicament.

- À 3 mois post-arrêt du traitement :
  - Le sujet est rencontré par l'infirmière qui :
    - Collecte les comprimés non-utilisés et les administre à la pharmacie pour décompte;
    - Complète le questionnaire de fin d'étude (cf. Annexe 8)
    - Administre le test cognitif MMSE (cf. Annexe 4);
    - Administre le test de qualité de vie EQ-5D-5L (cf. Annexe 5);
    - Administre le test d'autonomie fonctionnelle mBI (cf. Annexe 6)
    - Administre le test d'issue fonctionnelle GOSE (cf. Annexe 7);
  - Une tomodensitométrie axiale de contrôle est effectuée;
  - L'étude prend fin.

**Particularités chirurgicales**

Advenant la décision, par l'équipe médicale, de procéder à une intervention chirurgicale, un échantillon de la membrane de l'hématome sera prélevé par le chirurgien à l'aide d'une pince à biopsie et envoyé pour analyse au laboratoire de pathologie. La membrane sera analysée de façon centralisée au CHUS au moyen d'une coloration à l'hématoxyline et l'éosine, de même que par un marquage immunohistochimique au facteur VIII afin de quantifier le nombre de vaisseaux par $mm^3$. Un prélèvement de l'hématome lui-même sera également effectué par le chirurgien et un bilan biochimique (Glucose, Protéines, Osmolarité, D-Dimère, Fibrinogène, PT et PTT) sera effectué sur le spécimen en biochimie. Ces deux interventions n'altèrent aucunement la procédure chirurgicale.

À la fin de la chirurgie, l'équipe traitante devra remplir un questionnaire chirurgical précisant la procédure réalisée et l'utilisation de drains sous-duraux (cf. Annexe 12).

La séquence du reste du protocole et la chronologie des suivis demeurent par ailleurs inchangés. Aucun examen additionnel pré- ou post-opératoire n'est nécessaire, bien que ces derniers pourront être exécutés à la demande de l'équipe traitante au besoin, selon les critères cliniques usuels. Advenant une résolution complète de l'hématome lors d'un CT non-planifié au protocole, le médicament est cessé et le suivi post-traitement enclenché.

**Arrêt de l'intervention**

Si, après la randomisation, un sujet initialement éligible à l'étude développe une condition médicale rencontrant les critères d'exclusion de l'étude ou de cessation de la médication (par exemple, une thrombophlébite ou une fibrillation auriculaire), le patient est immédiatement contacté et l'intervention est cessée. Le suivi radiologique et clinique est toutefois poursuivi comme si le patient poursuivait l'intervention : les tomodensitométries axiales sont ainsi réalisées à 5, 10, 15 et 20 semaines jusqu'à résolution de l'hématome et les questionnaires remplis à 10 semaines. Le suivi final à 3 mois post-traitement est réalisé à partir de la date de fin de l'intervention. Si l'intervention est cessée à 5 semaines ou plus tôt, le suivi de 20 semaines est utilisé comme suivi 3 mois post-traitement et une tomodensitométrie axiale additionnelle n'est pas nécessaire.

Si, après la randomisation, il est établi que le sujet ne rencontrait pas les critères d'inclusion (par exemple, découverte d'hygrome sous-dural ou d'un empyème à la chirurgie au lieu d'un hématome sous-dural), l'intervention est immédiatement cessée et le patient est retiré de l'étude. Les questionnaires de suivi et les tomodensitométries axiales de contrôle sont annulées, car le suivi normal d'un hématome sous-dural chronique ne sera pas nécessairement le même que le suivi de la pathologie ayant mimé l'hématome.

**Thromboprophylaxie**

Tous les sujets de l'étude qui sont hospitalisés recevront une thromboprophylaxie standardisée consistant en des pompes à compression intermittente et de l'héparine 5 000 unités injectée en sous-cutané 2 fois par jour (ou 3 fois par jour si le patient a un poids > 100 kg) et devant être cessée 12h avant une intervention chirurgicale et reprise 12h après le retrait de tous les drains sous-duraux post-opératoires. L'équipe traitante peut modifier cette thromboprophylaxie au besoin si elle la juge inappropriée (par exemple, chez un patient avec antécédent de thrombocytopénie induite par l'héparine).

**Collecte des évènements indésirables**

Les évènements indésirables potentiellement en lien avec l'administration d'acide tranexamique seront systématiquement documentés lors des suivis cliniques planifiés en questionnant directement le patient.

Par ailleurs, les équipes traitantes doivent aviser le médecin coordonnateur du centre recruteur de tout évènement indésirable potentiellement en lien avec l'intervention dans un délai de 24h ouvrables. Le médecin coordonnateur du centre avisera à son tour l'investigateur principal de l'étude dans un délai de 5 jours ouvrables. L'investigateur principal sera responsable de rapporter à Santé Canada, dans un délai de 5 jours ouvrables, tout évènement à la fois grave (*Serious Adverse Event*), inattendu et possiblement en lien avec l'intervention.

Un évènement grave est un évènement rencontrant l'une des caractéristiques suivantes :

- Entraîne le décès du sujet;
- Met la vie du sujet en danger;
- Nécessite une hospitalisation ou entraîne la prolongation d'une hospitalisation;
- Entraîne une invalidité ou incapacité permanente ou importante;
- Se traduit par une anomalie ou malformation permanente, importante ou congénitale.

Un évènement inattendu est une complication de l'acide tranexamique non mentionnée dans la monographie du médicament (cf. Annexe 9).

Les centres recruteurs doivent avoir un mécanisme en place permettant aux équipes de recherche d'être avisées lorsqu'un de leur sujet est hospitalisé afin d'identifier proactivement la survenue d'évènements graves.

**Comité de sécurité**

Le comité de sécurité sera responsable d'analyser de façon indépendante des investigateurs la sécurité de l'intervention et les évènements indésirables survenant au long de l'étude. Le comité de sécurité se réunira une fois l'analyse intérimaire réalisée. Il pourra également être convoqué au besoin par les investigateurs si des préoccupations particulières étaient soulevées. Le comité de sécurité sera constitué, au minimum, de :

- Dr François Lamontagne, M.D., M.Sc., Service de traumatologie et soins critiques, Département de médecine, CHUS
- Dr Jean-François Castilloux, M.D., Service d'hématologie, Département de médecine, CHUS
- Dr Nicolas Dea, M.D., M.Sc., Service de neurochirurgie, Département de chirurgie, CHUS
- Dr David Fortin, M.D., M.Sc., Service de neurochirurgie, Département de chirurgie, CHUS

**Analyse intermédiaire pré-planifiée au 65$^{e}$ sujet**

Lorsque le suivi post-traitement sera enclenché pour le 65$^{e}$ sujet, une analyse intérimaire pré-planifiée aura lieu (cf. Annexe 11). Celle-ci visera à :

- Établir avec précision l'incidence de l'issue primaire dans notre échantillon;
- Réviser les calculs de puissance en fonction de l'incidence réelle de l'issue primaire;
- Établir l'incidence de complications thrombo-emboliques dans chacun des groupes (surveillance de la sécurité de l'intervention).

Suite aux résultats de l'analyse intérimaire pré-planifiée, une réunion des investigateurs aura lieu afin de :

- Débattre la nécessité de modifier les cibles de recrutement;
- Débattre la pertinence d'ouvrir l'étude à d'autres centres recruteurs.
- Réviser l'état du budget de projet;
- Débattre la nécessité d'obtenir davantage de financement;
- Analyser la survenue d'évènements indésirables;

Une copie du rapport de la réunion des investigateurs de même que de l'analyse intérimaire sera remise au comité de sécurité qui fournira des recommandations aux investigateurs afin d'améliorer, s'il y a lieu, la sécurité du protocole. Le comité de sécurité pourra exiger une

révision de l'étude par le Comité d'Éthique de la Recherche si des préoccupations quant à la sécurité de l'intervention étaient soulevées.

Dr Iorio-Morin sera responsable d'exécuter les recommandations posées suite à cette rencontre.

## Résumé du déroulement

**Jour -14 à 0 :**

- Tomodensitométrie diagnostique (réalisée hors du projet de recherche)
- Identification et recrutement du patient par l'équipe traitante

↓ Début du protocole

PHASE DE TRAITEMENT : Acide tranexamique 750 mg vs Placebo, 1,5 co PO die

**Jour 0 :**

- Questionnaire initial
- MMSE
- EQ-5D-5L et mBI
- Bilan sanguin
- Début traitement
- Tomodensitométrie injectée

**5 semaines* :**

- Tomodensitométrie

**10 semaines* :**

- Questionnaire de suivi
- MMSE
- EQ-5D-5L et mBI
- Service de Rx supplémentaires
- (Tomodensitométrie)

**15 semaines* :**

- (Tomodensitométrie)

**20 semaines* :**

- (Tomodensitométrie)

↓ Résolution complète de l'hématome ou fin des 20 semaines de traitement

**Si chirurgie :**

- Biopsie et analyse pathologique de la membrane de l'hématome
- Prélèvement et analyse biochimique de l'hématome
- Formulaire de protocole opératoire

PHASE DE SUIVI POST-TRAITEMENT

**Jour 0 :**

- Arrêt du traitement

**3 mois* :**

- Questionnaire de suivi
- MMSE
- EQ-5D-5L et mBI
- GOSE
- Tomodensitométrie

* Une fenêtre de +/- 7 jours est autorisée

## Méthode de collecte des données

Toutes les données recueillies dans le cadre de l'étude seront entreposées dans l'un et/ou l'autre des systèmes suivants :

- Le dossier de projet du patient :
  - Il s'agit d'une filière « papier » conservée sous clé au centre de recherche par l'infirmière de recherche;
  - Les documents contenus dans le dossier de projet incluent tous les formulaires « papiers », notamment :
    - Le formulaire de consentement signé;
    - La liste de validation du consentement;
    - Le questionnaire initial remplis en présence du patient;
    - Les questionnaires de suivi remplis en présence du patient;
    - Les grilles de test (MMSE, EQ-5D-5L, mBI, GOSE);

- Le système de gestion de projet en ligne :
  - Il s'agit d'une base de données informatisée, accessible en ligne et sécurisée par un identifiant et un mot de passe personnels;
  - Seule les infirmières de recherche et les médecins coordonateurs des centres recruteurs auront accès au système de gestion de projet en ligne;
  - Le système contiendra :
    - Les données nominatives de chaque sujet, incluant :
      - Nom et prénom;
      - Date de naissance;
      - Numéro de téléphone;
      - Numéro de dossier clinique;
    - Les résultats du bilan sanguin initial;
    - La transcription des résultats du questionnaire initial;
    - La transcription des résultats des questionnaires de suivi;
    - Les résultats des tests (MMSE, EQ-5D-5L, mBI, GOSE);
    - Le volume de l'hématome mesuré sur les différentes tomodensitométries;
    - Les résultats des analyses pathologique et biochimique de l'hématome.

- Le système permettra de :
  - Automatiser le suivi des patients en avisant l'infirmière de recherche par courriel lorsque :
    - Un sujet nécessite un suivi et/ou une tomodensitométrie;
    - Un sujet nécessite une livraison de médicaments supplémentaires;
  - Centraliser la collecte de données afin de permettre d'étendre l'étude à d'autres centres recruteurs au besoin;
  - Générer la base de données anonymisée avec laquelle sera exécutée l'analyse intermédiaire pré-planifiée au 65$^{e}$ sujet;
  - Générer la base de données anonymisée avec laquelle sera exécutée l'analyse finale;

Les données (dossier de projet et système de gestion de projet en ligne) seront conservées pour une durée de 25 ans après la date de publication des résultats de l'étude complétée.

## Analyse des données

Lorsque le dernier suivi du dernier sujet sera effectué, la table de randomisation des sujets au groupe A ou B sera dévoilée par la pharmacie et intégrée à la base de données en ligne. Un fichier Microsoft Excel contenant les données anonymisées sera généré et téléchargé par Dr Iorio-Morin afin d'effectuer les analyses statistiques dans le logiciel SPSS. Les données seront analysées en effectuant les tests statistiques présentés dans le tableau ci-dessous. Une fois les tableaux de résultat produits et les conclusions générales rédigées, l'intervention réalisée dans chaque groupe sera dévoilée par la pharmacie.

| Variable | Format utilisé pour l'analyse | Incertitude | Test statistique |
|---|---|---|---|
| **Comparaison des caractéristiques de l'étude pour les deux groupes (A vs B)** | | | |
| Nombre de sujets recrutés | Binaire | - | $\chi^2$ (Test exact de Fisher) |
| Nombre de sujets perdus au suivi | Binaire | - | $\chi^2$ (Test exact de Fisher) |
| **Comparaison des caractéristiques de base des deux groupes (A vs B)** | | | |
| Âge moyen au recrutement (années) | Continue | Écart-type | T de Student vs Mann-Withney * |
| Sexe (% d'hommes) | Continue | - | T de Student vs Mann-Withney * |
| Consommation moyenne d'alcool (consommation par semaine) | Continue | Écart-type | T de Student vs Mann-Withney * |
| Tabagisme actif (% de fumeurs actifs) | Continue | - | T de Student vs Mann-Withney * |
| Tabagisme cumulatif moyen (paquets-années) | Continue | Écart-type | T de Student vs Mann-Withney * |
| Antécédent d'hématome sous-dural (% ayant un antécédent d'hématome sous-dural) | Continue | - | T de Student vs Mann-Withney * |
| Prise récente d'un médicament antiplaquettaire (% avec prise récente) | Continue | - | T de Student vs Mann-Withney * |
| Prise récente d'un médicament anticoagulant (% avec prise récente) | Continue | - | T de Student vs Mann-Withney * |
| Prise récente d'IECA ou d'ARA (% avec prise récente) | Continue | - | T de Student vs Mann-Withney * |
| Prise récente de corticostéroïde (% avec prise récente) | Continue | - | T de Student vs Mann-Withney * |
| Convulsion dans la dernière année (% avec convulsion) | Continue | - | T de Student vs Mann-Withney * |
| Dérivation de LCR dans la dernière année (% avec dérivation) | Continue | - | T de Student vs Mann-Withney * |

| | | | |
|---|---|---|---|
| Antécédent de procédure neurochirurgicale récente (% avec procédure) | Continue | - | T de Student vs Mann-Withney * |
| Histoire de traumatisme récent (% avec traumatisme) | Continue | - | T de Student vs Mann-Withney * |
| Score MMSE moyen au recrutement | Continue | Écart-type | T de Student vs Mann-Withney * |
| Score EQ-5D-5L moyen au recrutement | Continue | Écart-type | T de Student vs Mann-Withney * |
| Score mBI moyen au recrutement | Continue | Écart-type | T de Student vs Mann-Withney * |
| Plan initial de traitement (% de traitement conservateur comme choix initial) | Continue | - | T de Student vs Mann-Withney * |
| Hémoglobine moyenne au diagnostic | Continue | Écart-type | T de Student vs Mann-Withney * |
| Décompte plaquettaire moyen au diagnostic | Continue | Écart-type | T de Student vs Mann-Withney * |
| PT moyen au diagnostic | Continue | Écart-type | T de Student vs Mann-Withney * |
| PTT moyen au diagnostic | Continue | Écart-type | T de Student vs Mann-Withney * |
| Insuffisance rénale (% des sujets ayant un DFGe < 60 mL/min) | Continue | - | T de Student vs Mann-Withney * |
| Ratio bicaudé moyen | Continue | Écart-type | T de Student vs Mann-Withney * |
| Hématome supratentoriel à la convexité (% des sujets) | Continue | - | T de Student vs Mann-Withney * |
| Hématome supratentoriel interhémisphérique (% des sujets) | Continue | - | T de Student vs Mann-Withney * |
| Hématome infratentoriel (% des sujets) | Continue | - | T de Student vs Mann-Withney * |
| Hématome du côté dominant (% des sujets) | Continue | - | T de Student vs Mann-Withney * |
| Volume moyen de l'hématome au diagnostic (mL) | Continue | Écart-type | T de Student vs Mann-Withney * |
| Composition de l'hématome sous-dural intial (% des sujets ayant un hématome purement chronique) | Continue | - | T de Student vs Mann-Withney * |
| **Effet de l'intervention (A vs B)** | | | |
| Atteinte de l'issue primaire | Binaire | - | $\chi^2$ (Test exact de Fisher) |
| Variation du volume de l'hématome | Série de valeurs continues | - | ANOVA à mesures répétées |
| Résolution de l'hématome | Binaire | - | $\chi^2$ (Test exact de Fisher) |
| Récidive d'hématome sous-dural | Binaire | - | $\chi^2$ (Test exact de Fisher) |
| Drainage chirurgical | Binaire | - | $\chi^2$ (Test exact de Fisher) |

| | | | |
|---|---|---|---|
| Variation du score MMSE | Série de 3 valeurs continues | - | ANOVA à mesures répétées |
| Variation du score EQ-5D-5L | Série de 3 valeurs continues | - | ANOVA à mesures répétées |
| Variation du score mBI | Série de 3 valeurs continues | - | ANOVA à mesures répétées |
| Score GOSE final | Continue | - | T de Student vs Mann-Withney * |
| Durée de l'hospitalisation initiale | Continue | - | T de Student vs Mann-Withney * |
| Nombre de ré-hospitalisation pour hématome sous-dural | Continue | - | T de Student vs Mann-Withney * |
| **Facteurs de risque pour l'issue primaire (issue primaire atteinte vs non-atteinte) pour l'ensemble des sujets** | | | |
| Facteurs de risques évalués :<br>• Traitement administré (placébo vs acide tranexamique)<br>• Âge<br>• Consommation d'alcool<br>• Tabagisme actif<br>• Tabagisme cumulatif<br>• Antécédent d'hématome sous-dural<br>• Médication antiplaquettaire<br>• Médication anticoagulante<br>• IECA ou ARA<br>• Corticostéroïdes<br>• Convulsions récentes<br>• Dérivation de LCR récente<br>• Procédure neurochirurgicale récente<br>• Décompte plaquettaire moyen au diagnostic<br>• PT au diagnostic<br>• PTT au diagnostic<br>• Insuffisance rénale<br>• Ratio bicaudé<br>• Localisation de l'hématome<br>• Volume initial de l'hématome<br>• Composition initiale de l'hématome<br>• Vascularisation de la membrane<br>• Rehaussement de l'hématome | - | - | Régression logistique |
| **Facteurs de risque pour l'issue primaire (issue primaire atteinte vs non-atteinte) pour les sujets du groupe Acide tranexamique uniquement** | | | |
| Facteurs de risques évalués :<br>• Vascularisation de la membrane<br>• Rehaussement de l'hématome<br>• Ratio des dosage hématome/sang pour :<br>o Glucose<br>o Protéines<br>o Osmolarité<br>o PT<br>o PTT<br>o Fibrinogène | - | - | Régression logistique |

| | | | |
|---|---|---|---|
| o D-dimères<br>• Composition de l'hématome | | | |
| **Sécurité de l'intervention (groupe A vs B)** | | | |
| Incidence de thrombophlébite profonde | Binaire | - | $\chi^2$ (Test exact de Fisher) |
| Incidence d'embolie pulmonaire | Binaire | - | $\chi^2$ (Test exact de Fisher) |
| Incidence d'ICT | Binaire | - | $\chi^2$ (Test exact de Fisher) |
| Incidence d'AVC | Binaire | - | $\chi^2$ (Test exact de Fisher) |
| Incidence de pseudo-néphrolithiase | Binaire | - | $\chi^2$ (Test exact de Fisher) |

* Pour évaluer l'effet de variables continues sur une issue dichotomique, le test T de Student sera employé si la distribution est normale, alors que le U de Mann-Whitney sera utilisé si la distribution est anormale. La normalité de la distribution sera évaluée avec le test de Kolmogorov-Smirnov (si l'on dispose de ≥ 50 observations) ou de Shapiro-Wilk (si l'on dispose de < de 50 observations).

## Limites de l'étude

L'étude a été conçue afin de minimiser au maximum les biais potentiels. Ainsi :

- Concernant la validité interne :
  - o Biais de sélection du groupe contrôle secondaire à la randomisation :
    - ▪ Vu la petite taille de l'étude, il est possible que, malgré la randomisation, le groupe contrôle ne soit pas parfaitement similaire au groupe intervention. Une telle situation diminuerait la puissance de l'étude et rendrait probablement impossibles les analyses de sous-groupe. L'usage d'une randomisation stratifiée pour la prise d'antiplaquettaires permettra d'assurer minimalement un contrôle de ces facteurs, jugés les plus importants.

- Concernant la validité externe :
  - o Biais de sélection secondaire à un échantillonnage non-probabiliste :
    - ▪ L'usage de critères d'inclusion et d'exclusion stricts et objectifs devrait permettre de maintenir un échantillon représentatif de la population à l'étude.
  - o Biais de sélection secondaire à la perte au suivi :
    - ▪ Il est inévitable que certains sujets seront perdus au suivi. Des efforts considérables ont été investis afin de minimiser la durée du suivi (4 mois pour l'issue primaire, 7 mois au maximum pour le suivi complet).

## Considérations éthiques

### Obtention d'un consentement éclairé

Le consentement au projet sera libre et éclairé. Spécifiquement :

- Lors des heures ouvrables, le recrutement sera fait par une infirmière de recherche indépendante de l'équipe traitante.
- Lors des soirs et des fins de semaines, le consentement initial sera obtenu par un membre de l'équipe traitante, mais sera par la suite validé par l'infirmière de recherche lors des heures ouvrables afin d'en assurer la qualité et la liberté.
- Aucune pression ne sera exercée sur le patient pour sa participation à l'étude.
- Les sujets peuvent se retirer du protocole à tout moment.
- La participation au protocole n'altère aucunement la prise en charge du patient, ni son accès aux tests diagnostiques et plateaux techniques nécessaires à un traitement optimal de sa condition.

Pour les patients inaptes à consentir, un substitut à la prise de décision est impliqué pour fournir le consentement. Il s'agira du proche jugé le plus significatif par l'équipe traitante (p. ex. conjoint, enfant, ami) et non-nécessairement le mandataire ou le représentant légal. Par ailleurs, même s'il est inapte, le patient peut refuser de participer au projet de recherche indépendamment de l'opinion de ses proches.

### Risques reliés à la participation à l'étude

Les principaux risques auxquels seront exposés les sujets sont des risques liés à leur condition médicale (hématome sous-dural) indépendamment de leur participation à l'étude. Ces risques incluent notamment les risques liés à la tomodensitométrie axiale et à la chirurgie de drainage d'hématome qui pourrait avoir lieu. Ces risques ne seront pas spécifiquement discutés dans le formulaire de consentement, car ils ne sont pas altérés par la participation à l'étude.

Les risques reliés à la participation à l'étude sont :

- Risques reliés à l'administration d'acide tranexamique (pour les sujets dans le groupe intervention). Ce médicament est bien connu, approuvé par Santé Canada et très bien toléré. Les effets secondaires rapportés incluent :
    - Thrombose veineuse ou artérielle
    - Rétention intra-rénale de caillots chez les sujets souffrant d'hémorragie rénale massive
    - Convulsions
    - Nausées
    - Étourdissements
    - Dermatite allergique

- Risques reliés à l'injection d'iode (pour les sujets n'ayant eu de tomographie axiale injectée au moment du diagnostic) :
    - Allergie au produit de contraste
    - Néphropathie de contraste chez les sujets préalablement insuffisant rénaux

Les risques reliés à la tomodensitométrie axiale ou à la chirurgie de drainage d'hématome sous-dural ne sont pas inhérents à l'étude et ne sont donc pas discutés. De plus, le prélèvement per-opératoire d'un échantillon de l'hématome sous-dural de même que le prélèvement d'une

biopsie de la membrane externe de l'hématome n'altèrent aucunement la procédure chirurgicale et ne posent aucun risque supplémentaire.

### Inconvénients liés à la participation à l'étude

Les sujets devront rencontrer l'infirmière de recherche à 3 reprises pour répondre à des questionnaires nécessitant la présence physique du patient (MMSE, EQ-5D-5L). Afin de minimiser les inconvénients pour le patient, ces rencontres seront réalisées immédiatement avant ou après les tomodensitométries prévues au protocole (et qui seraient par ailleurs nécessaires pour tout suivi régulier d'hématome sous-dural).

Si l'hématome sous-dural n'est pas résolu radiologiquement à la tomodensitométrie de 10 semaines, le sujet devra se présenter à son centre recruteur pour obtenir un deuxième service de médicaments.

### Respect de la confidentialité

La confidentialité sera strictement respectée tout au long de l'étude. Spécifiquement, l'usage du système de gestion de projet en ligne permettra de conserver à un endroit unique les données nominatives (nom et numéro de dossier clinique) dans un entrepôt numérique sécurisé. Le système ne permettra pas d'enregistrer les données sur un ordinateur personnel ou sur une clé USB sujette à perte, et génèrera automatiquement des fichiers d'analyse dénominalisés. Seul l'infirmière de recherche et les médecins coordonnateurs des centres recruteurs auront accès aux données nominatives et aucune donnée nominative ne sera écrite dans le dossier de projet « papier ».

### Indemnisation des participants

Les sujets ne recevront aucune indemnisation monétaire pour leur participation à l'étude. Ils n'auront toutefois aucun frais à débourser pour les médicaments consommés dans le cadre de l'étude.

## Retombées anticipées

En inhibant la fibrinolyse, nous postulons que l'acide tranexamique pourrait traiter les hématomes sous-duraux de façon médicale. Si notre étude permet de confirmer cette hypothèse, il sera possible d'offrir aux patients atteints d'hématome sous-duraux une alternative à la chirurgie, diminuant du coup la morbidité du traitement de cette pathologie.

## Diffusion des résultats

L'étude est enregistrée dans la banque d'études cliniques clinicaltrial.gov (NCT02568124) afin de déclarer son existence et diminuer les biais de non-publication pour les études négatives (23). Un résumé anglophone du protocole de même le protocole francophone complet a également été accepté pour publication dans la revue *Trials*.

Au terme de l'étude, les résultats de l'analyse seront publiés dans une revue spécialisée. Ces résultats seront également présentés à plusieurs congrès de neurochirurgie, tant localement qu'à l'international.

## Échéancier

| Échéance | Objectif |
|---|---|
| **Avril 2014** | • Obtention du financement PAFI<br>• Demande de bourse au département de chirurgie |
| **Novembre 2014** | • Dépôt du protocole au comité scientifique |
| **Décembre 2014** | • Acceptation au comité scientifique<br>• Dépôt au comité d'éthique<br>• Embauche de l'infirmière de recherche |
| **Mars 2015** | • Acceptation au comité d'éthique<br>• Commande des médicaments et placebo<br>• Enregistrement de l'étude sur clinicaltrial.gov<br>• Présentation du protocole final aux membres du service de neurochirurgie |
| **Avril 2015** | • Configuration du système de gestion de projet en ligne<br>• Traduction anglaise du protocole<br>• Implantation des procédures du protocoles au CHUS |
| **Octobre 2015** | • Début du recrutement au CHUS<br>• Publication du protocole approuvée par la revue *Trials*<br>• Obtention du financement de la Fondation Neuro-Trauma Marie Robert pour ouvrir le recrutement au CHa |
| **Juin 2016** | • Génération des modèles de fichiers d'analyse<br>• Programmation SPSS de l'analyse pré-planifiée au 65$^{e}$ sujet<br>• Programmation SPSS de l'analyse finale<br>• Ouverture du recrutement au CHa |
| **Janvier 2017** | • Suivi 4 mois du 65$^{e}$ sujet<br>• Analyse intérimaire pré-planifiée<br>• Révision des calculs de puissance<br>• Réunion des investigateurs |
| **Janvier 2018** | • Recrutement du 130$^{e}$ sujet<br>• Fin du recrutement<br>• Début de l'analyse des tomodensitométries axiales |
| **Mai 2018** | • Suivi final du dernier sujet<br>• Analyse finale<br>• Rédaction du manuscrit |
| **Octobre 2018** | • Soumission du manuscrit pour publication |

# Budget

Pour le budget détaillé, se référer au budget CRC-RC1.

Le financement sera initialement assumé par une bourse PAFI de 15 000$ octroyée le 23 janvier 2014 et expirant le 31 mars 2018. Ce montant est suffisant pour couvrir les frais de médicaments de 90 sujets (si tous les sujets utilisent le maximum de comprimés possible). Tel que présenté dans le budget, près du quart du coût du projet est consommé par les « frais administratifs » de la pharmacie du CRCHUS. Des négociations sont en cours afin d'atténuer ce fardeau.

Par ailleurs, une bourse de 4 000$ du département de chirurgie permettra également l'embauche d'une infirmière de recherche dédiée au projet. En cas de précarité budgétaire, les tâches de l'infirmière de recherche seront accomplies de façon temporaire par Dr Iorio-Morin.

Une bourse de 20 000$ octroyée par la Fondation Neuro-trauma Marie Robert financera la conduite du projet au CHa.

## Références

1. Jourdain A. Hospitalisations au CHUS pour hématome sous-dural non-traumatique entre 2009 et 2012. 1st ed. Sherbrooke: InfoCentre; 2013 Nov. Report No.: IC-2013-002366.

2. Jourdain A. Hospitalisations au CHUS pour hématome sous-dural traumatique entre 2009 et 2012. 2nd ed. Sherbrooke: InfoCentre; 2014 Mar. Report No.: IC-2013-002366.

3. O'Neill B, Wilberger J, Wilberger A. Pathophysiology of Subdural Hematomas. In: Couldwell WT, Winn HR, editors. Youmans Neurological Surgery. 6 ed. Elsevier; 2011. pp. 532–4.

4. Horikoshi T, Naganuma H, Fukasawa I, Uchida M, Nukui H. Computed tomography characteristics suggestive of spontaneous resolution of chronic subdural hematoma. Neurol Med Chir (Tokyo). 1998 Sep;38(9):527–32–discussion532–3.

5. Sambasivan M. An overview of chronic subdural hematoma: experience with 2300 cases. Surg Neurol. 1997 May;47(5):418–22.

6. Oh H-J, Lee K-S, Shim J-J, Yoon S-M, Yun I-G, Bae H-G. Postoperative course and recurrence of chronic subdural hematoma. J Korean Neurosurg Soc. 2010 Dec;48(6):518–23.

7. Krauss JK, Marshall LF, Weigel R. Medical and Surgical Management of Chronic Subdural Hematomas. In: Couldwell WT, Winn HR, editors. Youmans Neurological Surgery. 6 ed. Elsevier; 2011. pp. 535–43.

8. Almenawer SA, Farrokhyar F, Hong C, Alhazzani W, Manoranjan B, Yarascavitch B, et al. Chronic Subdural Hematoma Management: A Systematic Review and Meta-analysis of 34829 Patients. Ann Surg. 2013 Oct 3.

9. Weigel R, Hohenstein A, Schlickum L, Weiss C, Schilling L. Angiotensin converting enzyme inhibition for arterial hypertension reduces the risk of recurrence in patients with chronic subdural hematoma possibly by an antiangiogenic mechanism. Neurosurgery. 2007 Oct;61(4):788–92–discussion792–3.

10. CRASH-2 trial collaborators, Shakur H, Roberts I, Bautista R, Caballero J, Coats T, et al. Effects of tranexamic acid on death, vascular occlusive events, and blood transfusion in trauma patients with significant haemorrhage (CRASH-2): a randomised, placebo-controlled trial. Lancet. 2010 Jul 3;376(9734):23–32.

11. Ker K, Edwards P, Perel P, Shakur H, Roberts I. Effect of tranexamic acid on surgical bleeding: systematic review and cumulative meta-analysis. BMJ. 2012;344:e3054.

12. Wellington K, Wagstaff AJ. Tranexamic acid: a review of its use in the management of menorrhagia. Drugs. 2003;63(13):1417–33.

13. Vujkovac B, Sabovic M. Treatment of subdural and intracerebral haematomas in a haemodialysis patient with tranexamic acid. Nephrol Dial Transplant. 2000 Jan;15(1):107–9.

14. Kageyama H, Toyooka T, Tsuzuki N, Oka K. Nonsurgical treatment of chronic subdural hematoma with tranexamic acid. J Neurosurg. 2013 Aug;119(2):332–7.

15. Nakaguchi H, Tanishima T, Yoshimasu N. Factors in the natural history of chronic subdural hematomas that influence their postoperative recurrence. J Neurosurg. 2001 Aug;95(2):256–62.

16. Tsai FY, Huprich JE, Segall HD, Teal JS. The contrast-enhanced CT scan in the diagnosis of isodense subdural hematoma. J Neurosurg. 1979 Jan;50(1):64–9.

17. Park H-R, Lee K-S, Shim J-J, Yoon S-M, Bae H-G, Doh J-W. Multiple Densities of the Chronic Subdural Hematoma in CT Scans. J Korean Neurosurg Soc. 2013 Jul;54(1):38–41.

18. Olson RA, Iverson GL, Carolan H, Parkinson M, Brooks BL, McKenzie M. Prospective comparison of two cognitive screening tests: diagnostic accuracy and correlation with community integration and quality of life. J Neurooncol. 2011 Nov;105(2):337–44.

19. Ismail Z, Mulsant BH, Herrmann N, Rapoport M, Nilsson M, Shulman K. Canadian academy of geriatric psychiatry survey of brief cognitive screening instruments. Can Geriatr J. 2013;16(2):54–60.

20. Goffaux P, Boudrias M, Mathieu D, Charpentier C, Veilleux N, Fortin D. Development of a concise QOL questionnaire for brain tumor patients. Can J Neurol Sci. 2009 May;36(3):340–8.

21. Shah S, Vanclay F, Cooper B. Improving the sensitivity of the Barthel Index for stroke rehabilitation. J Clin Epidemiol. 1989;42(8):703–9.

22. Yang M, Ding X, Dong B. The measurement of disability in the elderly: a systematic review of self-reported questionnaires. J Am Med Dir Assoc. 2014 Feb;15(2):150.e1–9.

23. De Angelis C, Drazen JM, Frizelle FA, Haug C, Hoey J, Horton R, et al. Clinical trial registration: a statement from the International Committee of Medical Journal Editors. N Engl J Med. 2004 Sep 16;351(12):1250–1.

## Signatures

Dr Christian Iorio-Morin, résident 3
Service de neurochirurgie, Département de chirurgie
Centre hospitalier universitaire de Sherbrooke
Faculté de médecine et des sciences de la santé
Université de Sherbrooke

Dr Jocelyn Blanchard
Service de neurochirurgie, Département de chirurgie
Centre hospitalier universitaire de Sherbrooke
Faculté de médecine et des sciences de la santé
Université de Sherbrooke

Dr David Mathieu
Service de neurochirurgie, Département de chirurgie
Centre hospitalier universitaire de Sherbrooke
Centre de recherche du CHUS
Faculté de médecine et des sciences de la santé
Université de Sherbrooke

# Annexe 1

# Liste de validation du processus de consentement éclairé

# L'acide tranexamique comme traitement médical des hématomes sous-duraux chroniques

## Liste de validation du processus de consentement éclairé

Numéro du sujet: ______________________________

Je certifie que :

- ☐ Sujet approché : ____________________________ (sujet ou proche significatif offrant le consentement) dans le cadre du protocole.
- ☐ Sujet accepte de rencontrer l'équipe de recherche.
- ☐ Étude expliquée et sujet semble bien comprendre.
- ☐ Consentement lu ou j'ai lu le consentement au sujet.
  - o Si un pré-consentement a été fait → Validation de la compréhension
  - o Si aucun pré-consentement fait → Explications détaillées
- ☐ Avons répondu de façon satisfaisante à toutes ses questions.
- ☐ Sujet d'accord pour participation au protocole suite à une décision libre et éclairée.
- ☐ Consentement signé par le sujet, **version du** : _______________.
- ☐ Consentement signé préalablement à toutes procédures relatives à l'étude.
  - o Oui
  - o Non, car pré-consentement obtenu
- ☐ Copie signée du consentement remise au sujet.

___________________________________________________________

Signature de la personne qui a obtenu le consentement — Date de la signature

# Annexe 2

# Questionnaire initial

# L'acide tranexamique comme traitement médical des hématomes sous-duraux chroniques

## Questionnaire initial

## Identification

No sujet ______________________  No dossier ______________________

Nom ______________________  Prénom ______________________

Sexe ☐ Femme ☐ Homme  Date de naissance ______________________

Centre recruteur ______________________  Date du recrutement ______________________

## Caractéristiques de base

| | |
|---|---|
| Dominance | ☐ Droitier<br>☐ Gaucher<br>☐ Ambidextre |
| Consommation d'alcool (nombre par semaine) | ______________________ |
| Tabagisme actif | ☐ Non<br>☐ Oui |
| Tabagisme cumulatif (nombre de paquets-années) | ______________________ |
| Antécédent d'hématome sous-dural | ☐ Non<br>☐ Oui, non-drainé<br>☐ Oui, drainé |

| | |
|---|---|
| Prise récente (moins de 1 mois) d'un médicament antiplaquettaire | ☐ Non<br>☐ Oui : ________________ |
| Prise récente (moins de 1 mois) d'un médicament anticoagulant | ☐ Non<br>☐ Oui : ________________ |
| Prise récente (moins de 1 mois) d'IECA ou D'ARA | ☐ Non<br>☐ Oui : ________________ |
| Prise récente (moins de 1 mois) de corticostéroïde | ☐ Non<br>☐ Oui |
| Convulsion dans la dernière année (nombre) | ______________________________ |
| Dérivation de LCR dans la dernière année | ☐ Non<br>☐ Oui, ponction lombaire<br>☐ Oui, dérivation ventriculo-péritonéale<br>☐ Oui, autre : _______________ |
| Antécédent de procédure neurochirurgicale récente (moins de 1 mois avant le début des symptômes) | ☐ Non<br>☐ Oui, procédure extra-crânienne<br>☐ Oui, procédure intra-crânienne |
| Histoire de traumatisme récent (moins de 1 mois avant le début des symptômes) | ☐ Non<br>☐ Oui, mineur (pas de diagnostic)<br>☐ Oui, majeur (TCC léger ou pire diagnostiqué par un MD) |
| Tout autre facteur de risque d'hématome sous-dural chronique jugé pertinent | ______________________________ |

## Présentation clinique

| | |
|---|---|
| Contexte de la consultation | ☐ Consultation élective en clinique externe<br>☐ Consultation non-urgente à l'urgence ou sur un étage<br>☐ Consultation urgente |
| Symptôme **principal** à la présentation<br>(ne cocher qu'un seul symptôme) | ☐ Asymptomatique<br>☐ Céphalées<br>☐ Confusion<br>☐ Somnolence<br>☐ Chute ou trouble d'équilibre<br>☐ Faiblesse d'un membre<br>☐ Trouble du langage<br>☐ Trouble de mémoire<br>☐ Nausées/Vomissements<br>☐ Trouble visuel<br>☐ Convulsion<br>☐ Autre |
| Date du début approximatif des symptômes | ______________________ |
| Score MMSE au recrutement | ______________________ |
| Score mBI au recrutement | ______________________ |
| Score EQ-5D détaillé (XXXXX) au recrutement | ______________________ |
| Score EQ-5D VAS au recrutement | ______________________ |
| Plan initial de traitement | ☐ Conservateur<br>☐ Drainage électif<br>☐ Drainage urgent (ne pouvant pas attendre au lendemain) |

# Annexe 3

# Formulaire de validation du pré-consentement par les résidents

# L'acide tranexamique comme traitement médical des hématomes sous-duraux chroniques

## FORMULAIRE DE VALIDATION DU PRÉ-CONSENTEMENT OBTENU PAR L'ÉQUIPE TRAITANTE

Numéro du sujet: ______________________________

Je certifie que :

- ☐ Sujet approché : ___________________________ (sujet ou proche significatif offrant le consentement) dans le cadre du protocole.
- ☐ J'ai expliqué l'étude en détail au patient.
- ☐ J'ai expliqué la définition d'un placébo.
- ☐ J'ai expliqué les effets secondaires potentiels du traitement.
- ☐ J'ai expliqué la nécessité de tomodensitométries axiales de contrôle et les risques associés.
- ☐ J'ai répondu à toutes les questions du sujet.
- ☐ Le sujet comprend et accepte de rencontrer l'équipe de recherche.
- ☐ **Si une chirurgie urgente est nécessaire**, le sujet accepte de débuter l'étude immédiatement et de rencontrer l'équipe de recherche lors du prochain jour ouvrable. Le traitement ne sera débuté qu'après la signature du formulaire de consentement, mais les premiers questionnaires, scan et prélèvements devant être fait avant la chirurgie pourront être faits avant la signature du formulaire de consentement.

_______________________________________________________________

Signature de la personne qui a obtenu le consentement — Date de la signature

# Annexe 4

# Mini-Mental State Examination (MMSE)

# EXAMEN DE FOLSTEIN SUR L'ÉTAT MENTAL

DT9088

Nom de l'établissement ______________________________

## A) Orientation

| | COTE MAXIMALE | COTE DU SUJET |
|---|---|---|
| Demander au sujet : | | |
| **1- Quel est** l'année ___ le mois ___ le jour ___ le jour de la semaine : D L M Me J V S<br>La saison : printemps ☐ été ☐ automne ☐ hiver ☐ | **5** | ( ) |
| **2- Où sommes-nous** Province ___ Pays ___<br>Ville, village ___ Lieu (hôpital, cabinet, maison, etc.) ___ Étage ___ | **5** | ( ) |

## B) Enregistrement

3- Mentionner un des groupes de 3 mots suivants; prendre une seconde pour prononcer chaque mot :

| | COTE MAXIMALE | COTE DU SUJET |
|---|---|---|
| chemise, bleu, honnêteté ( )<br>ou<br>chaussure, brun, modestie ( )<br>ou<br>chandail, blanc, charité ( ) | **3** | ( ) |

Par la suite, demander au sujet de répéter les 3 mots choisis.

Donner 1 point pour chaque bonne réponse au 1er essai. Répéter l'exercice jusqu'à ce que le sujet retienne les 3 mots. Compter le nombre d'essais et le noter. Pour information seulement.

Nombre d'essais : ______

## C) Attention et calcul (cocher l'un ou l'autre test)

| | COTE MAXIMALE | COTE DU SUJET |
|---|---|---|
| 4- ☐ Demander au sujet de faire la soustraction par intervalles de 7 à partir de 100 :<br>100 – 7 = ( ) 93 – 7 = ( ) 86 – 7 = ( ) 79 – 7 = ( ) 72 – 7 = ( ) 65.<br>Donner 1 point pour chaque bonne réponse.<br>**OU**<br>☐ Demander au sujet d'épeler le mot « MONDE » à l'envers. (EDNOM) : ___ ___ ___ ___ ___ (écrire les lettres) | **5** | ( ) |

## D) Rétention mnésique

5- Demander au sujet de répéter les 3 mots déjà mentionnés :

| | COTE MAXIMALE | COTE DU SUJET |
|---|---|---|
| chemise, bleu, honnêteté ( )<br>ou<br>chaussure, brun, modestie ( )<br>ou<br>chandail, blanc, charité ( ) | **3** | ( ) |

## E) Langage

| | COTE MAXIMALE | COTE DU SUJET |
|---|---|---|
| 6- Montrer au sujet un crayon ( ) une montre ( ) et lui demander de nommer l'objet. | **2** | ( ) |
| 7- Demander au sujet de répéter la phrase suivante : **« Pas de si ni de mais »**. | **1** | ( ) |
| 8- Demander au sujet d'obéir à un ordre en 3 temps : **« Prenez ce papier de la main droite ou gauche, pliez-le en deux et redonnez-le moi ».** | **3** | ( ) |

***N.B. :*** *Demander au sujet droitier de prendre de la main gauche et vice versa. Prendre garde de tendre la main; éviter les indices non-verbaux.*

Traduction et adaptation française non validées du « Mini-Mental State » de Folstein, M.F., Folstein, S.E., Mc Hugh, P.R. : « Mini-Mental State : A practical method for grading the cognitive state of patients for the clinician », J. Psychiatr Res, 12 : 189-198, 1975, à partir des travaux du centre de gériatrie Hôpital d'Youville Sherbrooke de Québec.



(suite au verso)

| Nom de l'usager | N° de dossier |
|---|---|
| | |

## E) Langage (suite)

| | COTE MAXIMALE | COTE DU SUJET |
|---|---|---|
| 9- Demander au sujet de lire et de suivre l'instruction suivante : **« FERMEZ VOS YEUX »** | 1 | ( ) |
| 10- Demander au sujet d'écrire une phrase : ______ (sujet, verbe, sans égard aux fautes) | 1 | ( ) |

## F) Praxie de construction

11- Demander au sujet de copier le dessin suivant :

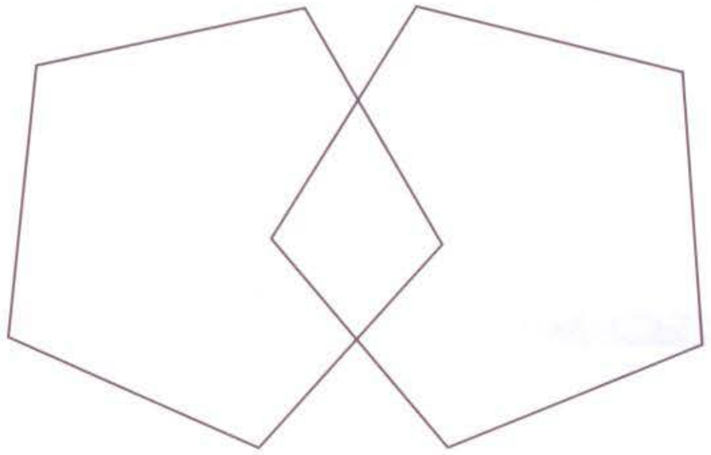

| | COTE MAXIMALE | COTE DU SUJET |
|---|---|---|
| 11- | 1 | ( ) |

**COTE MAXIMALE : 30**

**COTE DU SUJET :** ______

***Interprétation des scores :***
***Un score de moins de 24 points à l'examen de Folstein sur l'état mental détermine une altération des fonctions cognitives. Ce test ne permet cependant pas de faire un diagnostic étiologique.***

Nombre d'années de scolarité du sujet : ______ Évaluer le niveau de conscience du sujet : vigilant ☐ somnolent ☐

En cas d'incapacité du sujet à être évalué, spécifier : ______

Indiquer les conditions ayant pu influencer l'évaluation ______

Date | Année | Mois | Jour |  ______ Signature

# Annexe 5

# EQ-5D-5L

Pour chaque rubrique, veuillez cocher UNE case, celle qui décrit le mieux votre santé AUJOURD'HUI.

**MOBILITÉ**

Je n'ai aucun problème pour me déplacer à pied ❑
J'ai des problèmes légers pour me déplacer à pied ❑
J'ai des problèmes modérés pour me déplacer à pied ❑
J'ai des problèmes sévères pour me déplacer à pied ❑
Je suis incapable de me déplacer à pied ❑

**AUTONOMIE DE LA PERSONNE**

Je n'ai aucun problème pour me laver ou m'habiller tout(e) seul(e) ❑
J'ai des problèmes légers pour me laver ou m'habiller tout(e) seul(e) ❑
J'ai des problèmes modérés pour me laver ou m'habiller tout(e) seul(e) ❑
J'ai des problèmes sévères pour me laver ou m'habiller tout(e) seul(e) ❑
Je suis incapable de me laver ou de m'habiller tout(e) seul(e) ❑

**ACTIVITÉS COURANTES** *(exemples: travail, études, travaux domestiques, activités familiales ou loisirs)*

Je n'ai aucun problème pour accomplir mes activités courantes ❑
J'ai des problèmes légers pour accomplir mes activités courantes ❑
J'ai des problèmes modérés pour accomplir mes activités courantes ❑
J'ai des problèmes sévères pour accomplir mes activités courantes ❑
Je suis incapable d'accomplir mes activités courantes ❑

**DOULEURS / INCONFORT**

Je n'ai ni douleur ni inconfort ❑
J'ai des douleurs ou un inconfort léger(ères) ❑
J'ai des douleurs ou un inconfort modéré(es) ❑
J'ai des douleurs ou un inconfort sévère(s) ❑
J'ai des douleurs ou un inconfort extrême(s) ❑

**ANXIÉTÉ / DÉPRESSION**

Je ne suis ni anxieux(se) ni déprimé(e) ❑
Je suis légèrement anxieux(se) ou déprimé(e) ❑
Je suis modérément anxieux(se) ou déprimé(e) ❑
Je suis sévèrement anxieux(se) ou déprimé(e) ❑
Je suis extrêmement anxieux(se) ou déprimé(e) ❑



*Canada (French) © 2009 EuroQol Group EQ-5D™ is a trade mark of the EuroQol Group*

EQ-5D-5L

- Nous aimerions savoir dans quelle mesure votre santé est bonne ou mauvaise AUJOURD’HUI.

- Cette échelle est numérotée de 0 à 100.

- 100 correspond à la meilleure santé que vous puissiez imaginer.
0 correspond à la pire santé que vous puissiez imaginer.

- Veuillez faire un X sur l’échelle afin d’indiquer votre état de santé AUJOURD’HUI.

- Maintenant, veuillez noter dans la case ci-dessous le chiffre que vous avez coché sur l’échelle.

VOTRE SANTÉ AUJOURD’HUI = ☐

Date: ____________________

La meilleure santé que vous puissiez imaginer

100
95
90
85
80
75
70
65
60
55
50
45
40
35
30
25
20
15
10
5
0

La pire santé que vous puissiez imaginer



*Canada (French) © 2009 EuroQol Group EQ-5D™ is a trade mark of the EuroQol Group*

EQ-5D-5L

Under each heading, please tick the ONE box that best describes your health TODAY.

**MOBILITY**

I have no problems in walking about ❑
I have slight problems in walking about ❑
I have moderate problems in walking about ❑
I have severe problems in walking about ❑
I am unable to walk about ❑

**SELF-CARE**

I have no problems washing or dressing myself ❑
I have slight problems washing or dressing myself ❑
I have moderate problems washing or dressing myself ❑
I have severe problems washing or dressing myself ❑
I am unable to wash or dress myself ❑

**USUAL ACTIVITIES** *(e.g. work, study, housework, family or leisure activities)*

I have no problems doing my usual activities ❑
I have slight problems doing my usual activities ❑
I have moderate problems doing my usual activities ❑
I have severe problems doing my usual activities ❑
I am unable to do my usual activities ❑

**PAIN / DISCOMFORT**

I have no pain or discomfort ❑
I have slight pain or discomfort ❑
I have moderate pain or discomfort ❑
I have severe pain or discomfort ❑
I have extreme pain or discomfort ❑

**ANXIETY / DEPRESSION**

I am not anxious or depressed ❑
I am slightly anxious or depressed ❑
I am moderately anxious or depressed ❑
I am severely anxious or depressed ❑
I am extremely anxious or depressed ❑



*Canada (English) © 2009 EuroQol Group EQ-5D™ is a trade mark of the EuroQol Group*

- We would like to know how good or bad your health is TODAY.
- This scale is numbered from 0 to 100.
- 100 means the best health you can imagine.
  0 means the worst health you can imagine.
- Mark an X on the scale to indicate how your health is TODAY.
- Now, please write the number you marked on the scale in the box below.

YOUR HEALTH TODAY = [ ]

Date: ______________________________

The best health you can imagine

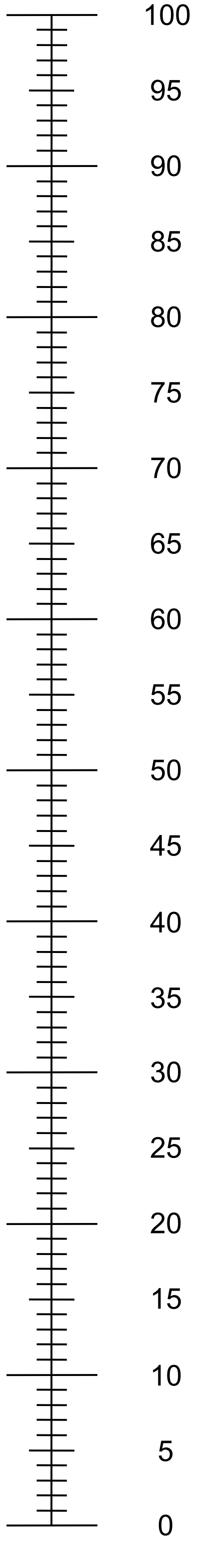


The worst health you can imagine



*Canada (English) © 2009 EuroQol Group EQ-5D™ is a trade mark of the EuroQol Group*

# Annexe 6

# Modified Barthel Index of Activities of Daily Living (mBI)

# Modified Barthel Index

## Grille d'évaluation

No sujet ______________________ Date d'administration ______________________

| | 1<br>Incapable | 2<br>Tentatives non-sécuritaires | 3<br>Réussit avec aide modérée | 4<br>Réussit avec aide minimale | 5<br>Autonome |
|---|---|---|---|---|---|
| Hygiène du visage (dents, barbe) | 0 | 1 | 3 | 4 | 5 |
| Prendre son bain | 0 | 1 | 3 | 4 | 5 |
| Aller à la toilette | 0 | 2 | 5 | 8 | 10 |
| Continence urinaire | 0 | 2 | 5 | 8 | 10 |
| Continence fécale | 0 | 2 | 5 | 8 | 10 |
| S'alimenter | 0 | 2 | 5 | 8 | 10 |
| S'habiller | 0 | 2 | 5 | 8 | 10 |
| Monter des escaliers | 0 | 2 | 5 | 8 | 10 |
| Marcher | 0 | 3 | 8 | 12 | 15 |
| Se déplacer en chaise roulante (si incapable de marcher) | 0 | 1 | 3 | 4 | 5 |
| Transférer au lit | 0 | 3 | 8 | 12 | 15 |

**Total : ________ / 100**

# Annexe 7

# Glasgow Outcome Scale – Extended (GOSE)

# Entretien structuré pour l'Échelle de devenir de Glasgow étendue (GOSE)

Nom : Prénom : Date de l'examen :

Date de naissance : ( ans) Date de l'accident : Sexe : ○ M ○ F

Age lors de l'accident : Délai depuis l'accident :

Interlocuteur : Patient seul :___; Parent/Ami/Référent seul : ___; Patient + Parent/Ami/ Référent:___

Examinateur : Résultat :

CONSCIENCE

1 Le sujet est-il capable de répondre aux ordres simples ou de dire un mot ? ○ Oui ○ Non
(4)

INDEPENDANCE AU DOMICILE

2a Le sujet est-il capable de se prendre en charge à domicile, sans aide, pendant 24 H. si nécessaire ? ○ Oui ○ Non

Pour répondre "Oui" le sujet doit être capable de se prendre en charge dans les différentes activités de la vie quotidienne pendant 24 H. si nécessaire, même si en ce moment ce n'est pas le cas.
Ceci comprend la capacité à planifier et à mener à bien les activités suivantes : toilette, habillage, préparation d'un repas pour soi-même, réponse au téléphone et résolution des problèmes domestiques mineurs.
Le sujet doit être capable d'exécuter ces activités sans incitation ni rappel et peut être laissé seul durant la nuit.
Si vous avez répondu "Oui", ne répondez pas à la question 2b.

2b Le sujet est-il capable de se prendre en charge à domicile, sans aide, pendant 8 H. si nécessaire ? ○ Oui ○ Non
(3.1) (3.2)

Pour répondre "Oui" le sujet doit être capable de se prendre en charge dans les différentes activités de la vie quotidienne pendant au moins 8 H durant la journée si nécessaire, même si en ce moment ce n'est pas le cas.

2c Avant le traumatisme le sujet était-il capable de se prendre en charge ? ○ Oui ○ Non

INDEPENDANCE EN DEHORS DU DOMICILE

3a Le sujet est-il capable de faire ses courses sans aide ? ○ Oui ○ Non
(3.1)

Ceci inclut la capacité de planifier les achats, de les payer, et de se comporter de façon appropriée en public.
Si le sujet ne fait pas habituellement ses courses, répondre à la question comme s'il avait à les faire.

3b Avant le traumatisme le sujet était-il capable de faire ses courses sans aide ? ○ Oui ○ Non

4a Le sujet est-il capable de se déplacer dans la localité sans aide ? ○ Oui ○ Non
(3.1)

Le sujet peut conduire, utiliser les transports en commun ou prendre un taxi (le commander par téléphone, donner les instructions au chauffeur).

4b Avant le traumatisme, le sujet était-il capable de se déplacer dans la localité sans aide ? ○ Oui ○ Non

Page 1 sur 3

Entretien structuré pour l'échelle de devenir de Glasgow étendue (GOSE). Copyright Patrick Fayol - 2004
d'après Structured interviews for the GOS and GOSE Lindlay Wilson, Laura Pettigrew, Graham Teasdale 1998.

## Entretien structuré pour l'Échelle de devenir de Glasgow étendue (GOSE)

TRAVAIL

5a Le sujet est-il capable actuellement de travailler à son niveau antérieur ? ○ Oui ○ Non

Si le sujet était à la recherche d'un travail avant le traumatisme, répondre "Oui" si le traumatisme n'a pas affecté ses chances d'obtenir un travail ou le niveau de travail qu'il pouvait espérer.
Si le sujet était étudiant avant le traumatisme, répondre "Oui" si sa capacité à suivre des études n'a pas été affectée.
Si vous avez répondu "Oui", ne répondez pas à la question 5b.

5b Importance de la restriction du travail : 1) réduction de la capacité de travail
2) uniquement capable de travailler en milieu protégé ou dans un travail non compétitif ou incapable de travailler
○ 1 (2.1) ○ 2 (2.2)

5c Avant le traumatisme, le sujet avait-il un travail ou était-il à la recherche d'un emploi (répondre "Oui") ou ni l'un ni l'autre (répondre "Non") ? ○ Oui ○ Non

VIE SOCIALE ET LOISIRS

6a Le sujet est-il capable de poursuivre des relations sociales régulières et d'avoir des activités de loisirs en dehors du domicile ? ○ Oui ○ Non

Si le sujet n'a pas repris toutes ses activités de loisirs, mais qu'il n'est pas gêné par des difficultés physiques ou mentales, répondre "Oui".
S'il a arrêté ses activités en raison d'un manque d'intérêt ou de motivation répondre "Non".
Si vous avez répondu "Oui", ne répondez pas à la question 6b.

6b Importance de la restriction des activités sociales ou de loisirs :
par rapport à avant le traumatisme
1) participe un peu moins : moins de deux fois moins souvent
2) participe beaucoup moins : plus de deux fois moins souvent
3) participe rarement, pour ne pas dire jamais
○ 1 (1.2) ○ 2 (2.1) ○ 3 (2.2)

6c Avant le traumatisme le sujet prenait-il part à des activités sociales régulières et à des activités de loisirs en dehors du domicile ? ○ Oui ○ Non

FAMILLE ET AMIS

7a Y-a-t-il eu des problèmes psychologiques ayant conduit à des ruptures familiales ou amicales toujours présentes actuellement ? ○ Oui ○ Non

Les modifications typiques de la personnalité post-traumatique sont : l'impulsivité, l'irritabilité, l'anxiété, l'indifférence à autrui, les changements d'humeur, la dépression et le comportement irresponsable ou enfantin.
Si vous avez répondu "Non", ne répondez pas à la question 7b.

7b Importance des ruptures ou des tensions :
1) occasionnelles : moins d'une fois par semaine
2) fréquentes : une fois par semaine ou plus mais tolérable
3) constantes : quotidiennes et intolérables.
○ 1 (1.2) ○ 2 (2.1) ○ 3 (2.2)

7c Avant le traumatisme y avait-il des problèmes avec la famille ou les amis ? Si tel était le cas mais si ces problèmes se sont aggravés de façon marquée, répondre quand même "Non". ○ Oui ○ Non

REPRISE D'UNE VIE NORMALE

8a Existe-t-il actuellement d'autres problèmes dus au traumatisme et qui affectent la vie quotidienne ? ○ Oui (1.2) ○ Non (1.1)

Les autres problèmes typiquement rapportés après un traumatisme crânien sont : céphalées, vertiges, fatigue, sensibilité au bruit ou à la lumière, ralentissement, manque de mémoire et difficultés de concentration.

8b Existait-il des problèmes du même ordre avant le traumatisme ? Si tel était le cas mais si ces problèmes se sont aggravés de façon marquée, répondre quand même "Non". ○ Oui ○ Non

Nom : Date de l'examen : GOSE : Page 2 sur 3

Entretien structuré pour l'échelle de devenir de Glasgow étendue (GOSE). Copyright Patrick Fayol - 2004
d'après Structured interviews for the GOS and GOSE Lindlay Wilson, Laura Pettigrew, Graham Teasdale 1998.

## Entretien structuré pour l'Échelle de devenir de Glasgow étendue (GOSE)

Épilepsie : Depuis le traumatisme le sujet a-t-il présenté des crises d'épilepsie ? oui non
Lui a-t-on dit qu'il risquait de faire des crises d'épilepsie ? oui non

Quel est le facteur le plus important du devenir ?
le traumatisme crânien : ____ un traumatisme non crânien ou une maladie : ____ un mélange des deux : ____

Cotation : le score global du patient est basé sur l'indication de la catégorie de devenir la moins bonne. Reportez-vous aux recommandations pour de plus amples informations concernant la passation de l'entretien et la cotation.

Bonne récupération :
Niveau supérieur .................................. 1.1
Niveau inférieur .................................. 1.2

Handicap modéré :
Niveau supérieur ................................ 2.1
Niveau inférieur ................................. 2.2

Handicap sévère :
Niveau supérieur ................................ 3.1
Niveau inférieur ................................. 3.2

État végétatif persistant ........................................ 4

(Décès) .................................................................. 5



Entretien structuré pour l'échelle de devenir de Glasgow étendue (GOSE). Copyright Patrick Fayol - 2004
d'après Structured interviews for the GOS and GOSE Lindlay Wilson, Laura Pettigrew, Graham Teasdale 1998.

# Annexe 8

# Questionnaire de suivi

# Questionnaire de suivi

No sujet ________________________________

| | |
|---|---|
| Résolution du symptôme initial | ☐ Non<br>☐ Partiellement<br>☐ Complètement |
| Usage de stéroïdes depuis la dernière visite | ☐ Non<br>☐ Oui : dose + dates : _____________ |
| Usage d'antiplaquettaires depuis la dernière visite | ☐ Non<br>☐ Oui : doses + dates : ____________ |
| Score MMSE | ____________________________________ |
| Score mBI | ____________________________________ |
| Score EQ-5D (détaillé + VAS) | ____________________________________ |
| Score GOSE (uniquement pour le suivi final) | ____________________________________ |

## Surveillance évènements indésirables

| | |
|---|---|
| Draînage chirurgical depuis la dernière visite | ☐ Non<br>☐ Oui, planifié, date :_______________<br>☐ Oui, non-planifié, date :___________ |
| Thrombophlébite diagnostiquée depuis la dernière visite | ☐ Non<br>☐ Oui, date : ______________________ |
| Embolie pulmonaire diagnostiquée depuis la dernière visite | ☐ Non<br>☐ Oui, date : ______________________ |
| ICT diagnostiquée depuis la dernière visite | ☐ Non<br>☐ Oui, date : ______________________ |
| AVC diagnostiquée depuis la dernière visite | ☐ Non<br>☐ Oui, date : ______________________ |
| Pseudo-néphrolithiase diagnostiquée depuis la dernière visite | ☐ Non<br>☐ Oui, date : ______________________ |
| Changement de la vision depuis la dernière visite | ☐ Non<br>☐ Oui, date et détails : _____________ |
| Autre complication potentiellement attribuable à l'administration d'acide tranexamique (et date) | ____________________________________ |

Signature ________________________________ Date ________________________________

# Annexe 9

# Monographie de l'acide tranexamique

# MONOGRAPHIE

## $^{Pr}$CYKLOKAPRON*

Comprimés d'acide tranexamique à 500 mg, BP
et solution d'acide tranexamique à 100 mg/mL pour injection, BP

Agent antifibrinolytique

Pfizer Canada inc.
17300, autoroute Transcanadienne
Kirkland (Québec)  H9J 2M5

Date de préparation :
13 juin 2013

N$^{o}$ de contrôle : 163228

* M.D. de Pfizer Health AB
Pfizer Canada inc., licencié
© Pfizer Canada inc. 2013

# MONOGRAPHIE

## PrCYKLOKAPRON*

**Comprimés d'acide tranexamique, BP**
**et solution d'acide tranexamique pour injection, BP**

## CLASSE THÉRAPEUTIQUE

Agent antifibrinolytique

## ACTION

CYKLOKAPRON (acide tranexamique) exerce un effet antifibrinolytique en inhibant par compétition l'activation du plasminogène en plasmine. C'est aussi un faible inhibiteur non compétitif de la plasmine. Ces propriétés permettent à CYKLOKAPRON d'être utilisé cliniquement comme agent antifibrinolytique dans le traitement d'hémorragies fibrinolytiques systémiques ou locales. In vitro, son mécanisme d'action est similaire à celui de l'acide E aminocaproïque (AEAC), mais environ 10 fois plus puissant.

Chez l'humain, son absorption dans le tube digestif est incomplète (40 %).

L'acide tranexamique se lie beaucoup plus fortement aux sites faibles et forts de la molécule de plasminogène que l'AEAC. Le rapport de fixation correspond à la différence de puissance des deux composés. La signification pharmacologique de la liaison à ces différents sites n'a pas encore été évaluée.

L'acide tranexamique ne se fixe pas à la sérum-albumine. Le taux de liaison aux protéines plasmatiques semble négligeable comparé aux taux plasmatiques thérapeutiques de 5 à 10 mg/L et semble être entièrement expliqué par la fixation au plasminogène.

Les voies possibles de biotransformation sont l'acétylation ou la désamination suivie de l'oxydation ou de la réduction. À la suite d'une administration orale, approximativement 50 % de la molécule mère, 2 % d'acide dicarboxylique désaminé, et 0,5 % du produit acétylé, sont excrétés.

L'acide tranexamique est éliminé par filtration glomérulaire. À la suite d'une administration intraveineuse de 10 mg par kg de poids corporel, 30 % du produit est excrété en une heure, 55 % en 3 heures et 90 % en 24 heures. À la suite d'une administration orale de 10 à 15 mg par kg de poids corporel, l'excrétion est de 1 % en une heure, 7 % en 3 heures et 39 % en 24 heures.

Une administration intraveineuse de 10 mg par kg de poids corporel donne des concentrations plasmatiques de 18,3 µg, 9,6 µg et 5 µg par mL, une heure, trois heures et cinq heures après l'injection.

Lorsque le médicament est administré 36 à 48 heures avant une intervention chirurgicale en 4 doses de 10 à 20 mg par kg de poids corporel, une concentration active antifibrinolytique (10 µg/mL) d'acide tranexamique demeure jusqu'à 17 heures dans les tissus examinés, et jusqu'à 7 à 8 heures dans le sérum (Andersson et coll., 1968).

L'acide tranexamique pénètre le placenta. Après une injection intraveineuse de 10 mg par kg, la concentration dans le sérum du fœtus peut s'élever à environ 30 µg par mL.

L'acide tranexamique se retrouve aussi dans le lait maternel durant la lactation, à des concentrations de 1/100 des taux sériques correspondants.

Après l'administration orale ou intraveineuse, l’acide tranexamique passe dans le sperme, et inhibe son activité fibrinolytique, sans toutefois affecter la motilité des spermatozoïdes (Liedholm, 1973).

La capacité que possède l’acide tranexamique de traverser la barrière hémato-encéphalique a été démontrée lors de son administration à des patients présentant des ruptures d’anévrisme intracrânien.

L’acide tranexamique se répand rapidement dans le liquide articulaire et dans la membrane synoviale. La concentration retrouvée dans le liquide articulaire est la même que celle retrouvée dans le sérum. La demi-vie biologique dans le liquide articulaire était d’environ trois heures.

Trois heures après une dose orale unique de 25 mg par kg de poids corporel, la concentration sérique maximale était de 15,4 mg par L, et le taux de l’humeur aqueuse était de 1,6 mg par L.

## INDICATIONS ET USAGE CLINIQUE

Œdème angioneurotique héréditaire.

Fibrinolyse locale accrue lorsque le diagnostic indique une hyperfibrinolyse comme dans les cas d’une conisation du col utérin, d’une extraction dentaire chez les patients présentant des troubles de la coagulation (conjointement avec le facteur antihémophilique), d’une épistaxis, d’un hyphéma, et d’une ménorragie (hyperménorrhée).

## CONTRE-INDICATIONS

**CYKLOKAPRON** (acide tranexamique) ne doit pas être administré à des patients présentant des antécédents ou un risque de thrombose, à moins qu'un traitement aux anticoagulants puisse être administré simultanément. La préparation ne doit pas être administrée à des patients présentant des troubles de vision des couleurs acquis. Si le patient se plaint de troubles visuels durant le traitement, l'administration de la préparation doit être interrompue.

CYKLOKAPRON ne doit pas être administré aux patients atteints de troubles thromboemboliques actifs, tels qu'une thrombose veineuse profonde, une embolie pulmonaire ou une thrombose cérébrale.

On ne doit pas administrer l'acide tranexamique aux patients qui souffrent d'hémorragies sous-arachnoïdiennes : l'expérience médicale limitée démontre que la diminution des risques de nouveaux saignements s'accompagne d'une augmentation du taux d'ischémie cérébrale.

Hématurie (*voir les rubriques* MISES EN GARDE *et* PRÉCAUTIONS)

On n'administrera pas non plus l'acide tranexamique aux patients atteints d'une hypersensibilité à l'acide tranexamique ou à tout autre ingrédient de ce produit.

## MISES EN GARDE

Des cas de troubles visuels, y compris de déficience visuelle, de vision floue et de perturbation de la perception des couleurs, ont été signalés en association avec l'acide tranexamique. Un examen ophtalmique (acuité visuelle, vision des couleurs, fond de l'œil, champ de vision, etc.) est recommandé chez les patients qui doivent être traités avec l'acide tranexamique durant plusieurs semaines, si possible avant de commencer le traitement, et à intervalles réguliers durant le traitement.

Les patientes qui ont des menstruations irrégulières ne devraient pas utiliser CYKLOKAPRON avant d'avoir établi la cause de cette irrégularité.

Les patientes doivent consulter leur médecin si les saignements menstruels n'ont pas diminué après trois cycles menstruels.

Lorsque CYKLOKAPRON ne parvient pas efficacement à réduire les saignements menstruels, on doit envisager un traitement de rechange.

Des cas de thrombose ou de thromboembolie veineuse ou artérielle ont été signalés chez des patients traités par le CYKLOKAPRON. Les patientes à risque élevé de thrombose (événement thromboembolique préalable et antécédents familiaux de troubles thromboemboliques) ne devraient utiliser CYCKLOKAPRON que s'il est fortement indiqué et sous étroite surveillance médicale.

Dans le cas des patients souffrant de coagulation intravasculaire disséminée (CID), qui doivent prendre CYKLOKAPRON, la surveillance étroite d'un médecin qualifié dans le traitement de ce trouble est nécessaire.

Le traitement à l'acide tranexamique n'est pas indiqué dans l'hématurie due aux maladies du parenchyme rénal. Une précipitation intravasculaire de fibrine se produit fréquemment dans ces conditions et peut aggraver la maladie. De plus, dans les cas d'une hémorragie rénale massive de quelque cause que ce soit, le traitement antifibrinolytique peut causer la rétention d'un caillot dans le bassinet du rein.

Des convulsions ont été signalées en lien avec le traitement par l'acide tranexamique.

Contraception hormonale : On sait que les contraceptifs hormonaux combinés accroissent le risque de thromboembolie veineuse, de même que le risque de thrombose artérielle (accident vasculaire cérébral, infarctus du myocarde). CYKLOKAPRON étant un antifibrinolytique, il peut accroître ce risque encore davantage si on le prescrit à une femme qui prend déjà un contraceptif hormonal. Il ne faut donc le prescrire aux femmes qui prennent un contraceptif hormonal qu'en cas d'extrême nécessité et seulement si les bienfaits éventuels de CYKLOKAPRON l'emportent sur l'augmentation du risque thrombotique (*voir la rubrique* Interactions médicamenteuses).

Patients prenant des anticoagulants (*voir la rubrique* POSOLOGIE ET MODE D'ADMINISTRATION)

Usage durant la grossesse

L'innocuité de CYKLOKAPRON (acide tranexamique) durant la grossesse n'a pas encore été établie. Aucun effet nocif n'a été rapporté jusqu'à maintenant.

Une femme présentant une hémorragie fibrinolytique dans son quatrième mois de grossesse a été traitée avec l'acide tranexamique pendant une durée de 64 jours. La dose totale a été de 256 g. L'accouchement se fit spontanément durant la 30e semaine de grossesse et fut normal à tous points de vue. Le bébé était en bonne santé.

Un autre cas révèle qu'une patiente qui avait déjà perdu deux enfants à la suite d'un décollement prématuré du placenta et qui était menacée d'une troisième fausse couche, fut traitée avec l'acide tranexamique. En effet, l'hémorragie se déclencha durant la 26$^{e}$ semaine de sa troisième grossesse, indiquant un autre décollement du placenta. Le diagnostic révéla une protéolyse pathologique due à une activation prédominante du système fibrinolytique. Entre la 26$^{e}$ et la 33$^{e}$ semaine de grossesse, on lui administra environ 250 g d'acide tranexamique par voie intraveineuse et orale. L'hémorragie s'arrêta et elle accoucha, par césarienne, d'un bébé bien portant.

L'acide tranexamique passe dans le fœtus (Kullander et Nilsson, 1970). Une injection intraveineuse de 10 mg/kg peut mener à une concentration d'environ 30 µg/mL dans le sérum du fœtus. L'activité fibrinolytique est très élevée chez les nouveau-nés. Toutefois, nous ne savons pas d'une façon certaine si une réduction de cette activité durant les premières heures de vie est nuisible. Kullander et Nilsson, qui possèdent une vaste expérience avec l'utilisation de l'acide tranexamique dans les cas d'accouchement, n'ont observé aucun effet négatif chez le nourrisson.

## PRÉCAUTIONS

Un soin particulier doit être pris dans les cas d'insuffisance rénale à cause d'un risque d'accumulation, d'une hématurie prononcée dans le haut appareil urinaire, car quelques cas isolés d'obstruction du passage ont été observés dans l'appareil urinaire (*voir la rubrique* POSOLOGIE ET MODE D'ADMINISTRATION).

Les patients suivants doivent consulter leur médecin avant de commencer un traitement par CYKLOKAPRON : patients obèses et diabétiques, patientes ayant un syndrome des ovaires polykystiques ou des antécédents familiaux (parente du premier degré) de cancer de l'endomètre, femmes prenant un estrogène seul (sans progestatif) ou du tamoxifène.

Mères allaitant :
L'acide tranexamique est sécrété dans le lait maternel à une concentration de 1/100 seulement des taux sériques correspondants (Eriksson *et coll.*, 1971). Les investigateurs sont d'avis que l'acide tranexamique peut être administré aux mères qui allaitent sans risque pour l'enfant.

Enfants :
On ne possède pas d'expérience clinique avec CYKLOKAPRON chez les enfants ménorragiques de moins de 18 ans.

Conduite automobile et utilisation de machines :
L'acide tranexamique peut causer des étourdissements et, par conséquent, avoir une influence sur l'aptitude à conduire un véhicule ou à utiliser une machine.

Interactions médicamenteuses :
Les interactions entre CYKLOKAPRON et d'autres médicaments n'ont fait l'objet d'aucune étude. En raison de l'absence d'études à ce sujet, le traitement simultané avec des anticoagulants se fera sous l'étroite surveillance d'un médecin qualifié dans ce domaine.

Interactions médicamenteuses possibles pouvant se solder par un infarctus du myocarde : contraceptifs hormonaux, hydrochlorothiazide, desmopressine, sulbactame-ampicilline, carbazochrome, ranitidine, nitroglycérine.
CYKLOKAPRON étant un antifibrinolytique, il peut accroître le risque thrombotique associé à la contraception hormonale combinée (*voir la rubrique* MISES EN GARDE).

## RÉACTIONS INDÉSIRABLES

**Troubles gastro-intestinaux :** Des symptômes gastro-intestinaux (nausées, vomissements, diarrhée) peuvent se produire, mais disparaissent lorsque la dose est réduite.
**Troubles du système nerveux :** Des cas isolés d'étourdissements et d'hypotension artérielle ont été rapportés.
**Troubles du système immunitaire :**On a signalé des cas moins fréquents de réactions allergiques cutanées.

**Troubles oculaires**
Dans les études expérimentales chez l'animal, il faut retenir que : chez le chien, des fortes doses d'acide tranexamique administrées à long terme, et chez le chat, des injections intraveineuses de 250 mg/kg de poids corporel par jour durant 14 jours ont produit des changements rétiniens. Chez le rat, de tels changements ne se sont pas produits après l'administration de la dose maximale tolérée.

Lors de l'examen ophtalmique, aucun changement rétinien n'a été rapporté ou observé chez les patients traités par CYKLOKAPRON durant plusieurs semaines ou plusieurs mois.

**Surveillance après la mise sur le marché** :
De rares cas de réactions indésirables ont été signalés avec l'administration d'acide tranexamique.
**Troubles vasculaires :** événements thromboemboliques (infarctus aigu du myocarde, thrombose, thrombose artérielle d'un membre, thrombose de la carotide, infarctus cérébral, accident vasculaire cérébral, thrombose veineuse profonde, embolie pulmonaire, thrombose cérébrale, nécrose aiguë du cortex rénal et occlusion de l'artère et de la veine

centrales de la rétine). L'injection rapide de CYKLOKAPRON peut entraîner de l'hypotension artérielle.

**Troubles oculaires :** déficience visuelle, vision floue, perturbation de la perception des couleurs (chromatopsie).

**Troubles du système nerveux :** étourdissements et crises convulsives.

## SYMPTÔMES ET TRAITEMENT DU SURDOSAGE

On ne connaît aucun cas de surdosage par CYKLOKAPRON chez l'humain. Les symptômes peuvent être des nausées, de la diarrhée, des étourdissements, des céphalées, des convulsions, des vomissements, des symptômes orthostatiques et de l'hypotension. Le traitement du surdosage consisterait à provoquer des vomissements, effectuer un lavage gastrique, administrer un traitement au charbon de bois et administrer un traitement symptomatique. On s'assurera que la diurèse est adéquate.

On a observé une légère intoxication après l'administration d'une dose de 37 g d'acide tranexamique chez une jeune fille de dix-sept ans qui avait subi un lavage gastrique.

**En cas de surdosage soupçonné, communiquez avec le centre antipoison de votre région.**

## POSOLOGIE ET MODE D'ADMINISTRATION

Conisation du col utérin : 2 à 3 comprimés toutes les 8 à 12 heures durant les 12 jours de la période postopératoire.

Épistaxis : 2 à 3 comprimés toutes les 8 à 12 heures durant 10 jours.

Hyphéma : 2 à 3 comprimés toutes les 8 à 12 heures durant 7 jours.

En chirurgie dentaire chez les patients souffrant de coagulopathie : 2 heures avant l'intervention, le facteur VIII et le facteur IX doivent être administrés conjointement avec CYKLOKAPRON, 25 mg par voie orale ou 10 mg par voie intraveineuse par kg de poids corporel. Après l'intervention, 25 mg/kg de CYKLOKAPRON sont administrés par voie orale 3 à 4 fois par jour durant 6 à 8 jours. Après l'intervention chirurgicale, le patient n'a généralement plus besoin de traitement substitutif.

***Administration***

La solution de CYKLOKAPRON pour injection doit être administrée par voie intraveineuse (injection ou perfusion). Son administration doit se faire lentement pendant au moins 5 minutes. Le débit recommandé d'administration en bolus est de 50 mg/min. Pour administrer directement 50 mg/min de solution en injection, il faut administrer 0,5 mL/min de solution de CYKLOKAPRON non diluée (100 mg/mL) par injection intraveineuse lente. Pour administrer 50 mg/min en perfusion, on peut administrer des solutions diluées avec 1 % d'acide tranexamique (c.-à-d. 1 g par 100 mL ou 10 mg/mL) à raison de 5 mL/min ou des solutions diluées avec 2 % d'acide tranexamique à raison de 2,5 mL/min.

Pour une perfusion intraveineuse, la solution de CYKLOKAPRON pour injection peut être mélangée à :

- une solution d'électrolytes (p. ex., solution de chlorure de sodium à 0,9 %, solution de Ringer);
- une solution glucidique (p. ex., solution de glucose à 5 %);
- une solution d'acides aminés;
- une solution de dextran (p. ex., dextran 40, dextran 70).

On peut ajouter de l'héparine à la solution de CYKLOKAPRON pour injection. La solution de CYKLOKAPRON pour injection ne devrait pas être mélangée avec le sang et des solutions pour perfusion contenant de la pénicilline.

Le volume nécessaire de solution de CYKLOKAPRON pour injection peut être ajouté à une solution pour perfusion afin d'atteindre une concentration finale de 1 ou 2 g par 100 mL (10 ou 20 mg/mL, 1 % ou 2 %). Le tableau ci-dessous présente les proportions à employer pour obtenir un volume total de 100 mL :

| **Préparation de solutions pour perfusion** | **Solution à 1 % (10 mg/mL)** | **Solution à 2 % (20 mg/mL)** |
|---|---|---|
| **Acide tranexamique (g)** | **1 g** | **2 g** |
| **Diluants compatibles*** | **q.s.p. 100 mL** | **q.s.p. 100 mL** |

* Voir plus haut pour connaître les diluants compatibles.

Remarque : On obtient **1 g d'acide tranexamique** à partir d'une fiole de 10 mL ou de 2 fioles de 5 mL; on obtient **2 g d'acide tranexamique** à partir de 2 fioles de 10 mL ou de 4 fioles de 5 mL.

Le tableau suivant présente un exemple de préparation et d'administration d'une solution pour perfusion intraveineuse :

| **Débits de perfusion pour les solutions d'acide tranexamique diluées et non diluées** | | | | |
|---|---|---|---|---|
| | | **Bolus (50 mg/min)** | | |
| | **Poids (kg)** | **Solution non diluée (100 mg/mL)** | **Solution diluée** | |
| | | | **1 % (10 mg/mL)** | **2 % (20 mg/mL)** |
| **Débit de perfusion** | - | 0,5 mL/min | 5 mL/min | 2,5 mL/min |
| Exemple pour un patient recevant 10 mg/kg | 70 | 7 mL (14 min) | 70 mL (14 min) | 35 mL (14 min) |

Le mélange doit être utilisé immédiatement après la préparation. S'il y a lieu, le mélange doit être conservé à une température de 2 à 8 °C pendant 24 heures au maximum. Si le mélange n'est pas utilisé dans les 24 heures suivant sa préparation, il faut le jeter.

Les ampoules de CYKLOKAPRON pour injection sont stériles. CYKLOKAPRON pour injection est destiné à un usage unique. Il faut jeter le produit non utilisé. Comme avec tout autre produit parentéral, il faut examiner la solution de CYKLOKAPRON pour

injection avant de l’administrer, si le liquide et le contenant le permettent, afin de s’assurer de sa transparence et de l'absence de particules, de précipité, de changement de couleur ou de fuite.

Ménorragie (hyperménorrhée) :
De 2 à 3 comprimés, 3 à 4 fois par jour durant plusieurs jours. Le traitement par CYKLOKAPRON ne doit être mis en route que lorsque des saignements abondants ont débuté.

Œdème angioneurotique héréditaire :
Certains patients reconnaissent le début des crises et peuvent être traités par intermittence à raison de 2 à 3 comprimés, 2 à 3 fois par jour durant plusieurs jours. D’autres doivent être traités continuellement avec cette dose.

Enfants :
La posologie doit être calculée en fonction du poids corporel à raison de 25 mg/kg, 2 à 3 fois par jour. On ne possède pas d’expérience clinique avec CYKLOKAPRON chez les enfants ménorragiques (hyperménorrhée) de moins de 18 ans.

Patients avec une fonction rénale altérée :
Chez les patients dont les concentrations sériques de créatine varient de 120 à 250 µmol/L, la dose recommandée est de 15 mg par voie orale ou 10 mg par voie intraveineuse d’acide tranexamique par kg de poids corporel 2 fois par jour; à des concentrations sériques de créatine de 250 à 500 µmol/L, la dose recommandée est de 15 mg par voie orale ou 10 mg par voie intraveineuse d’acide tranexamique par kg de poids corporel à un intervalle de 24 heures; à des concentrations sériques de créatine de 500 µmol/L ou plus, la même posologie peut être prescrite avec un intervalle de 48 heures entre les doses.

**Dose oubliée**

Si vous avez oublié de prendre un comprimé, prenez la prochaine dose, comme d'habitude. Ne prenez pas une double dose pour remplacer une dose oubliée.

**RENSEIGNEMENTS PHARMACEUTIQUES**

**Substance médicamenteuse :** Acide tranexamique

**Dénomination commune internationale :** Acide tranexamique, BP

**Dénomination chimique :** Acide *trans*-4-(aminométhyl)cyclohexane-1-carboxylique

**Formule développée :**

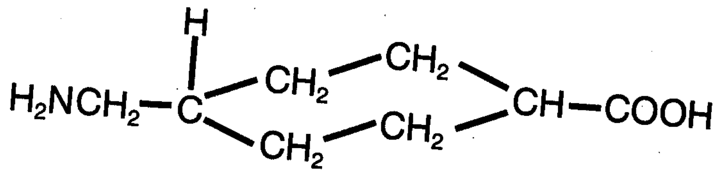


**Formule moléculaire :** $C_8H_{15}NO_2$

**Masse moléculaire :** 157,2

**Présentation :** Poudre cristalline blanche inodore ou presque inodore

**Solubilité :** L'acide tranexamique est soluble dans l'eau.

## COMPOSITION

**Comprimés (500 mg)**

Acide tranexamique, BP, 500 mg/comprimé

Ingrédients non médicinaux : cellulose microcristalline, silice colloïdal anhydre, hydroxypropylcellulose, stéarate de magnésium, polyvidone, talc. Les comprimés sont enduits d'une couche composée d'Eudragit, de stéarate de magnésium, de PEG 8000, de talc et de dioxyde de titane.

**Injection (100 mg/mL)**

Acide tranexamique, BP 100 mg/mL

Ingrédient non médicinal : **e**au pour injection à 1,0 mL

**pH :** CYKLOKAPRON pour injection a un pH de 6,5 à 8.

**Conservation :** Garder toutes les formes posologiques à une température ambiante allant de 15 à 30 °C.

## PRÉSENTATION

Comprimés (blancs, pelliculés, en forme de capsule, portant les lettres CY gravées entre 2 crochets horizontaux, 8 x 18 mm) en flacons de 100 comprimés. Chaque comprimé contient 500 mg d'acide tranexamique.

Solution pour injection : ampoules contenant 100 mg d'acide tranexamique par mL. Emballages de 10 ampoules de 5 mL et de 10 mL.

## PHARMACOLOGIE

La concentration plasmatique thérapeutique de CYKLOKAPRON (acide tranexamique) est de 5 à 15 mg/L. L'interaction fonctionnelle entre le plasminogène et l'activateur tissulaire, localisée principalement sur la fibrine, est empêchée par la dissociation du complexe entre la fibrine et les sites de liaison spécifiques du substrat sur le plasminogène. Un effet potentialisateur sur les inhibiteurs naturels semble aussi contribuer à l'effet clinique durant la thérapie antifibrinolytique.

### Effet antifibrinolytique in vivo

Trente minutes après avoir produit des états hyperfibrinolytiques chez des lapins par une injection de streptokinase, on a immédiatement arrêté la fibrinolyse par une injection intraveineuse de 30 mg/kg d'acide tranexamique, comparativement à une dose de 3 à 10 fois plus élevée de 100 à 300 mg/kg d'acide E aminocaproïque nécessaire pour obtenir des effets comparables.

Chez les chiens, une baisse immédiate de 40 % dans l'excrétion d'urokinase urinaire s’est produite à la suite d'une dose orale de 55 mg/kg d'acide tranexamique ajoutée à leur ration.

Une dose intraveineuse de 50 mg/kg d'acide tranexamique diminue la fibrinolyse pulmonaire chez le rat. L'inhibition maximale s’est produite de 5 à 15 minutes après une injection de 600 mg/kg et l'effet a duré 8 heures.

Influence sur d'autres mécanismes enzymatiques

L'acide tranexamique (5 x $10^{-2}$M) inhibe compétitivement l'activation de la trypsinogène par l'entérokinase, et inhibe non compétitivement l'activité protéolytique de la trypsine à une concentration 4 fois supérieure (p. ex., Dubber *et coll.*, 1965). Alors que l'acide aminocaproïque inhibe modérément la trypsine (40 %), la kallikréine urinaire (30 %) et la kallikréine pancréatique (60 %), l'acide tranexamique a peu d'effet (moins de 10 %) sur chacun de ces enzymes.

Un effet encore plus faible est exercé sur la thrombine (7 x $10^{-3}$M, 100 mg/L) (Andersson *et coll.*, 1965). L'acide tranexamique (7 x $10^{-2}$M) ajouté au sang, n'a pas d'influence sur la numération des plaquettes, sur le temps de coagulation, sur la méthode de Quick, ou sur le temps de Howell. Les taux plasmatiques du facteur AH, du facteur IX, de la prothrombine, du facteur VII, du facteur V et de la fibrinogène demeurent inchangés in vitro.

L'acide tranexamique (7 x $10^{-3}$M, 1 g/L) n'augmente pas l'agrégation plaquettaire chez l'humain in vitro. Au contraire, in vivo chez le chien, une dose de 30 mg/kg i.v. a démontré une diminution de l'agrégabilité induite par l'ADP et un effet stabilisateur sur l'adhésivité aux billes de verre (test de rétention des plaquettes) durant 24 heures après l'administration (Jong, 1974).

L'activité de la chymotrypsine n'est pas diminuée par l'action des antifibrinolytiques synthétiques, et une inhibition de l'action de la pepsine est observée seulement à hautes concentrations, 6 x $10^{-3}$M. La dégradation de la bradykinine dans le plasma humain n'est pas inhibée significativement à $10^{-2}$M.

Interaction avec le système cardiovasculaire :

L'acide tranexamique administré par perfusion i.v., chez le chat anesthésié, à raison de 0,4 à 2 mg/kg/min durant 60 minutes, et administré par voie i.m. chez le lapin, le chat et le chien à raison de 170 mg/kg, ne produit pas de changement significatif de la pression artérielle, de la respiration ou de l'ECG (Marmo *et coll.*, 1973).

Le mécanisme d'action de l'acide tranexamique sur le système cardiovasculaire est moins évident que celui de l'acide E aminocaproïque qui semble produire un effet sympathomimétique indirect. En relation avec son effet thérapeutique, l'acide tranexamique exerce un effet 10 fois moins puissant que celui de l'acide E aminocaproïque sur la pression artérielle. Les doses-seuil pour augmenter la pression artérielle et le pouls chez les chats anesthésiés sont de 50 à 100 mg/kg pour l'acide tranexamique, et de 30 à 50 mg/kg pour l'acide E aminocaproïque.

## TOXICITÉ

Toxicité aiguë :

La toxicité aiguë de l'acide tranexamique a été étudiée durant 24 heures et 7 jours chez les souris et les rats, et pendant 24 heures, 48 heures et 7 jours chez les lapins, suite à des administrations intraveineuses, intrapéritonéales, sous-cutanées et orales. Dans une quatrième étude, le taux de mortalité a été examiné 72 heures après une administration intraveineuse, orale et sous-cutanée. Les doses orales mortelles dépassent 5 à 10 g/kg de poids corporel chez toutes les espèces étudiées, et les valeurs de la $DL_{50}$ après une injection intraveineuse ont été d'environ 1 à 1,5 g/kg de poids corporel chez les souris, les rats et les chiens.

Toxicité subaiguë :

Dans six études de toxicité subaiguë, des doses quotidiennes d'acide tranexamique furent administrées : par voie orale à des rats (1 à 5 g/kg durant 10 semaines) et à des chiens (100 à 500 mg/kg durant 4 mois); par voie intraveineuse à des chiens (20 à 500 mg/kg durant 1 mois et 1 g/kg durant 3 jours) et à des lapins (60 à 180 mg/kg durant 13 jours); par voie intrapéritonéale à des rats (0 à 1 000 mg/kg pendant 2 semaines). Les seuls effets secondaires observés dans les études orales et intrapéritonéales, proportionnellement aux doses administrées, étaient des vomissements, des selles molles ou de la diarrhée, et une diminution du gain de poids.

Chez les lapins, le seul effet clinique retenu suite à l'administration intraveineuse a été de la tachypnée proportionnelle aux doses reçues.

L'étude à court terme (3 jours) de l'administration i.v. a révélé qu'un chien a vomi fréquemment durant la première perfusion, et à la fin de la perfusion, il est tombé en convulsion et il est mort. L'autopsie a démontré que ce chien avait fait une légère hémorragie cardiaque et l'examen histologique a révélé des pétéchies au coeur.

Dans l'étude d'un mois, les chiens ayant reçu une injection i.v. de 20, 100 ou 500 mg/kg/jour (Balazs & Porpora, 1969, Ohtake & Kepenis, 1969) ont présenté des vomissements et du ptyalisme aux deux doses les plus élevées. L'étude microscopique a révélé une thromboembolie pulmonaire chez un chien du groupe ayant reçu la dose intermédiaire, et chez un autre ayant reçu la dose plus élevée. Ce dernier chien avait également deux thrombophlébites dans la vessie. Aucune hémorragie cardiaque n'a été notée.

Toxicité chronique :

Dans huit études de toxicité chronique, l'acide tranexamique a été administré : par voie orale à des rats (0 à 4 000 mg/kg/jour durant un an), à des chiens (200 à 1 600 mg/kg/jour et 800 à 1 200 mg/kg/jour durant un an); dans leur régime alimentaire à des rats (0 à 4,8 % durant 22 mois et 0 à 5 % durant 19 mois) et à des rats et souris (4,8 % durant 20 mois); par voie sous-cutanée à des souris (0,8 à 1,5 mg en une seule injection) sous observation durant un an, et à des rats, par voie sous-cutanée (0 à 3,5 g/kg/semaine) ou par voie orale (0 à 10 g/kg/semaine) pendant deux ans.

À des niveaux de dosage extrêmement élevés de 2 x 400 mg/kg/jour et à des concentrations plasmatiques maximales d'environ 200 mg par litre, des études de toxicité chronique par voie orale chez les chiens ont démontré une atrophie de la rétine antérieure (*ora ciliniaris retinae*) et postérieure (autour du disque optique).

Les changements atrophiques de la rétine se sont avérés semblables à ceux observés chez l'homme et les chiens en sénescence. Chez l'homme, les concentrations plasmatiques maximales varient de 10 à 20 mg par litre à la suite d'une dose orale thérapeutique d'environ 30 mg par kg de poids corporel. Des changements similaires se sont produits chez des chiens et des chats à la suite d'une perfusion i.v. quotidienne élevée d'acide tranexamique.

L'étude à vie de l'addition au régime alimentaire de rats (étude de carcinogénicité) n'a démontré aucune lésion aux yeux.

Dans l'une des études de carcinogénicité dans laquelle de fortes doses d'acide tranexamique ont été administrées à des rats, l'hyperplasie biliaire, le cholangiome et l'adénome du foie furent rapportés.

Ces observations ne se sont pas répétées dans un certain nombre d'études subséquentes sur l'action carcinogène.

Une augmentation d'incidence des leucémies (bien que statistiquement insignifiante) a été rapportée dans une étude sur des souris ayant reçu 4,8 % d'acide tranexamique durant 20 mois. Dans d'autres études, la fréquence et l'apparence histologique des tumeurs examinées étaient similaires dans les groupes d’animaux traités et non traités.

## RÉFÉRENCES


Blohme G. Treatment of hereditary angioneurotic oedema with tranexamic acid. A random double-blind cross-over study. Acta Med Scand 1972; 192: 293-298.

Bramsen T. Traumatic hyphaema treated with the antifibrinolytic drug tranexamic acid. Acta Ophthalmol 1976; 54: 250-256.

Bramsen T. Traumatic hyphaema treated with the antifibrinolytic drug tranexamic acid. II. Acta Ophthalmol 1977; 55: 616-620.

Forbes CD, Barr RD, Reid G, Thompson C, Prentice DRM, McNicol DP, Douglas AS. Tranexamic acid in control of haemorrhage after dental extraction in haemophilia and Christmas disease. Br Med J 1972; 2: 311-313.

Jerndal T, Friesen M. Tranexamic acid (AMCA) and late hyphaema. A double-blind study in cataract surgery. Acta Ophthalmol 1976; 54: 417-429.

Landin L-E, Weiner E. Late bleeding after conization. The effect of tranexamic acid (Cyklokapron). Opusc Med 1975; 20: 280-284.

Nilsson L, Rybo G. Treatment of menorrhagia with an antifibrinolytic agent, tranexamic acid (AMCA). A double-blind investigation. Acta Obstet Gynecol 1967; 46: 572-580.

Nilsson L, Rybo G. Treatment of menorrhagia. Am J Obstet Gynecol 1971; 110: 713-720.

Petruson B. A double-blind study to evaluate the effect on epistaxis with oral administration of the antifibrinolytic drug tranexamic acid (Cyklokapron). Acta Oto-Laryngol 1974; Suppl. 317: 57-61.

Sheffer AL, Austen KF, Rosen FS. Tranexamic acid therapy in hereditary angioneurotic edema. N Engl J. Med 1972; 287: 452-454.

Sheffer AL, Fearon DT, Austen F, Rosen FS. Tranexamic acid: Pre-operative prophylactic therapy for patients with hereditary angioneurotic oedema. J Allerg Clin Immunol 1977; 60: 38-40.

Tavenner RWH. Use of tranexamic acid in control of teeth in haemophilia in Christmas disease. Br Med J 1972; 2: 314-315.

## PARTIE III : RENSEIGNEMENTS POUR LE CONSOMMATEUR

**PrCYKLOKAPRON***
**(acide tranexamique)**

**Le présent dépliant constitue la troisième et dernière partie de la « monographie de produit » publiée par suite de l'homologation de CYKLOKAPRON pour la vente au Canada, et s'adresse tout particulièrement aux consommateurs. Ce dépliant n'est qu'un résumé et ne donne donc pas tous les renseignements pertinents au sujet de CYKLOKAPRON. Pour toute question concernant ce médicament, communiquez avec votre médecin ou votre pharmacien.**

## AU SUJET DE CE MÉDICAMENT

**Les raisons d'utiliser ce médicament :**
CYKLOKAPRON est un médicament d'ordonnance employé pour prévenir ou réduire les hémorragies associées à différentes affections. CYKLOKAPRON peut vous avoir été prescrit pour l'une des raisons suivantes :

- Après une chirurgie mineure (p. ex., une extraction dentaire) chez les personnes présentant des troubles héréditaires de la coagulation sanguine (hémophilie)
- Chirurgie du col de l'utérus (conisation du col utérin)
- Saignements de nez (épistaxis)
- Règles abondantes (ménorragie)
- Saignement dans les yeux (hyphéma)
- Maladie héréditaire appelée œdème angioneurotique – si vous en êtes atteint, vous en avez été informé par un médecin

**Les effets de ce médicament :**
CYKLOKAPRON appartient à une catégorie de médicaments appelés agents antifibrinolytiques, qui sont employés pour arrêter les saignements excessifs après une intervention chirurgicale ou pour aider à prévenir la coagulation du sang. Lorsqu'on saigne, l'organisme forme des caillots comme mécanisme de guérison. Chez certaines personnes, les caillots ne restent pas en place assez longtemps, ce qui peut causer des saignements excessifs.

**Les circonstances où il est déconseillé d'utiliser ce médicament :**
Vous ne devez pas prendre CYKLOKAPRON si :

- vous avez des antécédents de caillots sanguins ou êtes exposés à un risque de formation de caillots sanguins (thromboembolie), particulièrement dans les jambes, les poumons et les tissus qui entourent le cerveau;
- vous êtes allergique à l'acide tranexamique, à tout autre ingrédient de ce produit ou à des médicaments semblables à celui-ci;
- vous présentez des troubles de vision des couleurs acquis;
- vous avez du sang dans votre urine ou vous présentez des saignements de provenance rénale.

**L'ingrédient médicinal :**
Acide tranexamique

**Les ingrédients non médicinaux importants :**
**Comprimés :** cellulose microcristalline, silice colloïdal anhydre, hydroxypropylcellulose, stéarate de magnésium, PEG 8000, polyvidone, talc et dioxyde de titane.

**Solution pour injection :** eau pour injection

**La présentation :**
Comprimés : 500 mg
Solution pour injection : 100 mg/mL

## MISES EN GARDE ET PRÉCAUTIONS

AVANT de prendre CYKLOKAPRON, informez-en votre médecin ou votre pharmacien si :

- vous êtes atteint d'une maladie du rein; comme le médicament peut s'accumuler dans votre organisme, il se peut qu'on vous prescrive une dose plus faible;
- vous avez des saignements provenant du haut de l'appareil urinaire qui se retrouveront dans votre urine;
- vous avez déjà eu des hémorragies non maîtrisables (y compris des menstruations irrégulières);
- vous êtes enceinte ou vous allaitez;
- vous prenez « la pilule » (contraceptif hormonal) ou un anticoagulant (médicament qui empêche le sang de trop se coaguler);
- vous êtes traité pour une coagulation intravasculaire disséminée; vous recevrez CYKLOKAPRON seulement si votre médecin vous a fait passer des tests sanguins pour vérifier si ce médicament vous convient – si ce n'est pas le cas, il se peut qu'un autre agent antiplaquettaire soit mieux indiqué pour vous;
- vous êtes obèse, diabétique, avez reçu un diagnostic de syndrome des ovaires polykystiques, avez des antécédents familiaux de cancer de l'endomètre, êtes sous œstrogénothérapie ou prenez du tamoxifène.

Vous devez informer votre médecin des médicaments d'ordonnance, des médicaments en vente libre et des produits naturels ou à base d'herbes médicinales que vous prenez.

L'emploi de CYKLOKAPRON n'est pas recommandé chez les adolescentes de moins de 18 ans qui ont un flux menstruel anormalement abondant (hyperménorrhée).

CYKLOKAPRON peut perturber la perception des couleurs. Si vous devez être traité par CYKLOKAPRON durant plusieurs semaines, un examen ophtalmique (acuité visuelle, vision des couleurs, champ de vision, etc.) est recommandé avant de commencer le traitement, et à intervalles réguliers durant le traitement. Si vous constatez un changement dans votre vision, particulièrement en ce qui a trait à la perception des couleurs, communiquez sans attendre avec votre médecin ou votre pharmacien afin qu'il vous donne des directives précises pour arrêter le traitement en toute sécurité.

Si vous prenez CYKLOKAPRON pour la prise en charge de règles abondantes, vous devez surveiller votre flux menstruel. Si les saignements menstruels n'ont pas diminué après trois cycles menstruels, consulter votre médecin pour qu'il envisage un traitement de rechange.

Si vous éprouvez des étourdissements, ne prenez pas le volant et ne manœuvrez pas de machines.

## INTERACTIONS MÉDICAMENTEUSES

Si vous prenez d'autres médicaments, informez-en votre médecin ou votre pharmacien, qu'il s'agisse de médicaments d'ordonnance ou en vente libre, ou encore de produits naturels.

**Médicaments qui sont susceptibles d'interagir avec** CYKLOKAPRON :

- tous les médicaments qui favorisent la coagulation du sang;
- médicaments qui réduisent la formation de caillots (anticoagulants); le traitement simultané avec des anticoagulants doit se faire sous l'étroite surveillance d'un médecin qualifié dans ce domaine;
- contraceptifs qui contiennent des hormones, comme la pilule anticonceptionnelle – le fait de prendre la pilule en même temps que CYKLOKAPRON peut accroître le risque de formation de caillots, d'accident vasculaire cérébral et de crise cardiaque;
- l'hydrochlorothiazide, la desmopressine, la sulbactame-ampicilline, le carbazochrome, la ranitidine et la nitroglycérine; la prise de ces médicaments avec CYKLOKAPRON peut provoquer une crise cardiaque.

## UTILISATION APPROPRIÉE DE CE MÉDICAMENT

**Posologie habituelle :**
Prenez CYKLOKAPRON exactement comme votre médecin vous l'a prescrit. Prenez toujours le nombre de comprimés que votre médecin vous a dit de prendre.
Chez l'adulte, la posologie habituelle est de 2 à 3 comprimés de 2 à 3 fois par jour. La posologie peut varier selon la raison pour laquelle le médicament a été prescrit.
Chez l'enfant, la posologie doit être calculée en fonction du poids corporel (25 mg/kg).
Il faut prescrire une dose plus faible aux patients atteints d'une maladie rénale.

La solution de CYKLOKAPRON pour injection est habituellement administrée lentement par voie intraveineuse en bolus pendant au moins 5 minutes ou par perfusion intraveineuse. Votre médecin décidera de la dose qui vous convient en fonction de votre poids.

Il faut examiner la solution avant de l'administrer afin de s'assurer de sa transparence et de l'absence de particules, de précipité, de changement de couleur ou de fuite.

**Surdose :**

En cas de surdosage, et ce, même en l'absence de symptômes, communiquez immédiatement avec un professionnel de la santé, le service des urgences d'un hôpital ou le centre antipoison de votre région.

**Dose oubliée :**
Si vous avez oublié de prendre une dose de médicament, prenez la prochaine comme d'habitude; ne prenez pas deux doses en même temps la fois suivante pour rattraper la dose oubliée. .

## EFFETS SECONDAIRES ET MESURES À PRENDRE

En plus de l'action recherchée, tout médicament peut causer des effets indésirables. Toutefois, consultez sans tarder votre médecin ou votre pharmacien si l'un des effets suivants persiste ou devient incommodant :

- Nausées (envie de vomir)
- Vomissements
- Diarrhée

Si la solution est injectée trop rapidement, il se peut que vous vous sentiez étourdi en raison d'une baisse de tension artérielle.

**EFFETS SECONDAIRES GRAVES : FRÉQUENCE ET MESURES À PRENDRE**

| Symptôme / effet | | Consultez votre médecin ou votre pharmacien | | Cessez de prendre le médicament et faites appel à un service d'urgence médicale immédiatement |
|---|---|---|---|---|
| | | Seulement pour les effets secondaires graves | Dans tous les cases | |
| Rare | Réactions allergiques (éruptions cutanées) | | | √ |
| | Occlusion de l'artère et de la veine centrales de la rétine. Changements liés à la vision (p. ex., changement de l'acuité visuelle ou du champ de vision et, surtout, changement dans la perception des couleurs) | | √ | √ |

**EFFETS SECONDAIRES GRAVES : FRÉQUENCE ET MESURES À PRENDRE**

| **Symptôme / effet** | | **Consultez votre médecin ou votre pharmacien** | | **Cessez de prendre le médicamen** |
|---|---|---|---|---|
| | Étourdissements | | √ | √ |
| | Crises ou convulsions | | √ | √ |
| | Douleur à la poitrine ou aux jambes | | √ | |
| | Crise cardiaque (douleur à la poitrine) | | √ | √ |
| | Caillots de sang/ thrombose veineuse profonde/ thrombose artérielle d'un membre (douleur, rougeur, chaleur aux mains, aux jambes, aux chevilles ou aux pieds) | | √ | √ |
| | Infarctus cérébral/ accident vasculaire cérébral/ thrombose cérébrale (difficultés à parler ou à marcher, confusion soudaine, engourdissement ou sensation de faiblesse) | | √ | √ |
| | Nécrose aiguë du cortex rénal (difficulté à uriner) | | √ | √ |
| | Baisse de la tension artérielle (peut survenir après une injection rapide). Voici les symptômes possibles : étourdissement, mal de tête et sensation d'ébriété (surtout lorsque vous changez rapidement de position) | | √ | |

***Cette liste d'effets secondaires n'est pas exhaustive. Si votre traitement par CYKLOKAPRON entraîne des effets secondaires inattendus, veuillez communiquer avec votre médecin ou votre pharmacien.***

## CONSERVATION DU MÉDICAMENT

Conserver à la température ambiante (entre 15 et 30 °C).
**À garder hors de la portée et de la vue des enfants.**

**DÉCLARATION DES EFFETS INDÉSIRABLES SOUPÇONNÉS**

**Vous pouvez déclarer les effets indésirables soupçonnés associés à l'utilisation des produits de santé au Programme Canada Vigilance de l'une des trois façons suivantes :**

**--------------------------------------------------------------------------**

- **En ligne à www.santecanada.gc.ca/medeffet**
- **Par téléphone, en composant le numéro sans frais 1-866-234-2345**
- **En remplissant un formulaire de déclaration de Canada Vigilance et en le faisant parvenir :**
  - **par télécopieur, au numéro sans frais 1-866-678-6789**
  - **par la poste :**

**Programme Canada Vigilance**
**Santé Canada**
**Indice postal 0701E**
**Ottawa (Ontario) K1A 0K9**

**Les étiquettes préaffranchies, le formulaire de déclaration de Canada Vigilance ainsi que les lignes directrices concernant la déclaration d'effets indésirables sont disponibles sur le site Web de MedEffet Canada à www.santecanada.gc.ca/medeffet.**

***REMARQUE : Pour obtenir des renseignements relatifs à la gestion des effets secondaires, veuillez communiquer avec votre professionnel de la santé. Le Programme Canada Vigilance ne fournit pas de conseils médicaux.***

## POUR DE PLUS AMPLES RENSEIGNEMENTS

Vous pouvez trouver ce document et la monographie complète du produit, préparée pour les professionnels de la santé, à l'adresse : http://www.pfizer.ca
ou en communiquant avec le promoteur, Pfizer Canada inc., au : 1-800-463-6001.
Pfizer Canada inc. a rédigé ce dépliant.
Dernière révision : le 2 décembre 2010

# Annexe 10

# Feuillet explicatif pour administration externe d'acide tranexamique

CENTRE DE RECHERCHE CHUS

Version 2
16 octobre 2015

**L'acide tranexamique comme traitement médical des hématomes sous-duraux chroniques**

# Instructions au patient

Madame,
Monsieur,

Dans le cadre de votre participation au projet de recherche « L'acide tranexamique comme traitement médical des hématomes sous-duraux chroniques », vous prenez à chaque jour un comprimé et demi d'acide tranexamique ou de placebo.

À votre sortie de l'hôpital, vous devez **continuer de prendre le médicament à raison de 1 ½ comprimé par jour, au coucher, jusqu'au dernier comprimé** ou jusqu'à ce que l'infirmière de recherche vous contacte et vous demande d'arrêter le médicament.

Conservez les comprimés avec vos médicaments usuels dans un endroit frais et sec, à l'abri de la lumière.

Pour toute question concernant le projet de recherche, contactez l'infirmière de recherche au 1 (819) 346-1110, poste 12811. Vous serez contacté par téléphone pour la planification de votre CT Scan de contrôle et votre prochain suivi.

Merci encore de votre participation.

Cordialement,

Dr David Mathieu, M.D.
Chef du service de neurochirurgie du CHUS

# Annexe 11

# Détail de l'analyse intermédiaire pré-planifiée au 65e sujet

# Analyse intérimaire pré-planifiée au 65e sujet

## Objectifs

L'analyse intermédiaire sera effectuée lorsque le suivi post-traitement du 65e sujet sera enclenché. Cette analyse a trois objectifs :

- Établir avec précision l'incidence de l'issue primaire dans notre échantillon;
- Réviser les calculs de puissance en fonction de l'incidence réelle de l'issue primaire;
- Établir l'incidence de complications thrombo-emboliques dans chacun des groupes (surveillance de la sécurité de l'intervention).

## Analyse des données

La table de randomisation des sujets au groupe A ou B sera dévoilée par la pharmacie et intégrée à la base de données en ligne pour les sujets 1 à 65. Un fichier Microsoft Excel contenant les données anonymisées sera généré et téléchargé par Dr Iorio-Morin afin d'effectuer les analyses statistiques dans le logiciel SPSS. Les données seront analysées en effectuant les tests statistiques présentés dans le tableau ci-dessous. À noter que l'assignation d'intervention pour chaque groupe (placébo vs acide tranexamique) ne sera pas dévoilée lors de l'analyse intérimaire, à moins d'une différence statistiquement et cliniquement significative (aux yeux du comité de projet) dans l'incidence d'évènements indésirables.

| Variable | Format utilisé pour l'analyse | Incertitude | Test statistique |
|---|---|---|---|
| Plan initial de traitement (% de traitement conservateur comme choix initial) | Continue | - | T de Student vs Mann-Withney * |
| Atteinte de l'issue primaire | Binaire | - | $\chi^2$ (Test exact de Fisher) |
| Résolution de l'hématome | Binaire | - | $\chi^2$ (Test exact de Fisher) |
| Récidive d'hématome sous-dural | Binaire | - | $\chi^2$ (Test exact de Fisher) |
| Drainage chirurgical | Binaire | - | $\chi^2$ (Test exact de Fisher) |
| Incidence de thrombophlébite profonde | Binaire | - | $\chi^2$ (Test exact de Fisher) |
| Incidence d'embolie pulmonaire | Binaire | - | $\chi^2$ (Test exact de Fisher) |
| Incidence d'ICT | Binaire | - | $\chi^2$ (Test exact de Fisher) |
| Incidence d'AVC | Binaire | - | $\chi^2$ (Test exact de Fisher) |
| Incidence de pseudo-néphrolithiase | Binaire | - | $\chi^2$ (Test exact de Fisher) |

* Pour évaluer l'effet de variables continues sur une issue dichotomique, le test T de Student sera employé si la distribution est normale, alors que le U de Mann-Whitney sera utilisé si la

distribution est anormale. La normalité de la distribution sera évaluée avec le test de Kolmogorov-Smirnov (si l'on dispose de ≥ 50 observations) ou de Shapiro-Wilk (si l'on dispose de < de 50 observations).

# Annexe 12

# Formulaire de protocole opératoire

# ÉTUDE TRACS – 14-213

## L'acide tranexamique comme traitement médical des hématomes sous-duraux chroniques

# Protocole opératoire

No sujet ________________________________

## Procédure réalisée à DROITE

| | |
|---|---|
| Contexte de la chirurgie | ☐ Aucune chirurgie effectuée à droite<br>☐ Chirurgie élective<br>☐ Chirurgie d'urgence (qui n'aurait pas pu attendre au lendemain) |
| Date de la chirurgie | ________________________________ |
| Type de chirurgie | ☐ Trépanation simple à droite<br>☐ Trépanation double à droite<br>☐ Craniotomie droite<br>☐ Autre : ______________________ |
| Lieu de la chirurgie | ☐ Bloc opératoire<br>☐ Draînage au chevet |
| Installation de drain | ☐ Non<br>☐ Oui, drain sous-dural droit<br>☐ Oui, drain sous-galéal droit<br>☐ Oui, autre : __________________ |
| Orientation du drain | ☐ Aucun drain<br>☐ Extrémité en antérieur<br>☐ Extrémité en postérieur |
| Irrigation intra-opératoire | ☐ Non<br>☐ Oui |
| Mobilisation post-opératoire | ☐ Repos au lit<br>☐ Mobilisation selon tolérance |

## Procédure réalisée à GAUCHE

| | |
|---|---|
| Contexte de la chirurgie | ☐ Aucune chirurgie effectuée à gauche<br>☐ Chirurgie élective<br>☐ Chirurgie d'urgence (qui n'aurait pas pu attendre au lendemain) |
| Date de la chirurgie | ________________________________ |
| Type de chirurgie | ☐ Trépanation simple à gauche<br>☐ Trépanation double à gauche<br>☐ Craniotomie gauche<br>☐ Autre : ______________________ |
| Lieu de la chirurgie | ☐ Bloc opératoire<br>☐ Draînage au chevet |
| Installation de drain | ☐ Non<br>☐ Oui, drain sous-dural gauche<br>☐ Oui, drain sous-galéal gauche<br>☐ Oui, autre : __________________ |
| Orientation du drain | ☐ Aucun drain<br>☐ Extrémité en antérieur<br>☐ Extrémité en postérieur |
| Irrigation intra-opératoire | ☐ Non<br>☐ Oui |
| Mobilisation post-opératoire | ☐ Repos au lit<br>☐ Mobilisation selon tolérance |

Signature ______________________________ Date ______________________________

CENTRE DE RECHERCHE | CHUS

Version 5
27 avril 2016

**ÉTUDE TRACS – 14-213**

**L'acide tranexamique comme traitement médical des hématomes sous-duraux chroniques**

# Protocole de prélèvements au bloc opératoire

**Vous aurez besoin de :**
1 Specimen trap
1 tube lavande
1 tube bleu

En pré-op :

- **Prescrire** tous les prélèvements chirurgicaux et **imprimer** les étiquettes :
  - Dans Ariane, dans la section ordonnance, cliquer sur le catalogue « Neurochirurgie » → « Projets de recherche » → « TRACS »
  - Prescrire tous les prélèvements de la section « Bilan chirurgical au bloc OP » et cocher « Aujourd'hui »
  - Imprimer toutes les **étiquettes**
  - Ré-imprimer une **2e étiquette** pour le « décompte cellulaire sur liquide »

- Demander à l'anesthésie d'effectuer les prélèvements à l'induction :
  - FSC
  - Glycémie
  - Protéine sérique
  - Osmolarité sérique
  - CIVD

- Prescrire la tomodensitométrie axiale de contrôle post-opératoire
- Rappeler au chirurgien de remplir son questionnaire de protocole opératoire

Per-op :

- Prélever l'hématome à l'aide d'un « spécimen trap »
- Transvider l'hématome le plus rapidement possible après le prélèvement :
  - Remplir un **tube bleu** pour le décompte cellulaire et écrire à la main « CIVD » sur l'étiquette de décompte cellulaire
  - Remplir un **tube lavande** pour la 2e étiquette de décompte cellulaire et écrire à la main « décompte + plaquettes »
  - Utiliser le « **spécimen trap** » comme contenant pour l'osmolarité, le glucose et les protéines sur liquide

- Prélever un échantillon de membrane à l'aide de la petite pince à biopsie
  - Déposer dans un **pot de formol** et envoyer en pathologie
  - En prescrivant la pathologie, ajouter dans en commentaire « Projet TRACS 14-213 »
