# Supplementary material for: Tranexamic Acid in Chronic Subdural Hematomas (TRACS): study protocol for a randomized controlled trial
Source: Trials. 2016 May 5;17:235. doi: 10.1186/s13063-016-1358-5 (PMC4857422; doi:10.1186/s13063-016-1358-5)
Supplement: Additional file 3: — Consent form (original French version). (PDF 253 kb) [file 13063_2016_1358_MOESM3_ESM.pdf]

CENTRE DE RECHERCHE | CHUS

FORMULAIRE D'INFORMATION ET DE CONSENTEMENT A LA RECHERCHE

**Titre du projet :**	L'acide tranexamique comme traitement médical des hématomes sous-duraux chroniques
**Numéro de projet :**	14-213
**Chercheur principal :**	Dr David Mathieu, M.D.<br>Service de neurochirurgie<br>Département de chirurgie
**Chercheurs associés:**	Dr Christian Iorio-Morin, M.D., Ph.D., R2<br>Service de neurochirurgie<br>Département de Chirurgie<br><br>Dr Jocelyn Blanchard, M.D.<br>Service de neurochirurgie<br>Département de chirurgie

EN CAS D'URGENCE

Du lundi au vendredi entre 7h et 17h, vous pouvez communiquer avec :

Dr Christian Iorio-Morin, R2	Tél : (819) 346-1110, faites le « 0 » et demandez à la téléphoniste de signaler la pagette 9721
Mme Josée Soucy inf.,	Téléphone : (819) 346-1110 poste 16364 ou faites le «0» et demandez à la téléphoniste de signaler la pagette 8359.
Mme Josée Ducharme inf.,	Téléphone : (819) 346-1110 poste 16369 ou faites le «0» et demandez à la téléphoniste de signaler la pagette 8423.

En dehors de ces heures de bureau, veuillez vous présenter à la salle d'urgence la plus proche et mentionner que vous participez à un projet de recherche.

PRÉAMBULE POUR LE CONSENTEMENT SUBSTITUÉ

Ce projet de recherche s'adresse à des personnes majeures qui, par leur état de santé, pourraient ne pas être en mesure d'exprimer leur volonté d'y participer ou non. Dans un tel cas, la loi prévoit que le consentement peut être donné par le conjoint, à défaut ou en cas d'empêchement de celui-ci, par un proche parent ou par une personne qui démontre pour le sujet un intérêt particulier. Si vous êtes cette personne, nous vous demanderons, le cas échéant, de signer le formulaire de consentement qui indiquera votre accord à la participation du sujet à ce projet de recherche. La décision que vous prendrez doit refléter l'intérêt du sujet et tenir compte, dans la mesure du possible, des volontés qu'il a pu exprimer dans le passé.

Afin d'alléger le texte qui suit, le pronom « vous » indique la personne que vous représentez.

APPROUVÉ LE 9 FÉVRIER 2016
CER CHEZ L'HUMAIN DU CHUS

INTRODUCTION

Nous sollicitons votre participation à un projet de recherche parce que vous avez reçu un diagnostic d'hématome sous-dural chronique. Cependant, avant d'accepter de participer à ce projet, veuillez prendre le temps de lire, de comprendre et de considérer attentivement les renseignements qui suivent. Si vous acceptez de participer au projet de recherche, vous devrez signer le formulaire de consentement à la fin du présent document et nous vous en remettrons une copie pour vos dossiers.

Ce formulaire d'information et de consentement vous explique le but de ce projet de recherche, les procédures, les risques et inconvénients ainsi que les avantages, de même que les personnes avec qui communiquer au besoin. Il peut contenir des mots que vous ne comprenez pas. Nous vous invitons à poser toutes les questions nécessaires au chercheur responsable du projet ou aux autres personnes affectées au projet de recherche et à leur demander de vous expliquer tout mot ou renseignement qui n'est pas clair.

NATURE ET OBJECTIFS DU PROJET DE RECHERCHE

Aucun médicament n'est actuellement démontré efficace pour guérir les hématomes sous-duraux chroniques. L'objectif de l'étude est de déterminer si l'acide tranexamique pourrait accélérer la résolution des hématomes comparé à un placebo.

DÉROULEMENT DU PROJET DE RECHERCHE

Le traitement de votre hématome sous-dural chronique peut inclure l'observation (laisser l'hématome disparaître seul) ou la chirurgie et sera discuté avec vous par votre médecin indépendemment de cette étude. Si vous acceptez de participer à l'étude, en plus du traitement choisi par votre médecin et vous, vous aurez à prendre un médicament (acide tranexamique ou placebo) une fois par jour jusqu'à résolution de l'hématome, pour une durée maximale de 20 semaines s'il n'a a pas de résolution.

L'acide tranexamique est un médicament utilisé fréquemment pour diminuer les saignements en traumatologie, en gynécologie, en chirurgie cardiaque et en chirurgie orthopédique. Quelques rapports suggèrent que l'administration d'acide tranexamique pendant quelques semaines pourrait guérir les hématomes sous-duraux sans avoir recours à la chirurgie

Tout au long du suivi, vous devrez passer 2 à 5 tomodensitométries axiales (CT Scan) de contrôle. Ces tomodensitométries axiales font partie du suivi médical usuel et sont faites peu importe si vous participez ou non à l'étude. Toutefois, si vous acceptez de participer à l'étude, l'une des tomodensitométrie sera supplémentée d'une injection d'iode afin d'évaluer la quantité de vaisseaux sanguins contribuant à l'hématome. De plus, vous aurez à rencontrer le personnel de recherche au Centre de recherche du CHUS à 3 reprises. Afin de minimiser vos déplacements, ces rencontres seront planifiées le même jour que les tomodensitométries axiales.

Les 3 rencontres sont :

- La visite de sélection et d'initiation du traitement
- La visite de suivi
- La visite de fin d'étude

Si vous devez avoir une chirurgie pour draîner votre hématome sous-dural, un échantillon de l'hématome sera prélevé et analysé au laboratoire. Notez que cette procédure n'altère aucunement le déroulement de la chirurgie.

Visite de sélection et d'initiation du traitement :
Durant cette visite, d'une durée approximative de 60 minutes, le personnel de recherche validera si vous êtes admissible à l'étude. Des données médicales et sociodémographiques de base seront recueillies et vous passerez une tomographie axiale (CT Scan) injectée à l'iode. Si vous êtes allergique à l'iode ou êtes insuffisant rénal, cet examen ne sera pas réalisé. Vous aurez également à répondre à un questionnaire évaluant différents aspects incluant l'autonomie, la qualité de vie et les fonctions cognitives.

Vous serez assigné au hasard à l'un de deux groupes :

- Groupe 1 : Placebo
- Groupe 2 : Acide tranexamique

Vous avez une chance sur deux d'être assigné à l'un ou l'autre des groupes. Le placebo est une substance inactive qui a la même apparence que le médicament actif. Puisque cette étude est à double insu, ni vous, ni l'équipe de recherche ne saurez à quel groupe vous appartenez. Cependant, en cas d'urgence, le médecin de l'étude pourra obtenir cette information rapidement.

Les médicaments vous seront finalement distribués et un rendez-vous de suivi planifié dans 10 semaines.

Visite de suivi :
La visite de suivi aura lieu après 10 semaines et durera approximativement 30 minutes. Vous aurez à répondre une fois de plus à différents questionnaires. Si l'hématome sous-dural n'est pas encore résolu à la tomodensitométrie axiale, le traitement sera prolongé de 10 semaines supplémentaires et les comprimés nécessaires vous seront remis.

Visite de fin de traitement :
Lorsque vous aurez définitivement cessé de recevoir le médicament à l'étude, vous devrez vous présenter au centre de recherche pour une visite de fin de traitement. Cette visite durera environ 30 minutes pendant lesquelles vous aurez à remplir différents questionnaires en présence du personnel de recherche.

Voici la description des différents tests et procédures qui seront réalisés pendant votre participation à l'étude. **SVP, vous référer au calendrier à la fin de ce document pour une vision d'ensemble des examens et procédures durant l'étude.**

- Histoire médicale
- Questionnaires (20 minutes total)
- Prises de sang
- Tomodensitométrie axiale avec injection
- Tomodensitométries axiales de suivi (3 à 5 selon l'évolution de l'hématome)
- Analyse de l'hématome (si vous avez une chirurgie)

Votre dossier médical sera consulté tout au long de l’étude par le chercheur et son équipe de recherche. Par ailleurs, les résultats des tests, procédures et examens médicaux effectués dans le cadre de ce projet de recherche peuvent apparaître dans votre dossier médical.

RISQUES POUVANT DÉCOULER DE VOTRE PARTICIPATION AU PROJET DE RECHERCHE

Les risques reliés à l’administration d’acide tranexamique sont les suivants :

- Effets secondaires fréquents (>10%) :
  - Maux de tête
  - Nausées
  - Vomissements
  - Diarrhée
  - Douleur musculaire ou lombaire

- Effets secondaires rares (1-10%) :
  - Fatigue
  - Anémie
  - Douleur aux articulations
  - Crampes musculaires

- Effets secondaires très rares (< 1%) :
  - Réactions allergiques (éruptions cutannées)
  - Occlusion de l’artère et de la veine centrales de la rétine de l’oeil
  - Changements liés à la vision (p. ex., changement de l’acuité visuelle ou du champ de vision et, surtout, changement dans la perception des couleurs)
  - Étourdissements
  - Crises ou convulsions
  - Douleur à la poitrine ou aux jambes
  - Crise cardiaque (douleur à la poitrine)
  - Caillots de sang/thrombose veineuse profonde/thrombose artérielle d’un membre (douleur, rougeur, chaleur aux mains, aux jambes, aux chevilles ou aux pieds)
  - Infarctus cérébral/accident vasculaire cérébral/thrombose cérébrale (difficultés à parler ou à marcher, confusion soudaine, engourdissement ou sensation de faiblesse)
  - Nécrose aiguë du cortex rénal (difficulté à uriner)
  - Baisse de la tension artérielle (étourdissement, mal de tête et sensation d’ébriété)

Si vous ressentez l’un des effets secondaires décrits plus haut, veuillez contacter Dr Christian Iorio-Morin au numéro sur la page couverture ou vous présenter à la salle d’urgence en mentionnant que vous participez à un projet de recherche.

Les risques reliés à l’injection d’iode sont les suivants : allergie au produit de contraste et insuffisance rénale.

RISQUES ASSOCIÉS À LA GROSSESSE

Votre participation à cette recherche peut comporter des risques, connus ou non, pour les femmes enceintes, les embryons, les fœtus ou encore les nourrissons allaités au sein. C'est pourquoi les femmes enceintes ou qui allaitent ne peuvent participer à ce projet. Les femmes susceptibles de devenir enceintes doivent subir un test de grossesse avant le début de leur participation au projet. De plus, elles doivent absolument utiliser une méthode contraceptive acceptable du point de vue médical tout au long de leur participation au projet de recherche. Le médecin de l'étude ou le personnel du projet de recherche vérifiera votre méthode contraceptive afin de s'assurer qu'elle est acceptable du point de vue médical. Si vous pensez être devenue enceinte durant l'étude, vous devrez le signaler immédiatement au chercheur responsable du projet de recherche afin de discuter avec lui des différentes options.

INCONVÉNIENTS POUVANT DÉCOULER DE VOTRE PARTICIPATION AU PROJET DE RECHERCHE

Le principal inconvénient découlant de votre participation au projet de recherche est le temps consacré à répondre aux questionnaires.

AVANTAGES POUVANT DÉCOULER DE VOTRE PARTICIPATION AU PROJET DE RECHERCHE

Il se peut que vous retiriez un bénéfice personnel de votre participation à ce projet de recherche, mais nous ne pouvons pas le garantir. Par ailleurs, les informations découlant de ce projet de recherche pourraient contribuer à l'avancement des connaissances dans le domaine des hématomes sous-duraux chroniques.

ALTERNATIVE À LA PARTICIPATION AU PROJET DE RECHERCHE

Vous n'êtes pas obligé de participer à ce projet de recherche pour être traité pour votre maladie. La participation ne fait qu'ajouter un traitement médical dont l'efficacité n'est pas démontrée.

PARTICIPATION VOLONTAIRE ET POSSIBILITÉ DE RETRAIT DU PROJET DE RECHERCHE

Votre participation à ce projet de recherche est volontaire. Vous êtes donc libre de refuser d'y participer. Vous pouvez également vous retirer de ce projet à n'importe quel moment, sans avoir à donner de raisons, en faisant connaître votre décision au chercheur responsable du projet ou à l'un de ses assistants.

Votre décision de ne pas participer à ce projet de recherche ou de vous en retirer n'aura aucune conséquence sur la qualité des soins et des services auxquels vous avez droit ou sur vos relations avec le chercheur responsable du projet et les autres intervenants.

Toute nouvelle connaissance acquise durant le déroulement de l'étude qui pourrait affecter votre décision de continuer d'y participer vous sera communiquée sans délai.

Si vous vous retirez de l'étude ou en êtes retiré, l'information médicale déjà obtenue dans le cadre de l'étude sera conservée aussi longtemps que nécessaire pour assurer la sécurité des patients et rencontrer les exigences réglementaires.

ARRÊT DU PROJET DE RECHERCHE

Le chercheur responsable de l'étude, l'organisme subventionnaire et le Comité d'éthique de la recherche en santé chez l'humain du CHUS peuvent mettre fin à votre participation, sans votre consentement, pour les raisons suivantes :

- Si de nouveaux développements scientifiques survenaient indiquant qu'il est de votre intérêt de cesser votre participation;
- Si le chercheur responsable du projet pense que cela est dans votre meilleur intérêt;
- Si vous ne respectez pas les consignes du projet de recherche;
- S'il existe des raisons administratives d'abandonner l'étude.

CONFIDENTIALITÉ

Durant votre participation à ce projet, le chercheur responsable ainsi que son personnel recueilleront et consigneront dans un dossier de recherche les renseignements vous concernant. Seuls les renseignements nécessaires pour répondre aux objectifs scientifiques de ce projet seront recueillis.

Ces renseignements peuvent comprendre les informations contenues dans vos dossiers médicaux concernant votre état de santé passé et présent, vos habitudes de vie ainsi que les résultats de tous les tests, examens et procédures que vous aurez à subir durant ce projet. Votre dossier peut aussi comprendre d'autres renseignements tels que votre nom, date de naissance, sexe et origine ethnique.

Tous les renseignements recueillis demeureront strictement confidentiels dans les limites prévues par la loi. Afin de préserver votre identité et la confidentialité des renseignements, vous ne serez identifié que par un numéro de code. La clé du code reliant votre nom à votre dossier de recherche sera conservée par le chercheur responsable du projet de manière sécuritaire.

Les données en elles-mêmes ou combinées aux données provenant d'autres projets, pourront être partagées avec les organismes réglementaires canadiens ou d'autres pays ou avec les partenaires commerciaux du commanditaire. Les données de recherche seront conservées pendant 25 ans par le chercheur responsable.

De plus, les données du projet pourraient servir à obtenir l'approbation de mise en marché du médicament à l'essai par les organismes réglementaires autorisés. Elles pourraient aussi servir pour d'autres analyses de données reliées au projet ou pour l'élaboration de projets de recherches futurs.

Les données pourront être publiées dans des revues spécialisées ou faire l'objet de discussions scientifiques mais il ne sera pas possible d'identifier les participants.

À des fins de surveillance et de contrôle, votre dossier de recherche ainsi que vos dossiers médicaux pourront être consultés par une personne mandatée par le Comité d'éthique de la recherche en santé chez l'humain du CHUS ou par l'établissement, par une personne mandatée ou par des organismes publiques autorisés. Toutes ces personnes et ces organismes adhèrent à une politique de confidentialité.

À des fins de protection, notamment afin de pouvoir communiquer avec vous rapidement, vos noms et prénoms, vos coordonnées et la date de début et de fin de votre participation au projet seront conservés pendant un an après la fin du projet dans un répertoire sécurisé maintenu par le chercheur.

Vous avez le droit de consulter votre dossier de recherche pour vérifier les renseignements recueillis, et les faire rectifier au besoin et ce, aussi longtemps que le chercheur responsable du projet ou le CHUS détiennent ces informations. Cependant, afin de préserver l'intégrité scientifique du projet, vous pourriez n’avoir accès à certaines de ces informations qu'une fois le projet terminé.

COMPENSATION
Vous ne recevrez aucune compensation pour votre participation à ce projet de recherche.

DROITS DU PARTICIPANT ET INDEMNISATION EN CAS DE PRÉJUDICE
Si vous deviez subir quelque préjudice que ce soit dû à votre participation au projet de recherche, vous recevrez tous les soins et services requis par votre état de santé, sans frais de votre part.

En acceptant de participer à cette étude, vous ne renoncez à aucun de vos droits ni ne libérez les chercheurs, le commanditaire ou l’établissement où se déroule ce projet de recherche de leurs responsabilités civile et professionnelle.

FINANCEMENT DU PROJET DE RECHERCHE
Le chercheur a reçu des fonds de l’organisme subventionnaire pour mener à bien ce projet de recherche.

PERSONNES-RESSOURCES
Si vous avez des questions concernant votre participation au projet de recherche SVP vous référer à l’encadré de la page 1.

Pour toute question concernant vos droits en tant que participant à ce projet de recherche ou si vous avez des plaintes ou des commentaires à formuler vous pouvez communiquer avec le Commissaire aux plaintes et à la qualité des services du CIUSSS de l’Estrie-CHUS au numéro suivant : 1-866-917-7903.

SURVEILLANCE DES ASPECTS ÉTHIQUES
Le Comité d’éthique de la recherche en santé chez l’humain du CHUS a approuvé ce projet de recherche et en assure le suivi. De plus, nous nous engageons à lui soumettre pour approbation toute révision et toute modification apportée au protocole de recherche ou au formulaire d’information et de consentement.

Si vous désirez rejoindre l’un des membres de ce comité vous pouvez communiquer avec le Service de soutien à l’éthique de la recherche du CHUS au numéro 819-346-1110, poste 12856.

CONSENTEMENT

Je déclare avoir lu le présent formulaire d'information et de consentement, particulièrement quant à la nature de ma participation au projet de recherche et l'étendue des risques qui en découlent. Je reconnais qu'on m'a expliqué le projet, qu'on a répondu à toutes mes questions et qu'on m'a laissé le temps voulu pour prendre une décision.

Je consens librement et volontairement à participer à ce projet.

Nom du participant *(lettres moulées)*	Signature du participant	Date

Nom de la personne qui obtient le consentement *(lettres moulées)*	Signature de la personne qui obtient le consentement	Date

ENGAGEMENT DU CHERCHEUR

Je certifie qu'on a expliqué au participant les termes du présent formulaire d'information et de consentement, que j'ai répondu aux questions que le participant avait à cet égard et que j'ai clairement indiqué qu'il demeure libre de mettre un terme à sa participation, et ce, sans préjudice.

Je m'engage à respecter ce qui a été convenu au formulaire d'information et de consentement et à en remettre copie signée au participant. J'ai participé au processus de sollicitation du participant et je confirme l'avoir avisé explicitement de mon double rôle (médecin traitant et chercheur)

Nom du chercheur (lettres moulées)	Signature du chercheur	Date

CONSENTEMENT DU REPRÉSENTANT (MAJEUR SUBITEMENT INAPTE)

Du fait que M/Mme ________________________ a été rendu(e) subitement inapte à consentir pour la raison identifiée ci-après, le Code civil du Québec vous autorise, en tant que ____________________________ (votre lien avec le participant) à consentir pour lui(elle) à participer à ce projet de recherche.
Dès que M./Mme _____________________ sera suffisamment rétabli(e), nous l’inviterons à signer lui(elle)-même le formulaire de consentement afin qu’il(elle) puisse signifier son désir de poursuivre, ou non, sa participation à l’étude.

RAISON POUR LAQUELLE LE PARTICIPANT NE PEUT CONSENTIR :

____

En signant cette page, je déclare avoir lu le présent formulaire d’information et de consentement. Je reconnais qu’on m’a expliqué le projet, qu’on a répondu à toutes mes questions et qu’on m’a laissé le temps voulu pour prendre une décision. Je donne volontairement mon accord pour que _________________ participe à cette étude.

____

Nom du représentant<br>(Lettres moulées)	Signature du représentant	Date

J’ai expliqué au représentant tous les aspects pertinents de la recherche et j’ai répondu aux questions qu’il (elle) m’a posées. Je lui ai indiqué que la participation au projet de recherche est libre et volontaire et que la participation peut être cessée en tout temps.
Je m’engage à respecter ce qui a été convenu au formulaire d’information et de consentement et à en remettre copie signée au représentant du participant.

____

Nom de la personne qui obtient le consentement<br>(Lettres moulées)	Signature de la personne qui obtient le consentement	Date

CONSENTEMENT DU REPRÉSENTANT (Majeur inapte)

Du fait que M/Mme ______________________________ est inapte à consentir pour la raison identifiée ci-après, en tant que son représentant légal, vous devez signer cette page pour que le patient participe à ce projet de recherche.

RAISON POUR LAQUELLE LE PARTICIPANT NE PEUT CONSENTIR :

____

Assentiment verbal du participant capable de comprendre la nature de ce projet:
Oui:____ Non:____NA : ____ (pour le participant incapable de comprendre la nature de ce projet)

____

Nom du participant (Lettres moulées)	Signature du participant (si capable de signer)	Date

____

Nom du représentant légal (Lettres moulées)	Signature et titre	Date

J'ai expliqué au représentant tous les aspects pertinents de la recherche et j'ai répondu aux questions qu'il (elle) m'a posées. Je lui ai indiqué que la participation au projet de recherche est libre et volontaire et que la participation peut être cessée en tout temps. Je m'engage à respecter ce qui a été convenu au formulaire d'information et de consentement et à en remettre copie signée au représentant du sujet de recherche.

____

Nom de la personne qui obtient le consentement (Lettres moulées)	*Signature de la personne qui obtient le consentement*	*Date*

CALENDRIER DES VISITES ET INTERVENTIONS

# Visites	Visite de sélection			Visite de suivi			Visite de fin de traitement
**Semaine de traitement**	**-2 à 0**	**1**	**5**	**10**	**15**	**20**	**Fin + 12**
**Durée des visites (en minutes)**	60			30			30
Consentement	**X**						
Questionnaire médical	**X**						
Questionnaires	**X**			**X**			**X**
Prises de sang	**X**						
Tomodensitométrie axiale (CT Scan)	**X**		**X**	**Au besoin**	**Au besoin**	**Au besoin**	**X**
Tomodensitométrie axiale injectée		**X**					
Analyse de l'hématome		**Au besoin**					
Suivi des médicaments		**En continu**					
Suivi des effets indésirables		**En continu**					
