# Supplementary material for: Tranexamic Acid in Chronic Subdural Hematomas (TRACS): study protocol for a randomized controlled trial
Source: Trials. 2016 May 5;17:235. doi: 10.1186/s13063-016-1358-5 (PMC4857422; doi:10.1186/s13063-016-1358-5)
Supplement: Additional file 4: — Consent form (English translation). (PDF 324 kb) [file 13063_2016_1358_MOESM4_ESM.pdf]

CENTRE DE RECHERCHE | CHUS

RESEARCH INFORMATION AND CONSENT FORM

**Project title:**	Tranexamic acid as a medical treatment for chronic subdural hematomas
**Study number:**	14-213
**Principal instigator:**	Dr. David Mathieu, M.D.<br>Neurosurgery Division<br>Department of Surgery
**Co-investigators:**	Dr. Christian Iorio-Morin, M.D., Ph.D., R2<br>Neurosurgery Division<br>Department of Surgery
	Dr. Jocelyn Blanchard, M.D.<br>Neurosurgery Division<br>Department of Surgery

<u>IN CASE OF EMERGENCY</u>

From Monday through Friday, from 8 a.m. to 5 p.m., you can reach:

Dr. Christian Iorio-Morin, R2	Tel.: (819) 346-1110, dial « 0 » and ask the operator to call his pager #9721
Ms. Josée Soucy, R.N.,	Telephone: 819-346-1110 ext.16364 or dial "0" and ask the operator to call her pager # 8359.
Ms. Josée Ducharme, R.N.,	Telephone: 819-346-1110 ext.16369 or dial "0" and ask the operator to call her pager # 8423.

Outside office hours, please go to the closest emergency room and inform the ER that you are participating in a research project.

PREAMBLE FOR SUBSTITUTE CONSENT

This project is designed for adults who, because of their health condition, may not be able to decide whether or not they should participate. In such a case, the law authorizes the spouse to give consent for the patient; if he/she is unavailable or incapable of giving it, a close relative or a person who shows a special interest for the patient can do so. If you are that person, we will ask you, should the case arise, to sign the consent form that will indicate your agreement to the subject's participation in this research project. The decision you make must be consistent with the subject's interests and take into account, as much as possible, any wish he may have expressed in the past.

For an easier reading, the pronoun "you" in the rest of the text indicates the person you represent.

APPROUVÉ LE 9 FÉVRIER 2016
CER CHEZ L'HUMAIN DU CHUS

INTRODUCTION

We are asking for your participation in a research study, because you have been diagnosed with a chronic subdural hematoma. However, before you accept to participate in this study, take the time to read, understand and carefully think about the following information. If you accept to take part in this research study, you will have to sign the consent form at the end of this document and we will give you a signed copy for your own records.

In this Information and Consent Form you will find explanations about the goals of the study, its procedures, risks, inconveniences and advantages as well as the names of the persons you may contact if needed. This document may contain words that you do not understand. Please feel free to ask the investigator in charge of the study or other members of the study team to answer your questions and explain any word or information you do not understand.

NATURE AND PURPOSE OF THE RESEARCH STUDY

To date, no medication has been shown effective in curing chronic subdural hematomas. The purpose of this study is to determine whether tranexamic acid, compared with a placebo, could resolve hematomas faster.

CONDUCT OF THE STUDY

Your chronic subdural hematoma could be treated by observation (hematoma left to disappear by itself) or by surgery; your physician will discuss this with you, independently from the study. If you agree to participate in the study, in addition to the treatment you and your doctor choose, you will have to take either tranexamic acid or a placebo once a day until the hematoma is resolved or for a maximum of 20 weeks if it persists.

Tranexamic acid is a medication frequently used to reduce bleeding in heart surgery, in gynaecologic and orthopaedic surgeries and in trauma patients. Some reports suggest that giving tranexamic acid for a few weeks could cure subdural hematomas without using surgery.

Whether you participate or not in the study, as part of the regular follow-up, you will have to undergo 2 to 5 computerized axial tomographies (CAT-scans). However, if you accept to participate in the study, one of those CAT-scans will be done with an iodine injection to evaluate the amount of blood vessels implicated in the hematoma. In addition, you will have to visit the Research Centre of the CHUS 3 times to meet with the research personnel. Those visits will be scheduled on the same days as the CAT-scans, to reduce your amount of travel.

The three meetings will be:

- Screening visit and beginning of treatment
- Follow-up visit
- End of study visit

If you must undergo surgery to drain your subdural hematoma, a sample of the hematoma will be taken and analyzed. That procedure does not modify the surgery proceedings in any way.

Screening visit and beginning of treatment:

During that visit (about 60 minutes), the research staff will validate your eligibility to participate in the study. Medical and sociodemographic data will be collected and you will be given an iodine injection to undergo a CAT-scan. If you are allergic to iodine or if you have kidney failure, that exam will not be done. You will also have to fill in a questionnaire to evaluate several aspects of your life, including your self-sufficiency, your quality of life and your cognitive functions.

You will randomly be assigned to one of the following groups:

- Group 1: Placebo
- Group 2: Tranexamic acid

You have one in two chances to be assigned to either group. Placebo is an inactive substance that looks like the active medication. Since this study is double-blinded, neither you nor the study team will know to which treatment group you will belong. However, in case of an emergency, the study doctor will be able to get that information quickly.

Finally, we will give you the medication and set a follow-up appointment 10 weeks later.

Follow-up Visit:

The follow-up visit (10 week later) will last approximately 30 minutes. You will have to fill in a few questionnaires again. If the CAT-scan shows that the subdural hematoma is still there, the treatment will be extended for 10 more weeks and we will give you the medication you need.

End of Treatment Visit:

When you have definitely stopped taking the study medication, you will visit the Research Centre for your end-of-treatment visit. That visit will last about 30 minutes during which you will be asked to fill in some questionnaires with the research staff.

The following tests and procedures will be done during your participation in the study: Please refer to the Study Schedule at the end of this document for a comprehensive view of the examinations and procedures of the study.

- Medical history
- Questionnaires (20 minutes total)
- Blood draws
- CAT-scan with injection
- Follow-up CAT-scans (3 to 5, depending on the evolution of the hematoma)
- Analysis of the hematoma (if you undergo surgery)

Your medical records will be examined all through this research study by the investigator and his research team. Besides, results of tests, procedures and medical exams done for research purposes may appear in your medical record.

RISKS THAT MAY RESULT FROM YOUR PARTICIPATION IN THIS RESEARCH STUDY

The risks associated with the tranexamic acid intake are the following:

- Common side-effects (>10%):
  - Headache
  - Nausea
  - Vomiting
  - Diarrhoea
  - Muscular or lower-back pain

- Rare side-effects (1-10%):
  - Fatigue
  - Anaemia
  - Joint pain
  - Muscular cramps

- Very rare side-effects (< 1%):
  - Allergic reactions (rash)
  - Occlusion of the central retinal artery and vein
  - Vision changes (e.g. in visual acuity or visual field and, mainly, changes in perception of colours)
  - Dizziness
  - Seizures or convulsions
  - Chest or leg pain
  - Heart attack (chest pain)
  - Blood clots/ deep venous thrombosis/ arterial thrombosis in a limb (pain, redness, heat in hands, legs, ankles or feet)
  - Cerebral infarction/ stroke/ cerebral thrombosis (difficulty talking or walking, sudden confusion, numbness or sensation of weakness)
  - Acute necrosis of the renal cortex (difficulty to urinate)
  - Decreased blood pressure (dizziness, headache and sensation of being inebriated)

If you experience any of those side effects, please reach Dr. Christian Iorio-Morin at the number written on the first page or go to the Emergency Room and say that you are participating in a research study.

Risks associated with iodine injections are: allergy to the contrast product, and renal failure.

RISK ASSOCIATED WITH PREGNANCY

Taking part in this research study may include known or unknown risks to pregnant women, embryos, foetuses, or breastfed infants. Therefore pregnant or breastfeeding women cannot participate in this study. Women liable to become pregnant must take a pregnancy test before participating in the study. Furthermore, they absolutely must use a medically acceptable birth-control method all through their participation in the research study. The study doctor or the study staff will check your birth control method to make sure it is medically acceptable. If you think you have become pregnant during the study, you must inform the study investigator at once to discuss various options.

INCONVENIENCES POSSIBLY RESULTING FROM YOUR PARTICIPATION IN THE RESEARCH STUDY.

The main inconvenience resulting from your participation in the study is the time needed to fill in the questionnaires.

BENEFITS POSSIBLY RESULTING FROM YOUR PARTICIPATION IN THE RESEARCH STUDY

You may personally benefit from your participation in this research study, but it cannot be guaranteed. However, the information resulting from this study may help increase our knowledge about chronic subdural hematomas.

ALTERNATIVES TO YOUR PARTICIPATION IN THIS RESEARCH STUDY

You do not have to participate in this research study to be treated for your condition. Participation merely adds a medical treatment, the efficiency of which is not demonstrated.

VOLONTARY PARTICIPATION TO AND POSSIBLE WITHDRAWAL FROM THE RESEARCH STUDY

Your participation in this research study is voluntary. Thus, you are free to refuse to participate. You can also withdraw from the study at any time, without providing any reason, by notifying the investigator in charge of the study or one of his assistants.

Your decision not to participate or to withdraw from the study will not have any consequences on the quality of care and services you are entitled to or on your relationship with the investigator in charge or other caregivers.

We will inform you without delay of any new knowledge acquired during the study procedures and that could influence your decision to keep on participating.

If you withdraw or are withdrawn from the study, your medical information already collected during the study will be kept as long as necessary to insure patients' safety and to meet regulatory requirements.

TERMINATION OF STUDY

The study investigator, the funding agency or the Research Ethics Board of the CHUS could end your participation without your consent at any time for the following reasons:

- If new scientific information was made available and showed that it is in your best interest to stop participating;
- If the investigator in charge of the study thinks it is in your best interest;
- If you do not follow the study instructions;
- If there are administrative reasons to abandon the study.

CONFIDENTIALITY

While you participate in this research study, the study doctor and his staff will collect and record information about you in a research file. Only the information needed to meet the scientific objectives of the study will be collected.

This information could include data taken from your medical record concerning your past and present medical history, your lifestyle and results from all the tests, exams and procedures you will undergo during the study. Your file could also contain other information, such as your name, gender, date of birth and ethnic origin.

The information collected will be kept strictly confidential to the extent permitted by law. To protect your identity and confidentiality of your information, you will only be identified by a code number. The code key linking your identity and your research file will be kept securely by the study investigator.

Your data, combined with data collected from other studies, could be shared with regulatory agencies from Canada or other countries, or with the study sponsor's business partners. The investigator in charge will keep the research data for a 25-year period.

In addition, the study data may be used to obtain marketing approval of the study drug from authorized regulatory agencies. The study data could also be used for other analyses related to the study or to develop future research studies.

The study data may be published in specialized journals or discussed during scientific meetings, but it will be impossible to identify any participants.

To make sure the data collected from your information is accurate, your research and medical records could be inspected by a person or persons authorized by the Research Ethics Board of the CHUS or the institution or by representatives of public authorities. All of these people and groups are bound by confidentiality policies.For safety purposes, in order to be able to reach you quickly if needed, the study investigator will keep your name, surname, contact information and dates of your participation in the study in a separate secured log, for one year after the end of the study.

You have the right to examine your study records in order to check the information collected about you and to correct it, if necessary, for as long as this information is available from the study investigator or the CHUS. However, in order to maintain the scientific integrity of the study, some of this information may be made available to you only once the study has ended.

COMPENSATION

You will not be compensated for your participation in this research study.

PARTICIPANTS' RIGHTS AND INDEMNIFICATION IN CASE OF INJURY

If you suffer any harm whatsoever resulting from your participation in this research study, you will be provided with all the necessary care and services required by your medical condition, at no cost to you.

By agreeing to take part in this study, you do not waive any of your legal rights nor do you release the investigators or the institution where this research study is being conducted from their civil and professional responsibilities.

FUNDING OF THE RESEARCH STUDY

The investigator has received money from the funding agency to conduct this research study.

RESOURCE PERSONS

If you have any question concerning your participation in this study, please refer to the boxed numbers on page 1.

For any question about your rights as a subject participating in this study or if you have comments or wish to file a complaint, you can contact the Commissioner for Complaints and Quality Services of the CIUSSS de l'Estrie-CHUS at the following number: 1-866-917-7903.

MONITORING OF ETHICAL ASPECTS OF THE STUDY

The Research Ethics Board (REB) of the CHUS approved this study and is in charge of its monitoring. Furthermore, we guarantee that any modification to the study protocol or to this information and consent form will be submitted to the REB's approval.

If you wish to contact a member of the REB, please reach the its Support Services at the following number: 819-346-1110, ext. 12856.

CONSENT

I declare having read this Information and Consent Form, especially where the nature of my participation and the extent of the associated risks are concerned. I acknowledge having received explanations about the study, answers to all my questions and having been given enough time to make a decision.

I freely and voluntarily agree to participate in this research study.

Name of participant<br>(please print)	Signature of participant	Date

Name of person who obtains consent<br>(please print)	Signature of person who obtains consent	Date

INVESTIGATOR'S COMMITMENT

I hereby certify that the provisions of this Information and Consent Form were fully explained to the participant, that his/her questions about the research study were answered and that the participant was clearly informed that he/she can withdraw from the study at any time, without prejudice.

I am committed to honour what has been agreed upon in this Information and Consent Form and to give a signed copy of it to the participant. I have participated in the recruitment process of the participant and I confirm that I have informed her/him of my double role as treating physician and investigator.

Name of investigator<br>(please print)	Signature of investigator	Date

CONSENT OF REPRESENTATIVE (ADULTS HAVING SUDDENLY BECOME INCAPABLE)

Due to the fact that Mr./Ms. ______________________________ has suddenly become incapable of giving consent for the reason indicated below, the Civil Code of Quebec authorizes you, as his/her _______________________________ (your relationship with the participant) to give consent for him/her to participate in this research study.
As soon as Mr./Ms ________________________________has recovered enough, we will invite him/her to sign the consent form in order to indicate whether he/she wants to continue participating or not in the study.

REASON WHY THE PARTICIPANT CANNOT GIVE CONSENT:

____

By signing this page, I declare having read this Information and Consent Form. I acknowledge having received explanations about the study, answers to all my questions and having been given enough time to make a decision. I voluntarily agree to the participation of ________________________________ in this study.

____

Name of representative (please print)	Signature of representative	Date

I explained the representative all pertinent aspects of the research and I have answered all his/her questions. I have told him/her that participation in the research study is free and voluntary and that participation can be stopped anytime.
I am committed to honour what has been agreed upon in this Information and Consent Form and to give a signed copy of it to the participant.

____

Name of person who obtains consent (please print)	Signature of person who obtains consent	Date

CONSENT OF REPRESENTATIVE (FOR INCAPABLE ADULTS)

Due to the fact that Mr./Ms. ______________________________ is incapable of giving consent for the reason indicated below, as his/her legal representative you must sign this page in order for the patient to participate in this research study.

REASON WHY THE PARTICIPANT CANNOT GIVE CONSENT:

____

Verbal agreement of the participant able to understand the nature of the project:

Yes: ___ No: ___ N/A: ___ (for the participant incapable of understanding the nature of the project)

____

Name of participant (please print)	Signature of participant (if capable of signing)	Date

____

Name of legal representative (please print)	Signature and title	Date

I explained the representative all pertinent aspects of the research and I have answered all his/her questions. I have told him/her that participation in the research study is free and voluntary and that participation can be stopped anytime. I am committed to honour what has been agreed upon in this Information and Consent Form and to give a signed copy thereof to the participant's representative.

____

Name of person obtaining consent (please print)	Signature of person obtaining consent	Date

SCHEDULE OF STUDY VISITS AND PROCEDURES

**Visit #**	**Screening Visit**			**Follow-up Visit**			**End-of-treatment Visit**
**Treatment Week**	**-2 to 0**	**1**	**5**	**10**	**15**	**20**	**End + 12**
**Visit length (minutes)**	60			30			30
Consent	X						
Medical questionnaire	X						
Questionnaires	X			X			X
Blood draws	X						
Computerized axial tomography (CAT-Scan)	X		X	If needed	If needed	If needed	X
CAT-Scan with injection		X					
Hematoma analysis				If needed			
Medication monitoring				Continuing			
Monitoring of side-effects				Continuing			
