# Supplementary material for: Tranexamic Acid in Chronic Subdural Hematomas (TRACS): study protocol for a randomized controlled trial
Source: Trials. 2016 May 5;17:235. doi: 10.1186/s13063-016-1358-5 (PMC4857422; doi:10.1186/s13063-016-1358-5)
Supplement: Additional file 5: — Data collection forms (original French version). (PDF 334 kb) [file 13063_2016_1358_MOESM5_ESM.pdf]

L'acide tranexamique comme traitement médical des hématomes sous-duraux chroniques

Questionnaire initial

Identification

No sujet ______________________ No dossier ______________________

Nom ______________________ Prénom ______________________

Sexe
☐ Femme
☐ Homme

Date de naissance ______________________

Centre recruteur ______________________ Date du recrutement ______________________

Caractéristiques de base

Dominance	☐ Droitier<br>☐ Gaucher<br>☐ Ambidextre
Consommation d'alcool (nombre par semaine)	______________________
Tabagisme actif	☐ Non<br>☐ Oui
Tabagisme cumulatif (nombre de paquets-années)	______________________
Antécédent d'hématome sous-dural	☐ Non<br>☐ Oui, non-drainé<br>☐ Oui, drainé

Prise récente (moins de 1 mois) d'un médicament antiplaquettaire	☐ Non<br>☐ Oui : ________________
Prise récente (moins de 1 mois) d'un médicament anticoagulant	☐ Non<br>☐ Oui : ________________
Prise récente (moins de 1 mois) d'IECA ou D'ARA	☐ Non<br>☐ Oui : ________________
Prise récente (moins de 1 mois) de corticostéroïde	☐ Non<br>☐ Oui
Convulsion dans la dernière année (nombre)	____________________________
Dérivation de LCR dans la dernière année	☐ Non<br>☐ Oui, ponction lombaire<br>☐ Oui, dérivation ventriculo-péritonéale<br>☐ Oui, autre : ______________
Antécédent de procédure neurochirurgicale récente (moins de 1 mois avant le début des symptômes)	☐ Non<br>☐ Oui, procédure extra-crânienne<br>☐ Oui, procédure intra-crânienne
Histoire de traumatisme récent (moins de 1 mois avant le début des symptômes)	☐ Non<br>☐ Oui, mineur (pas de diagnostic)<br>☐ Oui, majeur (TCC léger ou pire diagnostiqué par un MD)
Tout autre facteur de risque d'hématome sous-dural chronique jugé pertinent	____________________________

Présentation clinique

Contexte de la consultation	☐ Consultation élective en clinique externe<br>☐ Consultation non-urgente à l'urgence ou sur un étage<br>☐ Consultation urgente
Symptôme **principal** à la présentation<br>(ne cocher qu'un seul symptôme)	☐ Asymptomatique<br>☐ Céphalées<br>☐ Confusion<br>☐ Somnolence<br>☐ Chute ou trouble d'équilibre<br>☐ Faiblesse d'un membre<br>☐ Trouble du langage<br>☐ Trouble de mémoire<br>☐ Nausées/Vomissements<br>☐ Trouble visuel<br>☐ Convulsion<br>☐ Autre
Date du début approximatif des symptômes	______________________
Score MMSE au recrutement	______________________
Score mBI au recrutement	______________________
Score EQ-5D détaillé (XXXXX) au recrutement	______________________
Score EQ-5D VAS au recrutement	______________________
Plan initial de traitement	☐ Conservateur<br>☐ Drainage électif<br>☐ Drainage urgent (ne pouvant pas attendre au lendemain)

EXAMEN DE FOLSTEIN SUR L'ÉTAT MENTAL

DT9088

Nom de l'établissement ______________________________

A) Orientation

	COTE MAXIMALE	COTE DU SUJET
Demander au sujet :		
**1- Quel est** l'année ___ le mois ___ le jour ___ le jour de la semaine : D L M Me J V S<br>La saison : printemps ☐ été ☐ automne ☐ hiver ☐	**5**	( )
**2- Où sommes-nous** Province ___ Pays ___<br>Ville, village ___ Lieu (hôpital, cabinet, maison, etc.) ___ Étage ___	**5**	( )

B) Enregistrement

3- Mentionner un des groupes de 3 mots suivants; prendre une seconde pour prononcer chaque mot :

chemise, bleu, honnêteté ( )
ou
chaussure, brun, modestie ( )
ou
chandail, blanc, charité ( )

COTE MAXIMALE : **3** — COTE DU SUJET : ( )

Par la suite, demander au sujet de répéter les 3 mots choisis.
Donner 1 point pour chaque bonne réponse au 1er essai. Répéter l'exercice jusqu'à ce que le sujet retienne les 3 mots.
Compter le nombre d'essais et le noter. Pour information seulement.

Nombre d'essais : ______

C) Attention et calcul (cocher l'un ou l'autre test)

4- ☐ Demander au sujet de faire la soustraction par intervalles de 7 à partir de 100 :

100 − 7 = ( ) 93 − 7 = ( ) 86 − 7 = ( ) 79 − 7 = ( ) 72 − 7 = ( ) 65.

Donner 1 point pour chaque bonne réponse.

OU

☐ Demander au sujet d'épeler le mot « MONDE » à l'envers. (EDNOM) : ___ ___ ___ ___ ___ (écrire les lettres)

COTE MAXIMALE : **5** — COTE DU SUJET : ( )

D) Rétention mnésique

5- Demander au sujet de répéter les 3 mots déjà mentionnés :

chemise, bleu, honnêteté ( )
ou
chaussure, brun, modestie ( )
ou
chandail, blanc, charité ( )

COTE MAXIMALE : **3** — COTE DU SUJET : ( )

E) Langage

	COTE MAXIMALE	COTE DU SUJET
6- Montrer au sujet un crayon ( ) une montre ( ) et lui demander de nommer l'objet.	**2**	( )
7- Demander au sujet de répéter la phrase suivante : **« Pas de si ni de mais »**.	**1**	( )
8- Demander au sujet d'obéir à un ordre en 3 temps : **« Prenez ce papier de la main droite ou gauche, pliez-le en deux et redonnez-le moi ».**	**3**	( )

N.B. : *Demander au sujet droitier de prendre de la main gauche et vice versa. Prendre garde de tendre la main; éviter les indices non-verbaux.*

Traduction et adaptation française non validées du « Mini-Mental State » de Folstein, M.F., Folstein, S.E., Mc Hugh, P.R. : « Mini-Mental State : A practical method for grading the cognitive state of patients for the clinician ». J. Psychiatr Res, 12 : 189-198, 1975, à partir des travaux du centre de gériatrie Hôpital d'Youville Sherbrooke de Québec.

(suite au verso)

Nom de l'usager	N° de dossier

E) Langage (suite)

	COTE MAXIMALE	COTE DU SUJET
9- Demander au sujet de lire et de suivre l'instruction suivante : **« FERMEZ VOS YEUX »**	1	( )
10- Demander au sujet d'écrire une phrase : ______ (sujet, verbe, sans égard aux fautes)	1	( )

F) Praxie de construction

11- Demander au sujet de copier le dessin suivant :

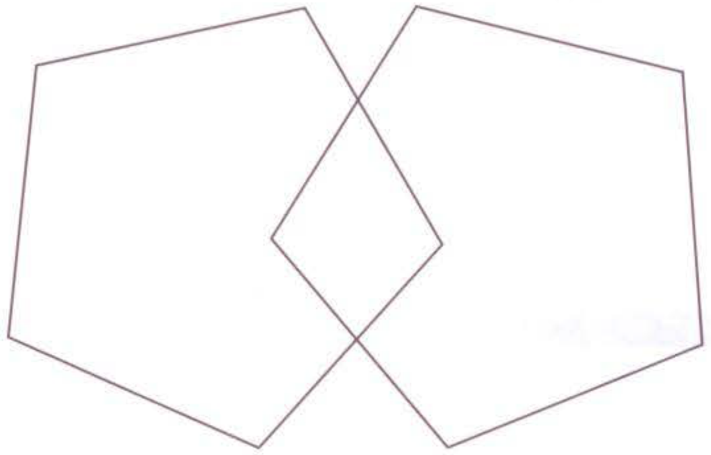

	1	( )

COTE MAXIMALE : 30

COTE DU SUJET : ______

Interprétation des scores :
Un score de moins de 24 points à l'examen de Folstein sur l'état mental détermine une altération des fonctions cognitives. Ce test ne permet cependant pas de faire un diagnostic étiologique.

Nombre d'années de scolarité du sujet : ______ Évaluer le niveau de conscience du sujet : vigilant ☐ somnolent ☐

En cas d'incapacité du sujet à être évalué, spécifier : ______

Indiquer les conditions ayant pu influencer l'évaluation ______

Date | Année | Mois | Jour |

Signature ______

EQ-5D-5L

Pour chaque rubrique, veuillez cocher UNE case, celle qui décrit le mieux votre santé AUJOURD'HUI.

MOBILITÉ

Je n'ai aucun problème pour me déplacer à pied ❑

J'ai des problèmes légers pour me déplacer à pied ❑

J'ai des problèmes modérés pour me déplacer à pied ❑

J'ai des problèmes sévères pour me déplacer à pied ❑

Je suis incapable de me déplacer à pied ❑

AUTONOMIE DE LA PERSONNE

Je n'ai aucun problème pour me laver ou m'habiller tout(e) seul(e) ❑

J'ai des problèmes légers pour me laver ou m'habiller tout(e) seul(e) ❑

J'ai des problèmes modérés pour me laver ou m'habiller tout(e) seul(e) ❑

J'ai des problèmes sévères pour me laver ou m'habiller tout(e) seul(e) ❑

Je suis incapable de me laver ou de m'habiller tout(e) seul(e) ❑

ACTIVITÉS COURANTES *(exemples: travail, études, travaux domestiques, activités familiales ou loisirs)*

Je n'ai aucun problème pour accomplir mes activités courantes ❑

J'ai des problèmes légers pour accomplir mes activités courantes ❑

J'ai des problèmes modérés pour accomplir mes activités courantes ❑

J'ai des problèmes sévères pour accomplir mes activités courantes ❑

Je suis incapable d'accomplir mes activités courantes ❑

DOULEURS / INCONFORT

Je n'ai ni douleur ni inconfort ❑

J'ai des douleurs ou un inconfort léger(ères) ❑

J'ai des douleurs ou un inconfort modéré(es) ❑

J'ai des douleurs ou un inconfort sévère(s) ❑

J'ai des douleurs ou un inconfort extrême(s) ❑

ANXIÉTÉ / DÉPRESSION

Je ne suis ni anxieux(se) ni déprimé(e) ❑

Je suis légèrement anxieux(se) ou déprimé(e) ❑

Je suis modérément anxieux(se) ou déprimé(e) ❑

Je suis sévèrement anxieux(se) ou déprimé(e) ❑

Je suis extrêmement anxieux(se) ou déprimé(e) ❑

Canada (French) © 2009 EuroQol Group EQ-5D™ is a trade mark of the EuroQol Group

- Nous aimerions savoir dans quelle mesure votre santé est bonne ou mauvaise AUJOURD'HUI.

- Cette échelle est numérotée de 0 à 100.

- 100 correspond à la <u>meilleure</u> santé que vous puissiez imaginer.
  0 correspond à la <u>pire</u> santé que vous puissiez imaginer.

- Veuillez faire un X sur l'échelle afin d'indiquer votre état de santé AUJOURD'HUI.

- Maintenant, veuillez noter dans la case ci-dessous le chiffre que vous avez coché sur l'échelle.

VOTRE SANTÉ AUJOURD'HUI = ☐

Date: ______________________________

La meilleure santé que vous puissiez imaginer

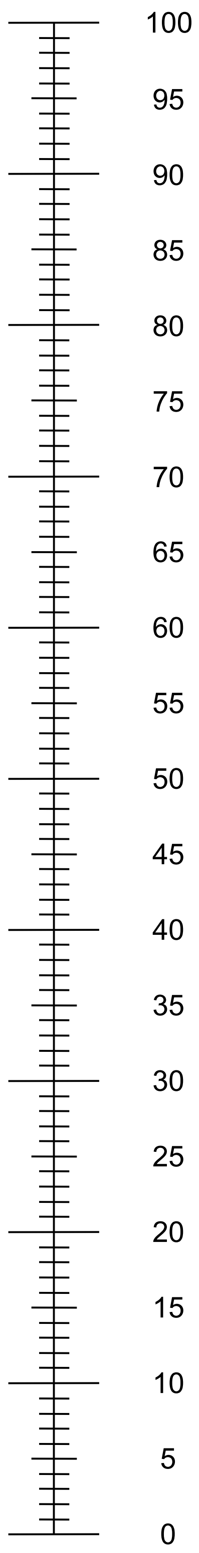

La pire santé que vous puissiez imaginer

Canada (French) © 2009 EuroQol Group EQ-5D™ is a trade mark of the EuroQol Group

EQ-5D-5L

Under each heading, please tick the ONE box that best describes your health TODAY.

MOBILITY

I have no problems in walking about ❑
I have slight problems in walking about ❑
I have moderate problems in walking about ❑
I have severe problems in walking about ❑
I am unable to walk about ❑

SELF-CARE

I have no problems washing or dressing myself ❑
I have slight problems washing or dressing myself ❑
I have moderate problems washing or dressing myself ❑
I have severe problems washing or dressing myself ❑
I am unable to wash or dress myself ❑

USUAL ACTIVITIES *(e.g. work, study, housework, family or leisure activities)*

I have no problems doing my usual activities ❑
I have slight problems doing my usual activities ❑
I have moderate problems doing my usual activities ❑
I have severe problems doing my usual activities ❑
I am unable to do my usual activities ❑

PAIN / DISCOMFORT

I have no pain or discomfort ❑
I have slight pain or discomfort ❑
I have moderate pain or discomfort ❑
I have severe pain or discomfort ❑
I have extreme pain or discomfort ❑

ANXIETY / DEPRESSION

I am not anxious or depressed ❑
I am slightly anxious or depressed ❑
I am moderately anxious or depressed ❑
I am severely anxious or depressed ❑
I am extremely anxious or depressed ❑

Canada (English) © 2009 EuroQol Group EQ-5D™ is a trade mark of the EuroQol Group

- We would like to know how good or bad your health is TODAY.
- This scale is numbered from 0 to 100.
- 100 means the best health you can imagine.
  0 means the worst health you can imagine.
- Mark an X on the scale to indicate how your health is TODAY.
- Now, please write the number you marked on the scale in the box below.

YOUR HEALTH TODAY = ☐

Date: ______________________________

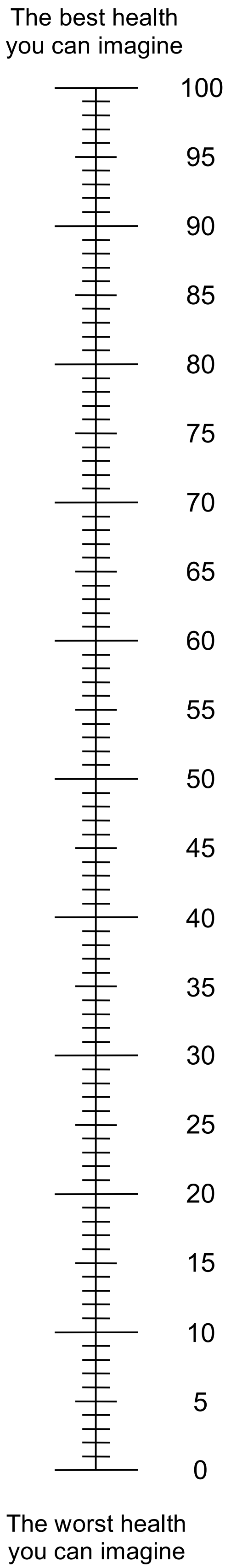

Canada (English) © 2009 EuroQol Group EQ-5D™ is a trade mark of the EuroQol Group

Modified Barthel Index

Grille d'évaluation

No sujet ____________________ Date d'administration ____________________

	1<br>Incapable	2<br>Tentatives non-sécuritaires	3<br>Réussit avec aide modérée	4<br>Réussit avec aide minimale	5<br>Autonome
Hygiène du visage (dents, barbe)	0	1	3	4	5
Prendre son bain	0	1	3	4	5
Aller à la toilette	0	2	5	8	10
Continence urinaire	0	2	5	8	10
Continence fécale	0	2	5	8	10
S'alimenter	0	2	5	8	10
S'habiller	0	2	5	8	10
Monter des escaliers	0	2	5	8	10
Marcher	0	3	8	12	15
Se déplacer en chaise roulante (si incapable de marcher)	0	1	3	4	5
Transférer au lit	0	3	8	12	15

Total : ________ / 100

CENTRE DE RECHERCHE | CHUS

ÉTUDE TRACS – 14-213

L'acide tranexamique comme traitement médical des hématomes sous-duraux chroniques

Protocole opératoire

No sujet ___________________________________

Procédure réalisée à DROITE

Contexte de la chirurgie	☐ Aucune chirurgie effectuée à droite<br>☐ Chirurgie élective<br>☐ Chirurgie d'urgence (qui n'aurait pas pu attendre au lendemain)
Date de la chirurgie	________________________________
Type de chirurgie	☐ Trépanation simple à droite<br>☐ Trépanation double à droite<br>☐ Craniotomie droite<br>☐ Autre : ______________________
Lieu de la chirurgie	☐ Bloc opératoire<br>☐ Draînage au chevet
Installation de drain	☐ Non<br>☐ Oui, drain sous-dural droit<br>☐ Oui, drain sous-galéal droit<br>☐ Oui, autre : __________________
Orientation du drain	☐ Aucun drain<br>☐ Extrémité en antérieur<br>☐ Extrémité en postérieur
Irrigation intra-opératoire	☐ Non<br>☐ Oui
Mobilisation post-opératoire	☐ Repos au lit<br>☐ Mobilisation selon tolérance

Procédure réalisée à GAUCHE

Contexte de la chirurgie	☐ Aucune chirurgie effectuée à gauche<br>☐ Chirurgie élective<br>☐ Chirurgie d'urgence (qui n'aurait pas pu attendre au lendemain)
Date de la chirurgie	________________________________
Type de chirurgie	☐ Trépanation simple à gauche<br>☐ Trépanation double à gauche<br>☐ Craniotomie gauche<br>☐ Autre : ______________________
Lieu de la chirurgie	☐ Bloc opératoire<br>☐ Draînage au chevet
Installation de drain	☐ Non<br>☐ Oui, drain sous-dural gauche<br>☐ Oui, drain sous-galéal gauche<br>☐ Oui, autre : __________________
Orientation du drain	☐ Aucun drain<br>☐ Extrémité en antérieur<br>☐ Extrémité en postérieur
Irrigation intra-opératoire	☐ Non<br>☐ Oui
Mobilisation post-opératoire	☐ Repos au lit<br>☐ Mobilisation selon tolérance

Signature ______________________________ Date ______________________________

Questionnaire de suivi

No sujet ______________________________

Résolution du symptôme initial	☐ Non<br>☐ Partiellement<br>☐ Complètement
Usage de stéroïdes depuis la dernière visite	☐ Non<br>☐ Oui : dose + dates : ____________
Usage d'antiplaquettaires depuis la dernière visite	☐ Non<br>☐ Oui : doses + dates : ___________
Score MMSE	______________________________
Score mBI	______________________________
Score EQ-5D (détaillé + VAS)	______________________________
Score GOSE (uniquement pour le suivi final)	______________________________

Surveillance évènements indésirables

Draînage chirurgical depuis la dernière visite	☐ Non<br>☐ Oui, planifié, date :______________<br>☐ Oui, non-planifié, date :__________
Thrombophlébite diagnostiquée depuis la dernière visite	☐ Non<br>☐ Oui, date : ____________________
Embolie pulmonaire diagnostiquée depuis la dernière visite	☐ Non<br>☐ Oui, date : ____________________
ICT diagnostiquée depuis la dernière visite	☐ Non<br>☐ Oui, date : ____________________
AVC diagnostiquée depuis la dernière visite	☐ Non<br>☐ Oui, date : ____________________
Pseudo-néphrolithiase diagnostiquée depuis la dernière visite	☐ Non<br>☐ Oui, date : ____________________
Changement de la vision depuis la dernière visite	☐ Non<br>☐ Oui, date et détails : ____________
Autre complication potentiellement attribuable à l'administration d'acide tranexamique (et date)	______________________________

Signature ______________________________ Date ______________________________
